# An Evaluation of *Arabidopsis thaliana* Hybrid Traits and Their Genetic Control

**DOI:** 10.1534/g3.111.001156

**Published:** 2011-12-01

**Authors:** Siobhan Moore, Lewis Lukens

**Affiliations:** Department of Plant Agriculture, University of Guelph, Guelph, Ontario, Canada, N1G 2W1

**Keywords:** heterosis, *FRIGIDA*, *FLOWERING LOCUS C*, diallel, *Arabidopsis thaliana*

## Abstract

Heterosis is an important phenomenon in agriculture. However, heterosis often greatly varies among hybrids and among traits. To investigate heterosis across a large number of traits and numerous genotypes, we evaluated 12 life history traits on parents and hybrids derived from five *Arabidopsis thaliana* ecotypes (Col, L*er*-0, Cvi, Ws, and C24) by using a complete diallel analysis containing 20 hybrids. Parental contributions to heterosis were hybrid and trait specific with a few reciprocal differences. Most notably, C24 generated hybrids with flowering time, biomass, and reproductive traits that often exceeded high-parent values. However, reproductive traits of C24 and Col hybrids and flowering time traits of C24 and L*er* hybrids had no heterosis. We investigated whether allelic variation at flowering time genes *FRIGIDA* (*FRI*) and *FLOWERING LOCUS C* (*FLC*) could explain the genotype- and trait-specific contribution of C24 to hybrid traits. We evaluated both Col and L*er* lines introgressed with various *FRI* and *FLC* alleles and hybrids between these lines and C24. Hybrids with functional *FLC* differed from hybrids with nonfunctional *FLC* for 21 of the 24 hybrid-trait combinations. In most crosses, heterosis was fully or partially explained by *FRI* and *FLC*. Our results describe the genetic diversity for heterosis within a sample of *A. thaliana* ecotypes and show that *FRI* and *FLC* are major factors that contribute to heterosis in a genotype and trait specific fashion.

The occurrence of heterosis, or hybrid vigor, is a phenomenon in which the hybrid offspring between two parental lines has trait values that surpass the trait values of the parents (Crow 1948). The phenomenon of heterosis was noted as far back as 1876 by Charles Darwin (Shull 1908). Heterosis is often referred to in traits associated with vigor, such as size, yield, and reproductive success (Lippman and Zamir 2007), but the term is also used more broadly to describe any trait for which a hybrid exceeds parental levels (Xiao *et al.* 1995). Heterosis in both the narrow and broad senses is prevalent in numerous species and has been critical for agricultural productivity for many decades (Whaley 1944).

*Arabidopsis thaliana* offers a tractable model system to investigate the genetic basis of heterosis. Although *A. thaliana* is autogamous, and heterosis is predicted to be low, heterosis has been found to be widespread among various *A. thaliana* hybrids. Heterosis has been reported for the rate of early biomass accumulation and for yield-related traits, including biomass yield, number of seeds, and 1000-seed weight (Barth *et al.* 2003; Meyer *et al.* 2004; Kusterer *et al.* 2007a; Kusterer *et al.* 2007b).

In most studies within *Arabidopsis* investigators have focused on elucidating the genetic basis for heterosis by examining one or a small number of traits or by using a small number of crosses. However, heterosis is variable across traits and across genotypes (Barth *et al.* 2003; Meyer *et al.* 2004; Syed and Chen 2005; Springer and Stupar 2007; Stupar *et al.* 2008; Flint-Garcia *et al.* 2009). One hybrid trait may exhibit heterosis, whereas a second trait may have a lower level of heterosis, be unaffected, or be lower than parental trait values (Barth *et al.* 2003; Syed and Chen 2005; Springer and Stupar 2007; Stupar *et al.* 2008; Flint-Garcia *et al.* 2009). One genotype may exhibit extensive heterosis for a trait, whereas another genotype may have little to no heterosis for the same trait (Barth *et al.* 2003; Meyer *et al.* 2004; Stupar *et al.* 2008). Furthermore, reciprocal hybrids may differ in trait expression. A number of *A. thaliana* reciprocal hybrids, including Col-0 x C24 and Cvi x L*er*, differed for biomass, seed size, and seed yield. These reciprocal differences have been attributed to nonmaternal genetic factors and to maternal nuclear or cytoplasmic effects (Alonso-Blanco *et al.* 1999; Barth *et al.* 2003).

Heterosis within *A. thaliana* has been attributed to dominance, overdominance, pseudo-overdominance, and/or epistasis depending on the traits and the crosses examined. Heterosis for viability in a cross between the Niederzenz and Landsberg ecotypes of *A. thaliana* was attributed to overdominance, *i.e.* F2 progeny homozygous for one locus had 50% lower viability than heterozygotes (Mitchell-Olds 1995). Single-locus heterosis attributable to overdominance has been observed for stem length, total number of buds, flowers and fruit, and fresh and dry weight (Rédei 1962). Kusterer *et al.* (2007a) and Kusterer *et al.* (2007b) examined C24 x Col-derived recombinant inbred lines crossed to both parents and the F1 and found that heterosis for biomass-related traits within 29 days of sowing was caused by dominance, overdominance or pseudo-overdominance, and epistasis. The authors of an analysis of early growth in C24 x Col near isogenic line hybrids found a significant role for epistasis (Melchinger *et al.* 2007).

The flowering time genes *FRIGIDA* (*FRI*) and *FLOWERING LOCUS C* (*FLC*) interact epistatically and have a large effect on flowering time. Genotypes with functional alleles at both the *FRI* and *FLC* loci flower much later than genotypes that carry only one functional allele when plants are not vernalized (Lee *et al.* 1994; Sanda and Amasino 1995). Late flowering in interspecific allotetraploids between *A. thaliana* and *A. arenosa* has also been attributed to functional alleles of *FRI* and *FLC* because a functional *A. arenosa FRI* allele *trans*-activated an *A. thaliana FLC* allele (Wang *et al.* 2006). *FRI* and *FLC* may affect other traits because mutations in flowering time genes can cause changes in leaf number, the number of axillary flowering shoots, final height, silique number, total number of seeds, floral organ development, as well as other traits (Tienderen *et al.* 1996; Koornneef *et al.* 1998; Alonso-Blanco *et al.* 1999).

In this study, we first investigated how heterosis varies across 12 diverse traits measured in 20 hybrids derived from five parental genotypes. We used a complete diallel design to determine the contribution of each genotype on each trait by estimating general (additive) and specific (nonadditive) combining abilities as well as reciprocal effects. Second, we determined the degree to which genetic variation at *FRI* and *FLC* explains a number of hybrid traits across different genotypes. We suggest that genes or genotypes interpreted as having additive effects may interact nonadditively to generate hybrid trait variation.

## Materials and Methods

### Plant growth conditions, trait measurements

Plants were grown under long day conditions with 16 hr (07:00–23:00) of ~150 µmol m^-2^ s^-1^ light and 8 hr of dark at a constant temperature of 23°. Eleven traits were measured, and one reproductive trait, total seed per plant, was estimated (Table 1). Days to bolting, days to flowering, and days to mature seed were the three flowering time traits. Rosette diameter at bolting, shoot biomass at death, and final height at death were the three biomass traits. Total number of siliques, average silique length, average number of seeds per silique, and total number of seeds per plant were the four reproductive traits. The total number of seeds for each plant was estimated by multiplying the total number of siliques by the average number of seeds per silique. We also measured height at flowering and lifespan. Death was defined as the day the last silique matured and the plant was no longer producing new branches. Additional details on growth conditions and trait measurements are given in the supporting information (File S1).

**Table 1:** The 12 traits measured in diallel and introgression experiments

Trait
Days to bolting (A)
Days to flowering (B)
Days to mature Seed (C)
Rosette diameter (D)
Shoot biomass (E)
Final height (F)
Total number of siliques (G)
Total number of seeds (H)
Average silique length (I)
Average number of seeds per silique (J)
Height at flowering (K)
Lifespan (L)

### Diallel plant material and experimental design

Five *A. thaliana* parental lines, *i.e.* Columbia (Col), Wassilewskija (Ws), Landsberg *erecta* (L*er*), Cape Verde Islands (Cvi), and C24, were mated by the use of a complete diallel mating design. The diallel consisted of 20 hybrids, including reciprocals, and the manually crossed five parental genotypes, for a total of 25 lines. The diallel plants were examined by the use of a split plot design consisting of four split-plots with 25 cells each that formed two whole plot replicates. The whole-plot factor, density, had two levels, high and low. Density stress was imposed upon plants as an environmental effect because heterosis may be more evident in conditions of stress (Tollenaar and Wu 1999). The high-density treatment consisted of the plant of interest located in the center of a 3.5-inch pot surrounded by four L*er* parent plants (2 cm spacing) within the same cell. The low-density treatment consisted of only the genotype of interest centered in a pot. The split-plot factor, genotype, with 25 levels was randomly assigned to the 25 cells within each flat. Each pot was re-randomized within its flat every 6 days to ensure homogeneity within the flat and to eliminate edge effects. Rearranging ceased approximately 3 months after the start of the experiment. Cleaved amplified polymorphic sequences marker analysis of DNA extracted from each hybrid and inbred plant was used to confirm plant genotypes. Manual pollinations were used to produce all seed. Among the 100 plants, three were not of the expected genotype and were removed (two Ws x Cvi and one Col x Ws).

### Introgression plant material and experimental design

To investigate the effects of *FRI* and *FLC* in nearly isogenic backgrounds, we used lines homozygous for the four combinations of functional and nonfunctional *FRI* and *FLC* alleles. Each *FRI* and *FLC* combination was obtained in the Col background and the L*er* background. For simplicity, each line has been given a short notation to refer to its *FRI* and *FLC* alleles. A +/+ indicates a line with a functional *FRI* and a functional *FLC* allele, whereas +/− indicates a line with a functional *FRI* allele and a nonfunctional *FLC* allele. A −/+ indicates a genotype with a nonfunctional *FRI* allele and a functional *FLC* allele, whereas −/− indicates a genotype with nonfunctional alleles for both *FRI* and *FLC*. We investigated two L*er* −/+ genotypes. In one, the functional *FLC* was introgressed from Col. In the other, it was introgressed from Sf2. Additional details on the lines are given in supporting information. To investigate the effect of *FLC* in hybrid backgrounds, we generated (C24 x Col) +/+ and (C24 x L*er*) +/+ F1 hybrids and (C24 x Col) +/− and (C24 x L*er*) +/− F1 hybrids.

Introgression lines and their hybrids with C24 were evaluated by the use of a randomized complete block design with six blocks. Each of the nine isogenic genotypes [Col −/−, −/+, +/−, and +/+, as well as L*er* −/−, −/+ (Sf2), −/+ (Col), +/−, and +/+], the four hybrid genotypes [(C24 x Col) +/+, (C24 x Col) +/−, (C24 x L*er*) +/+, and (C24 x L*er*) +/−], and the C24 +/− parental genotype were randomly placed within each block for a total of 84 plants (14 plants per block with six blocks).

### Statistical analyses

To partition phenotypic variation to density and genotypic effects, data from the five parent complete diallel design was analyzed with DIALLEL-SAS05 via the use of Griffing’s method 1 (Griffing 1956; Zhang and Kang 1997; Zhang *et al.* 2005). Genotypes were treated as fixed effects. Variances attributed to interactions between density and genotype were also determined. Genotypic variance was partitioned into general combining ability (GCA), specific combining ability (SCA), and reciprocal effects, which were further partitioned to maternal and nonmaternal effects.

We also used a split-plot analysis to analyze diallel data to estimate hybrid deviations from mid-, low-, and high-parent values and to perform contrasts between genotypes. Finally, we used the following equation to calculate heterosis of the diallel hybrids:

$$\%\text{Heterosis}=\frac{\left(\mathrm{F1}-X\right)}{X}\times 100$$

where F1 is the mean trait value of a specific hybrid. For mid-parent heterosis (MPH), X is the mean trait value of the two parents of the hybrid; for high-parent heterosis (HPH), X is the mean trait value of the high parent.

The introgression line and hybrid introgression line experiment was analyzed with a randomized complete block design model. Within the introgression line analysis, contrasts were performed among Col and L*er* introgression lines to evaluate the effects of *FRI* and *FLC* on trait values within the inbred genotypes. For the hybrids between C24 and the introgression lines, we contrasted the hybrid with *FLC* to the hybrid without *FLC* to determine the effect of *FLC* when a functional *FRI* was present.

A type I error rate of alpha = 0.01 was used to define statistical significance unless otherwise stated. Analyses were performed with SAS 9.1 (SAS Institute Inc., Cary, NC, USA 2002-2003) general linear model procedure. Data used in all analyses are given in the supporting information (Table S2, Table S3, Table S4, Table S5, and Table S6).

## Results

### Analysis of diallel components

To determine the parental genotypic contributions to *A. thaliana* hybrid traits, we evaluated 12 traits by using a complete diallel design comprising five parental ecotypes (Col, Cvi, L*er*, Ws, and C24) and 20 F1 hybrids. Genotype explained a highly significant (*P* < 0.0001) proportion of the variation for every trait except plant lifespan (Table 2). The genetic components of variation for rosette diameter and days to bolting, flowering and mature seed were especially high. The mean square estimates for genotype were over 60x the mean square errors for these traits (Table 2). The genotype mean square estimates for the remaining 7 traits ranged from 5 to 32 times the mean square error.

**Table 2:** Variance partitions for the 12 traits measured in the complete diallel

Source	df	Mean Square											
		A	B	C	D	E	F	G	H	I	J	K	L
Density	1	10.24	36.00	39.27	222.01	23.00*^a^*	24,263.25*^a^*	2,075.33*^b^*	36,931.91*^a^*	2.02	4.19	330.03	15.73
REP (density)	2	8.84	9.65	16.47	43.81	0.12	989.85	5.50	979.41	1.71*^a^*	82.33*^b^*	86.41	503.76
Genotype	24	407.04*^c^*	367.86*^c^*	416.92*^c^*	8,706.01*^c^*	14.87*^c^*	25,897.52*^c^*	404.80*^c^*	10,417.07*^c^*	8.43*^c^*	187.08*^c^*	2,590.67*^c^*	307.46
GCA	4	1,355.18*^c^*	1,184.01*^c^*	1,349.87*^c^*	24,984.62*^c^*	50.68*^c^*	96,442.98*^c^*	1,425.00*^c^*	31,663.85*^c^*	17.01*^c^*	604.52*^c^*	5,950.88*^c^*	383.80
SCA	10	412.32*^c^*	386.60*^c^*	432.89*^c^*	10,608.89*^c^*	14.68*^c^*	21,913.21*^c^*	322.41*^c^*	10,629.11*^c^*	11.83*^c^*	172.42*^c^*	2,737.03*^c^*	267.08
REC	10	22.49*^b^*	22.65*^b^*	27.77*^b^*	291.68*^a^*	0.75	1,663.65	79.11*^a^*	1,706.30*^a^*	1.59*^b^*	34.77*^b^*	1,100.24*^a^*	317.31
MAT	4	23.79*^b^*	24.35*^b^*	32.48*^b^*	125.24	0.73	2,710.58	98.93*^a^*	2,702.48*^a^*	1.96*^b^*	31.46*^a^*	1,994.68*^b^*	519.99
NMAT	6	21.62*^b^*	21.52*^b^*	24.62*^b^*	402.63*^a^*	0.77	965.69	65.90	1,042.19	1.35*^a^*	36.98*^b^*	503.95	182.19
Genotype density*^a^*	24	4.74	4.67	6.08	194.39	0.81*^a^*	2,178.80	37.39	668.40	0.40	9.67	426.70	339.52
Error	48	4.44	4.78	6.64	134.66	0.46	1,475.34	34.05	836.28	0.44	11.15	473.17	267.92

A, days to bolting; B, days to flowering; C, days to mature seed; D, rosette diameter; E, shoot biomass; F, final height; G, total number of siliques; GCA, general combining ability ; H, total number of seeds; I, silique length; J, average number of seeds per silique; K, height at flowering; L, lifespan; MAT, material; NMAT, nonmaterial; SCA, specific combining ability. Rep, repetition; REC, reciprocal.

a *P* < 0.05, *^b^P* < 0.01, *^c^P* < 0.0001.

**Figure 1:**
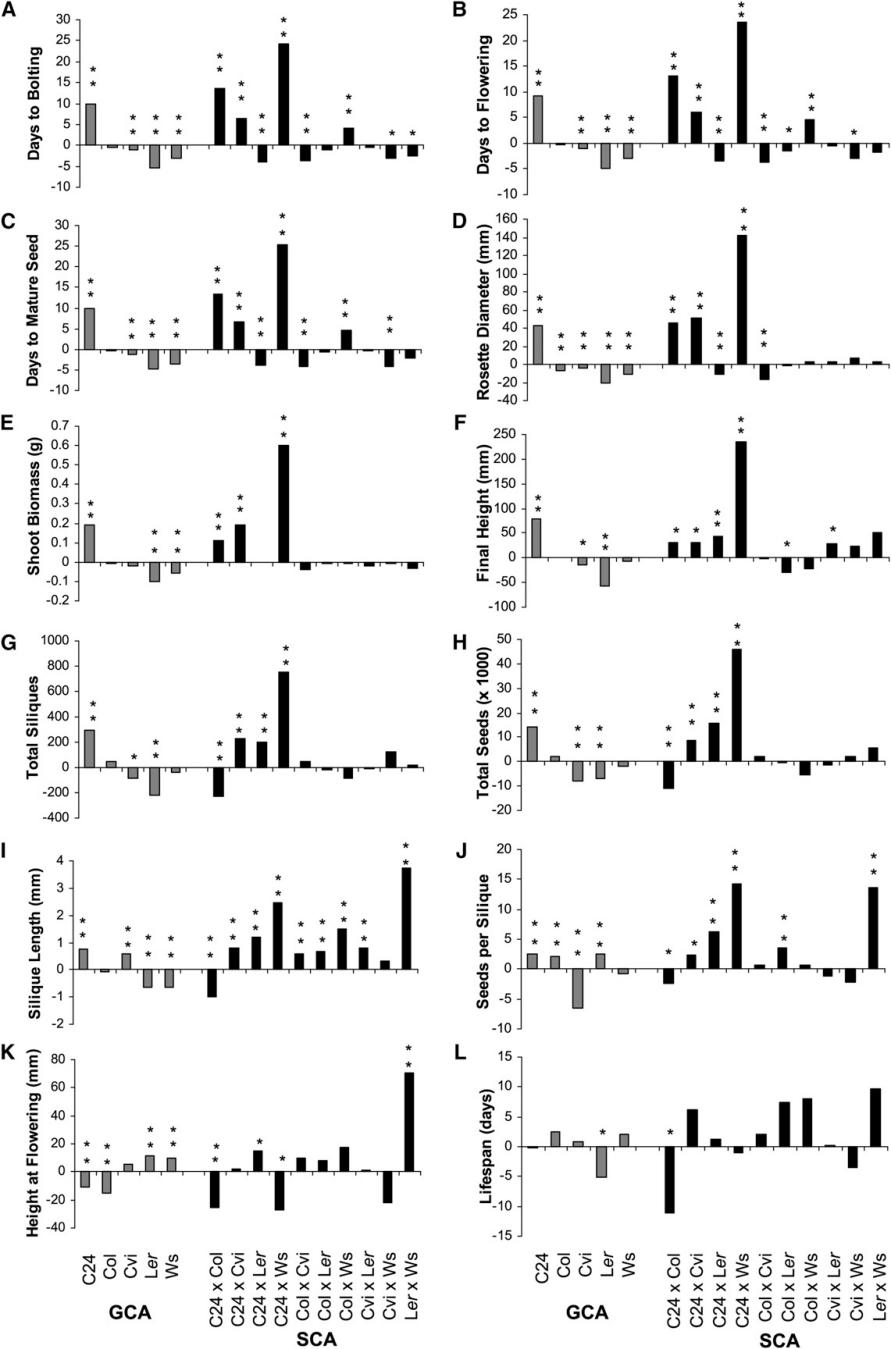
GCA estimates for parental genotypes and hybrid SCA estimates. The parental GCA estimates and the hybrid SCA estimates for all 12 traits measured in the diallel. (A) Days to bolting; (B) days to flowering; (C) days to mature seed; (D) rosette diameter; (E) shoot biomass; (F) final height; (G) total number of siliques; (H) total number of seeds; (I) silique length; (J) number of seeds per silique; (K) height at flowering; (L) lifespan. Gray bars show the GCA estimates and black bars show the SCA estimates. ^*^P < 0.05, ^**^P < 0.01.

**Figure 2:**
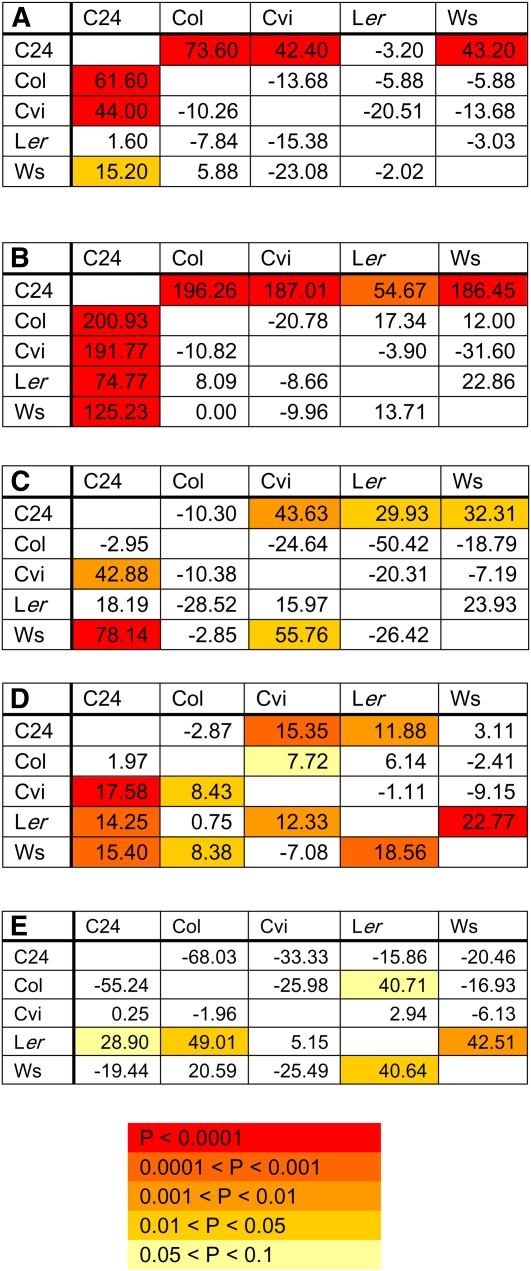
The percent HPH for each hybrid for a number of traits. The color represents the significance level of the HPH estimate. The value in the cell is the percent HPH. (A) The percentage of HPH for days to flower. (B) The percentage of HPH for rosette diameter. (C) The percentage of HPH for total number of siliques. (D) The percentage of HPH for silique length. (E) The percentage of HPH for height at flowering. The maternal genotype is on the vertical axis and the paternal genotype is on the horizontal axis.

**Figure 3:**
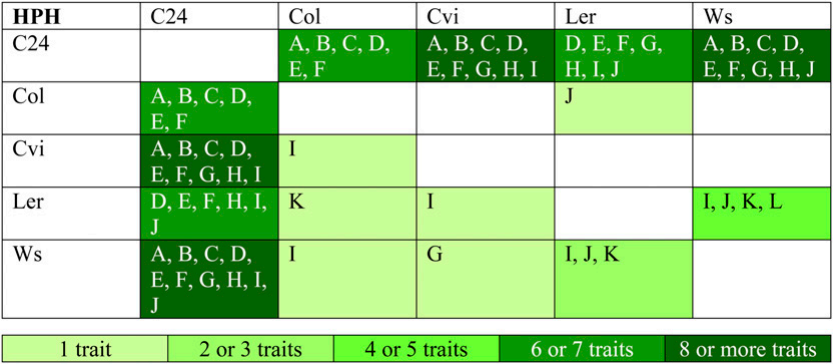
HPH summary of diallel traits. The percent heterosis was calculated for each genotype and for each trait for all hybrids within the diallel experiment. The letter in the cell indicates which traits have HPH at *P* < 0.05 (A, days to bolting; B, days to flowering; C, days to mature seed; D, rosette diameter; E, shoot biomass; F, final height; G, total number of siliques; H, total number of seeds; I, silique length; J, number of seeds per silique; K, height at flowering; L, lifespan). The color gradient from light to dark represents a low to high number of traits with HPH for the genotype. The maternal genotype is on the vertical axis and the paternal genotype is on the horizontal axis.

For the 11 traits that had significant genotypic effects, the variance of GCA, that is, the difference between the average trait value of a specific parent’s offspring and the average trait value of the population, was highly significant (*P* < 0.0001; Table 2). Of the five parental lines, C24 consistently had the largest effect on traits, having positive GCA estimates for 10 of 12 traits (Figure 1). This was also seen in heterosis measurements (*e.g.,* Figure 2). Of the 75 hybrid-trait combinations with significant HPH, 62 were in hybrids that had C24 as a parent (Figure 2, A–D, Figure 3, and Figure S1). For eight of the traits (days to bolting, flowering and mature seed; rosette diameter; shoot biomass; final height; total siliques; and total seeds), HPH was predominately restricted to hybrids where C24 was used as a parent (Figure 2, A–C, Figure 3, and Figure S1). Interestingly, height at flowering was often shorter in the late flowering C24 hybrids than in the early flowering parental lines (Figure 2E).

Although the C24 parent had a large, positive effect on many hybrids, this effect varied greatly. Largely because of the different effects of C24 on hybrids, the SCA, the estimated deviation of the trait value of an individual cross from the sum of the parental GCA effects, explained a highly significant proportion (*P* < 0.0001) of the genetic variance for all 11 traits (Table 2). For most flowering time traits (days to bolting, flowering, and mature seed) and biomass traits (rosette diameter, shoot biomass, final height), C24 hybrids with Ws had very high SCA estimates, C24 hybrids with Col and Cvi had high SCA estimates, and C24 hybrids with L*er* had negative SCA estimates (Figure 1, A−F). The effect of C24 on hybrids also varied for reproductive traits. Almost all of the reproductive traits in hybrids between C24 with Cvi, L*er*, and Ws (3 hybrids × 2 reciprocals × 4 yield traits = 24 hybrid × reproductive trait combinations) had significant (17) or marginally significant (3) HPH (Figure 3), a pattern mimicked in the SCA estimates (Figure 1, G−J). In contrast, no reproductive trait exhibited HPH in the hybrids between C24 and Col. These hybrids had 228 fewer siliques, 11,000 fewer seeds, 1-mm shorter siliques, and over 2 fewer seeds per silique than predicted by parental GCA estimates (Figure 1, G−J).

A small number of hybrids without C24 as a parent, most notably hybrids with L*er*, exhibited heterotic traits. For example, hybrids between L*er* and Ws exceeded both parental lines in silique length, height at flowering, and number of seeds per silique (Figure 2, D–E, Figure 3). The L*er* x Ws hybrid was 43% taller than the tallest parent at flowering (Figure 2E). L*er* is homozygous for the recessive *erecta* gene that reduces plant height at flowering and silique length. All eight L*er* hybrids had significant MPH for silique length, and all but one L*er* hybrid had significant MPH for height at flowering (Figure S1). Heterosis was rare in hybrids that had neither C24 nor L*er* as a parent. Of the 72 parent-hybrid trait comparisons among Col, Cvi, and Ws, three had marginally significant HPH (Figure 3).

Reciprocal effects explained less genotypic variance than did GCA and SCA, but they were marginally significant for four traits and significant for five others: number of days to bolting, days to flowering, days to mature seed, average silique length, and average seeds per silique (Table 2). The five significant reciprocal effects were partitioned into maternal and nonmaternal effects. These were significant or marginally significant for all five traits (Table 2). For example, C24 had a positive, maternal effect on flowering time, and Ws had a marginally significant (*P* < 0.05) negative, maternal effect on flowering time (Table S1). However, the difference between C24 x Ws and Ws x C24 flowering times (*e.g.*, days to flowering was 43% later than the high parent *vs.* 15% later than the high parent, respectively) was greater than could be accounted for by maternal effects (Figure 2A, Table S1).

We also examined the effects of both low- and high-density plantings on traits. Density had a significant effect on the total number of siliques (*P* = 0.003) and a marginally significant effect on total seeds, final height, and shoot biomass (Table 2). Genotype x density interactions were not significant (Table 2).

### Contribution of *FRI* and *FLC* to hybrid traits: Introgression analyses

C24 was a parent of most hybrids within the diallel that exhibited heterotic traits, and the effect of C24 varied across traits and hybrids. C24 has a functional *FRI* allele and a weak *FLC* allele (Sanda and Amasino 1995). Col, Cvi, and Ws all have nonfunctional *FRI* alleles and strong *FLC* alleles (Lee *et al.* 1993; Koornneef *et al.* 1994; Lee *et al.* 1994; Alonso-Blanco *et al.* 1998; Michaels and Amasino 1999; Johanson *et al.* 2000; Gazzani *et al.* 2003). L*er* has a nonfunctional *FRI* allele and a weak *FLC* allele (Koornneef *et al.* 1994; Lee *et al.* 1994; Michaels and Amasino 2001). As described previously, *FRI* and *FLC* have been shown to interact epistatically within inbred genotypes to delay flowering. We used L*er* and Col ecotypes introgressed with functional and nonfunctional *FLC* and *FRI* alleles to determine if allelic variation at *FRI* and *FLC* explained the flowering time traits and other traits of C24 hybrids. To determine if *FLC* contributed to C24 hybrid trait values, we compared C24 hybrids with a functional *FLC* to hybrids without a functional *FLC* [*e.g.* (C24 x Col) +/+ hybrids compared with (C24 x Col) +/− hybrids and (C24 x L*er*) +/+ hybrids compared with (C24 x L*er*) +/− hybrids]. To evaluate the degree to which *FRI* and *FLC* alleles could explain the C24 hybrid phenotypes, we compared hybrids with functional *FRI* and *FLC* (+/+) to the introgression lines with functional *FRI* and *FLC* (+/+), and we compared hybrids with functional *FRI* only (+/−) to the inbred introgression lines with *FRI* only (+/−). If +/+ and +/− introgression lines resembled +/+ and +/− hybrid lines, respectively, then *FRI* and *FLC* fully accounted for the hybrid phenotype.

**Figure 4:**
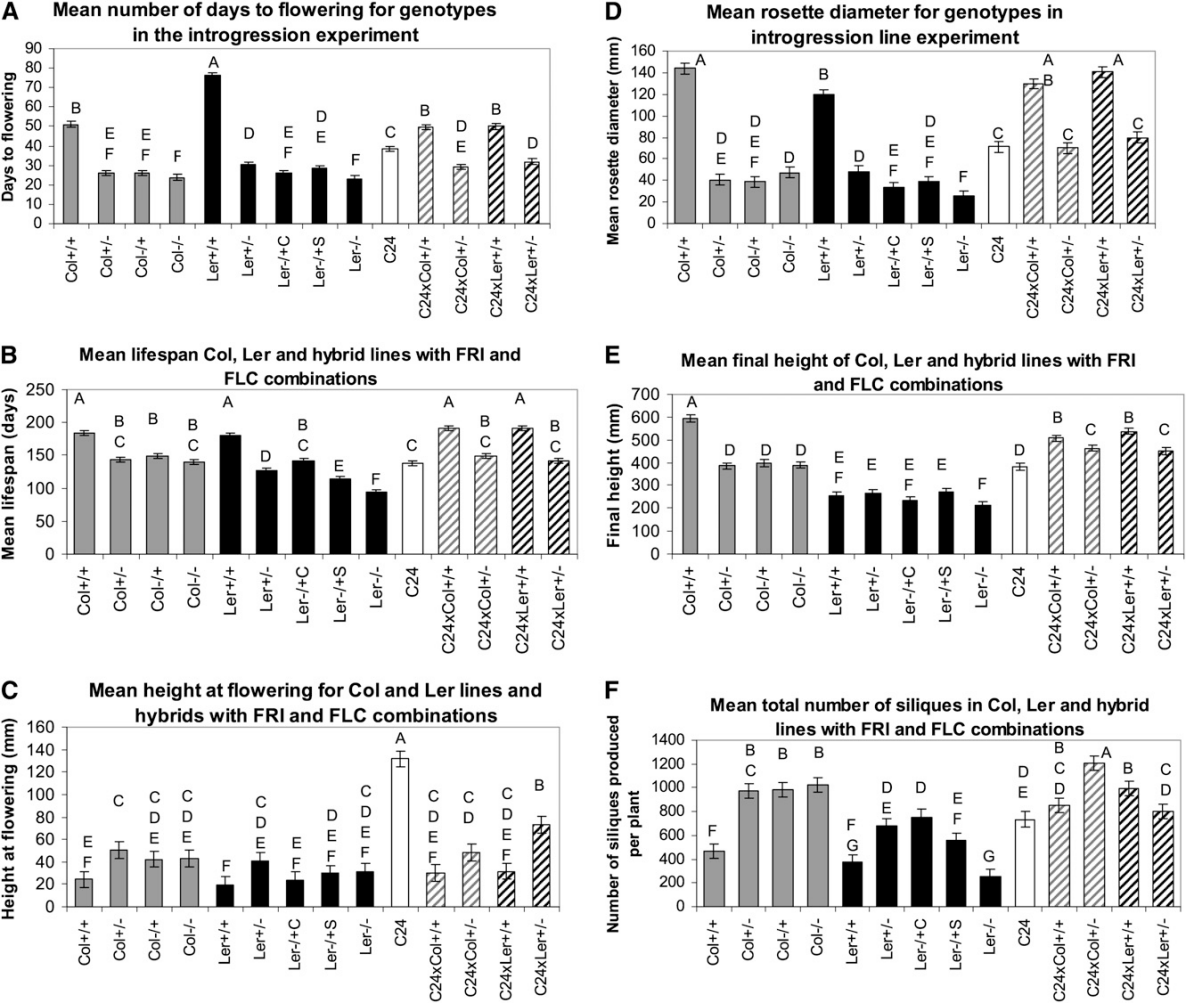
The effects of *FRI* and *FLC* on isogenic line and C24 hybrid traits. The mean trait values of fourteen Col, L*er*, C24 x Col and C24 x L*er* lines with various *FRI* and *FLC* allele combinations are plotted. (A) Mean number of days to flowering. (B) Mean height at flowering. (C) Mean rosette diameter. (D) Mean final height. (E) Mean total number of siliques. (F) Mean lifespan. Functional or strong alleles are indicated by “+”, whereas nonfunctional or weak alleles are indicated by “−”. The *FRI* allele is listed before the “/” and the *FLC* allele is listed after. The shade of the bars indicates the genotypic background: solid gray bars are Col inbred lines; black bars are L*er* inbred lines; white bars are C24 lines; striped gray and white bars are hybrids between Col and C24; and striped black and white bars are hybrids between L*er* and C24. Bars with different letters are significantly different at *P* < 0.05.

Functional alleles of *FRI* and *FLC* explained heterosis for flowering time traits in the hybrids between C24 and Col (Figure 4A, Figure S2, Figure S3). For example, Col +/+ flowered after 51 days and the (C24 x Col) +/+ hybrid flowered after 50 days. The Col +/− line flowered after 26 days and the (C24 x Col) +/− hybrid flowered after 29 days (Figure 4A). Within L*er* and C24 hybrids, *FRI* and *FLC* contributed to hybrid flowering time traits but did not fully explain them. The L*er* +/− line resembled the (C24 x L*er*) +/− hybrid for all flowering time traits (for example, 31 days to flowering *vs.* 32 days). However, the L*er* +/+ inbred line bolted, flowered, and matured later than the (C24 x L*er*) +/+ hybrid (for example, 76 days to flowering *vs.* 50 days) (Figure 4A, Figure S3). The large effects of functional *FRI* and *FLC* on flowering time traits were the result of epistasis. For example, flowering was delayed by 114% (27 days) in Col +/+ relative to Col −/− (Figure 4A), but *FRI* and *FLC* individually had no effect, as Col +/− and Col −/+ did not differ from Col −/− (Figure 4A). This finding is consistent with previous reports (Lee *et al.* 1994; Lee and Amasino 1995; Michaels and Amasino 1999). Although genotype was not a significant component of lifespan variance within the diallel, in the introgression experiment, both (C24 x Col) +/+ and (C24 x L*er*) +/+ lived significantly longer than their parents (Figure 4B). The increased hybrid lifespan of C24 x Col (+/+) could be fully ascribed to an epistatic interaction between *FRI* and *FLC* (Figure 4B).

Among the diallel genotypes, C24 hybrids often had a low height at flowering. Variation within *FRI* and *FLC* contribute to this trait in the hybrids because the (+/+) C24 hybrids with L*er* were significantly shorter at flowering than the corresponding C24 x L*er* (+/−) hybrids, and the (+/+) C24 hybrids with Col were nominally shorter than the (+/−) C24 hybrids with Col (Figure 4C). In addition, the (+/+) Col and L*er* genotypes were significantly shorter than the (+/−) introgression lines and resembled their respective (+/+) hybrids (Figure 4C).

Like flowering time heterosis, biomass trait heterosis was affected by *FRI* and *FLC*. Within hybrids and introgression lines, the effects of *FRI* and *FLC* on rosette diameter were similar to their effects on flowering time traits, except C24 (+/−) hybrids were larger than their respective (+/−) inbreds (Figure 4D). For example, the Col +/+ line had a rosette diameter that was 203% larger than the Col −/− line, and neither functional *FRI* nor *FLC* alone had a significant effect in the Col isogenic background (Figure 4D). In the Col genetic background, the effects of *FRI* and *FLC* on final plant height and stem biomass were similar to their effects on rosette diameter (Figure 4E, Figure S3). In contrast, in L*er*, both *FRI* and *FLC* had positive effects on stem biomass but no significant effect on final height (Figure 4E, Figure S3).

Finally, we found strong evidence that *FRI* and *FLC* negatively interact to reduce seed yields in the C24 and Col hybrids, largely explaining the poor performance of this hybrid in the diallel experiment. All (C24 x Col) +/− hybrids had significant HPH for reproductive traits (total siliques, total seeds, silique length, and seeds per silique), and no (C24 x Col) +/+ hybrid had HPH for these traits (Figure 4F, Figure S3). For example, the (C24 x Col) +/+ hybrid had 29% fewer siliques and 17% fewer seeds per silique than the (C24 x Col) +/− hybrid (Figure 4F, Figure S3). The poor hybrid seed production was likely the result of epistasis. The Col +/+ line averaged 55% fewer siliques, 76% fewer seeds, an 18% decrease in silique length, and a 34% decrease in the average number of seeds per silique compared to the Col −/− line, whereas Col −/−, Col −/+, and Col +/− did not significantly differ from each other for these traits (Figure 4F, Figure S3).

A strong *FLC* in the C24 x L*er* hybrid reduced the number of seeds per silique, but the total number of seed and silique length were not significantly different from the C24 x L*er* hybrid without *FLC* (Figure S3). A strong *FLC* increased the total number of siliques in the (C24 x L*er*) +/+ hybrid compared to the (C24 x L*er*) +/− hybrid (Figure 4F). Within the L*er* introgression lines, an epistatic interaction caused a significant reduction in reproductive traits because the L*er* +/+ line had lower trait values than expected given the individual effects of *FRI* and *FLC* (Figure 4F, Figure S3).

## Discussion

### The genetic components of diallel trait variation

To investigate how environment and genotype contribute to heterosis for 12 *A. thaliana* life history traits, we grew 20 hybrids from five parental *A. thaliana* genotypes in two planting densities by using a complete diallel design. Genotype and its GCA and SCA partitions explained a highly significant proportion of the variation for all but one trait, lifespan. A very high proportion of the variation for flowering time traits and a high but lower proportion of reproductive trait variation were explained by GCA and SCA. Other genetic studies have reported that flowering time trait variation has a relatively strong genetic component and reproductive traits have a relatively weak genetic component. For example, genetic variances for flowering time traits were high relative to variances for reproductive traits in *Brassica carinata* (Teklewold and Becker 2005). Variation in reproductive traits likely does have a strong genetic component. However, yield is a highly environmentally sensitive, multigenic trait (Hittalmani *et al.* 2003, Goff 2011), and yield traits thus have relatively high variation among replicates of the same genotype. Indeed, in our study, the coefficients of variation for total seeds and total siliques were greater than the coefficients of variation for flowering time traits (data not shown).

Far more than other ecotypes, C24 produced hybrids that exhibited heterotic traits. Most traits in hybrids between C24 and Cvi as well as between C24 and Ws exceeded parental levels (Figure 3). Hybrids between C24 and Col flowered late and had large biomasses, but the hybrids’ reproductive traits did not exceed parental levels (Figure 3). Hybrids between C24 and L*er* did not flower late, had moderate heterosis for biomass, and exceeded parental levels for reproductive traits (Figure 2 and Figure 3). The fact that heterosis for flowering time and yield co-occur in some hybrids but not others could explain reported differences in flowering time and yield correlations. For example, Aarssen and Clauss (1992) reported that late flowering plants with substantial vegetative growth generate large yields under favorable growth conditions. In contrast, Pigliucci and Schlichting (1995) reported that bolting time and plant size were negatively correlated to seed and fruit production.

Reciprocal effects accounted for a small component of the genetic variance compared to GCA and SCA effects and were significant for three flowering time and two yield traits. For these traits, both maternal and nonmaternal effects were significant or marginally significant. Maternal and nonmaternal effects may have an impact on reciprocal hybrids that rival the effect of the nuclear genotype (Corey *et al.* 1976; Alonso-Blanco *et al.* 1999). For example, Corey *et al.* (1976) found that maternal effects accounted for more variance for five early growth traits in a diallel analysis than did the GCA and SCA effects. Maternal effects usually have a larger effect on seed traits and traits early in a plant’s life than traits that express late in life (Kromer and Gross 1987). Although a number of reciprocal hybrids had substantially different yield and flowering time trait values, the traits we measured may explain why reciprocal effects played a relatively small role.

High density caused significant reductions in total number of siliques (Table 2), and density x genotype interactions were not significant. Previous studies in Arabidopsis have shown that high planting densities reduce plant size, accelerate flowering time, reduce leaf size, and decrease fecundity because of competition and/or red-far red signals (Aarssen and Clauss 1992; Franklin and Whitelam 2005; Keuskamp and Pierik 2010). *A. thaliana* hybrids are also more tolerant than inbreds to some stresses such as temperature (Griffing and Langridge 1963). The absence of widespread density effects and possibly the absence of density x genotype interactions are likely the result of the mild density stress.

### The effect of *FLC* and *FRI* on heterosis

Within hybrids between C24 and Col, we found that *FLC* and *FRI* fully accounted for flowering time trait heterosis and largely accounted for rosette diameter and shoot biomass heterosis. These findings are remarkable because they suggest that all genetic differences between C24 and Col outside of these two genes do not contribute to heterosis for these traits. Allelic variation in other genes may be rare because naturally occurring alleles in genes other than *FRI* and *FLC* may have pleiotropic detrimental effects, as suggested by both Koornneef *et al.* (1994) and Johanson *et al.* (2000). Alternatively, genes that do differ between Col and C24 could have subtle effects in long day, unvernalized growth conditions. For example, in two studies several small-effect QTL affecting flowering time were detected only after *FLC* was down-regulated through vernalization (Alonso-Blanco *et al.* 1998; Werner *et al.* 2005; Strange *et al.* 2011).

*FLC* also contributed to the low yield of the C24 and Col hybrid and likely to its short height at flowering. The effect *FLC* on traits other than flowering time traits may be caused by *FLC*’s direct role in the development of these traits, because of its effect on flowering time that in turn affects these traits, or both. The negative effect of *FLC* on height at flowering appears to be structurally related to flowering time. Koornneef *et al.* (1994) described a dominant late flowering mutant *florens* (*F*)—one of the latest flowering Arabidopsis genotypes—as having a poorly elongated main stem, and Pigliucci and Schlichting (1995) also found that late flowering plants consistently have a low height at flowering. We postulate that the role of *FLC* on reproductive development is attributable in part to its participation in this process. Loss-of-function mutations in *FCA*, which negatively regulates *FLC*, affect silique production in addition to the vegetative to floral transition (Tienderen *et al.* 1996; Macknight *et al.* 1997). *FLC* is expressed in multiple tissues during Arabidopsis development, including the root, aerial tissue, rosette leaves, and floral buds (Sheldon *et al.* 2000). In addition, *FLC* has a large number of promoter binding sites in genes that are involved in a number of developmental pathways, including reproductive development (Deng *et al.* 2011). A mutant flowering time gene in tomato, *SINGLE FLOWER TRUSS* (*SFT*), an ortholog of *A. thaliana FLOWERING LOCUS T* (*FT*) (Krieger *et al.* 2010), causes heterosis of inflorescence number and flowers per inflorescence.

*FLC* had a smaller effect on traits in C24 and L*er* hybrids than in C24 and Col hybrids. There were no heterotic traits for which both L*er* (+/+) mimicked (C24 x L*er*) +/+ and L*er* (+/−) mimicked (C24 x L*er*) +/−. *FLC* likely had a relatively weak role in C24 x L*er* heterosis because of the strong phenotypic effect of *erecta*, which was complemented in the hybrid. Indeed, hybrids of a tester line with *angustifolia* (*an*) and *erecta* (*er*) mutants resulted in heterosis for numerous traits, including length of the main stem, total number of siliques, and both fresh and dry weight (Rédei 1962).

For most traits, we found that *FRI* and *FLC* interact epistatically to positively or negatively influence phenotypic values. Manipulating such epistatic interactions may be a general mechanism to improve traits in breeding populations. The yield increase in tomatoes caused by heterozygosity at *SFT* is due to suppression of growth termination imposed by the *SELF PRUNING* (*SP*) gene (Krieger *et al.* 2010). The high performance of elite European rapeseed (*Brassica napus* L.) and Brussels sprouts (*Brassica oleracea* var. *gemmifera*) is caused in part by beneficial epistatic interactions (Werner *et al.* 1989; Engqvist and Becker 1991), and Lamkey *et al.* (1995) proposed that elite maize genotypes have favorable epistatic interactions between linked genes. By extension, selection of lines with favorable GCA, or additive, trait estimates for further development may not be a productive method to enhance hybrid traits. In this study, C24 had significant, positive GCA estimates, but GCA alone poorly predicted hybrid traits because of epistasis between *FRI* and *FLC*. As the genetic basis for hybrid trait variation is studied in greater depth, we predict epistasis will have a major role in its explanation.

## Supplementary Material

Supporting Information
