# Supplementary material for: An Evaluation of Arabidopsis thaliana Hybrid Traits and Their Genetic Control
Source: G3 (Bethesda). 2011 Dec 1;1(7):571–9. doi: 10.1534/g3.111.001156 (PMC3276180; doi:10.1534/g3.111.001156)
Supplement: Supporting Information [file supp_1_7_571__index.html]

Supporting Information 

# An Evaluation of *Arabidopsis thaliana* Hybrid Traits and Their Genetic Control

## Supporting Information for Moore and Lukens, 2011

**Files in this Data Supplement:**

- Supporting Information - Figures S1-S3, File S1, and Tables S1-S6 (PDF, 1.8 MB)
- Figure S1 - The percent MPH and HPH for all hybrids within the diallel for all 12 traits (PDF, 376 KB)
- Figure S2 - Mean trait values for all genotypes in the diallel and introgression experiments (PDF, 416 KB)
- Figure S3 - Means of the fourteen introgression experiment genotypes for the 6 traits not included in main text (PDF, 380 KB)
- File S1 - Supporting Materials and Methods (PDF, 372 KB)
- Table S1 - Reciprocal and non-maternal estimates for hybrids and maternal and SCA estimates of selfed parental lines from the diallel analysis (PDF, 316 KB)
- Table S2 - Raw data from diallel analysis (PDF, 308 KB)
- Table S3 - Raw data for the number of seeds per silique averaged for the diallel experiment (PDF, 336 KB)
- Table S4 - Raw data for the average silique length in the diallel experiment (PDF, 364 KB)
- Table S5 - Raw data from the *FRI* and *FLC* experiment (PDF, 340 KB)
- Table S6 - Raw data for the number of seeds per silique and silique length measurements in the *FRI* and *FLC* experiments (PDF, 340 KB)
