# Supplementary material for: An Evaluation of Arabidopsis thaliana Hybrid Traits and Their Genetic Control
Source: G3 (Bethesda). 2011 Dec 1;1(7):571–9. doi: 10.1534/g3.111.001156 (PMC3276180; doi:10.1534/g3.111.001156)
Supplement: Supporting Information [file supp_1.7.571_001156SI.pdf]

File S1

Supporting Materials and Methods

Plant growth and trait measurements

All seeds were sterilized and incubated at 4º for four days then placed on germination media. Seedlings were germinated under continuous light at room temperature. After two weeks the seedlings were moved to 3.5” x 3.5” pots with LA-4 Sunshine Mix (Sun Gro) with one pellet of 14-14-14 fertilizer and placed in a growth chamber (Conviron). Light intensity from fluorescent bulbs was measured at the shelf level. Plants were watered with deionized tap water approximately every three days or as needed.

Days to bolting was the time between germination and when a visible bud was formed. Days to flowering was the time between germination and when a flower fully opened. Days to mature seed was time until a silique was fully browned and opened to release mature seeds. Height at flowering was measured from the base of the stem to the top of the plant. For silique number, all mature siliques were collected and counted for each plant throughout development. (In the diallel, 87,306 siliques were counted, and in the introgression experiment 61,818 siliques were counted.) The silique length was averaged from 25 siliques per plant in the diallel and five siliques per plant in the introgression experiment. The number of seeds within a silique was averaged from 20 siliques per plant in the diallel and five siliques per plant in the introgression experiment. (In the diallel, 97,410 seeds were counted, and in the introgression experiment, 20,710 seeds were counted.) The final plant height was measured from the base of the stem to the top of the tallest branch at death. The lifespan was considered the time from sowing on germination media until death. Shoot biomass was determined by drying the stem (with no rosette leaves) at 80º for 48 hours then weighing it. The total number of seeds was estimated by multiplying the total number of siliques and the average number of seeds per silique for each plant. Plants were checked daily.

Seed source

The five *A. thaliana* parental lines, Columbia (Col), Wassilewskija (Ws), Landsberg *erecta* (L*er*), Cape Verde Islands (Cvi), and C24, were obtained from Dr. Stephen Chatfield and originally ordered from Lehle Seeds (catalogue numbers WT-2, WT-8A, WT-4, WT-18, and WT-23, respectively). Manual self pollinations of parent lines were made to control for differences in seeds produced by selfing versus manual crossing (Meyer *et al*. 2004).

We obtained Col and Ler genotypes into which a number of FRI and FLC alleles had been introgressed. Non-functional *FLC* alleles were produced with fast neutron radiation mutagenesis (*flc*-3) (Col -/-), while the functional *FRI* allele was introgressed into wild-type Col -/+ and Col -/- from the Sf2 accession (producing Col +/+ and Col +/-, respectively) (Lee and Amasino 1995; Michaels and Amasino 1999). In the L*er* background (wild-type L*er* -/-), functional alleles of *FRI* and *FLC* were introgressed from the Sf2 accession (producing L*er* -/+, L*er* +/-, and L*er* +/+) (Lee *et al*. 1994). An additional line was used with a functional *FLC* allele from Col introgressed into L*er* (L*er* -/+ *FLC*-Col) (Koornneef *et al*. 1994). Wild-type seeds were obtained from Dr. Stephen Chatfield, all other seeds were obtained from Dr. Richard Amasino, except for L*er* -/+ *FLC*-Col, which was ordered from The Arabidopsis Biological Research Center (ABRC) (Alonso et al. 2003). The introgressed segment is referred to using the name of introgressed gene of interest (i.e. *FRI* or *FLC*) (Lee *et al*. 1993; Koornneef *et al*. 1994; Lee *et al*. 1994; Michaels and Amasino 1999; Michaels and Amasino 2001).

C24 hybrids were generated by crossing the following lines: C24 +/- with Col -/+; C24 +/- with L*er* -/+ (*FLC*-Col); C24 +/- with Col -/-; and C24 +/- with L*er* -/-, respectively

Statistical analyses

DIALLEL-SAS05, the program that executed Griffing's diallel analysis, required balanced data to compute diallel statistics. Therefore, the trait values for the three plants with incorrect genotypes (as determined by CAPS marker analysis) were substituted with pseudoreplicates - estimated trait values calculated from other plants of the correct genotype.

We used the following model for the split plot analysis of diallel data

$$Y_{ijk} = \mu + \alpha_i + \eta_{k(i)} + \beta_j + \alpha\beta_{ij} + \varepsilon_{k(ij)}$$

where α_i is the whole plot factor (density); $\eta_{k(i)}$ is the replication nested within the density; β_j is the subplot factor (genotype); $\alpha\beta_{ij}$ is the interaction between genotype and density; and $\varepsilon_{k(ij)}$ is the residual error.

Among the introgression lines and their hybrids, we used the following model:

$$Y_{ij} = \mu + \alpha_i + \beta_j + \varepsilon_{ij}$$

where α_i is the genotype (treatment) effect, β_j is the block effect, and ε_{ij} is the residual error.

Among inbreds, five sets of contrasts were performed to determine the effects of *FRI* and *FLC*. (1) To evaluate the effect of *FRI* in a background with a non-functional *FLC* allele, the +/- line was compared to the -/- line. (2) The effect of *FRI* in a background with a functional *FLC* was determined by contrasting the -/+ and +/+ lines. (3) The effect of *FLC* with a non-functional *FRI* was determined by contrasting the -/- and -/+ lines. (4) The effect of *FLC* when a functional *FRI* is present was determined by contrasting +/- and +/+ lines. Finally, (5) the effect of both genes together was determined by comparing the -/- and +/+ varieties.

References

Alonso, J. et al., 2003. Genome-wide insertional mutagenesis of *Arabidopsis thaliana*. Science 301: 653-657.

Koornneef, M., H. B. Vries, C. Hanhart, W. Soppe and T. Peeters, 1994 The phenotype of some late-flowering mutants is enhanced by a locus on chromosome 5 that is not effective in the Landsberg *erecta* wild-type. Plant J. **6:** 911-919.

Lee, I., A. Bleecker and R. Amasino, 1993 Analysis of naturally occurring late flowering in *Arabidopsis thaliana*. Mol. Gen. Genet. **237:** 171-176.

Lee, I., S. D. Michaels, A. S. Masshardt and R. M. Amasino, 1994 The late-flowering phenotype of *FRIGIDA* and mutations in *LUMINIDEPENDENS* is suppressed in the Landsberg *erecta* strain of Arabidopsis. Plant J. **6:** 903-909.

Lee, I., and R. M. Amasino, 1995 Effect of vernalization, photoperiod, and light quality on the flowering phenotype of Arabidopsis plants containing the FRIGIDA gene. Plant Physiol. **108:** 157-162.

Meyer, R. C., O. Torjek, M. Becher and T. Altmann, 2004 Heterosis of biomass production in Arabidopsis. Establishment during early development. Plant Physiol. **134:** 1813-1823.

Michaels, S. D., and R. M. Amasino, 1999 FLOWERING LOCUS C encodes a novel MADS domain protein that acts as a repressor of flowering. Plant Cell **11:** 949-956.

Michaels, S. D., and R. M. Amasino, 2001 Loss of FLOWERING LOCUS C activity eliminates the late-flowering phenotype of FRIGIDA and autonomous pathway mutations but not responsiveness to vernalization. Plant Cell **13**: 935-942.

A) Days to Bolting

MPH	C24	Col	Cvi	*Ler*	Ws
C24	-3.77	119.46	59.42	12.57	82.42
Col	103.24	16.46	-6.67	-2.56	-1.94
Cvi	59.42	-7.78	-2.97	-13.48	-11.86
Ler	18.03	-2.56	-13.48	0.00	-0.65
Ws	40.66	10.97	-14.12	-0.65	0.00

HPH	C24	Col	Cvi	*Ler*	Ws
C24		91.51	55.66	-2.83	56.60
Col	77.36		-16.83	-3.80	-3.80
Cvi	55.66	-17.82		-23.76	-22.77
Ler	1.89	-3.80	-23.76		-1.30
Ws	20.75	8.86	-24.75	-1.30	

B) Days to Flowering

MPH	C24	Col	Cvi	*Ler*	Ws
C24	-7.20	91.19	47.11	8.04	63.47
Col	77.97	8.82	-7.76	-4.48	-2.04
Cvi	48.76	-4.11	-0.85	-13.89	-4.27
Ler	13.39	-6.47	-8.33	-3.03	-0.52
Ws	31.51	10.20	-14.69	0.52	-3.19

HPH	C24	Col	Cvi	*Ler*	Ws
C24		73.60	42.40	-3.20	43.20
Col	61.60		-13.68	-5.88	-5.88
Cvi	44.00	-10.26		-20.51	-13.68
Ler	1.60	-7.84	-15.38		-3.03
Ws	15.20	5.88	-23.08	-2.02	

C) Days to Mature Seed

MPH	C24	Col	Cvi	*Ler*	Ws
C24	-4.37	64.22	33.90	8.24	45.07
Col	54.25	5.06	-5.78	-3.49	-2.80
Cvi	37.85	-3.95	1.17	-7.93	-5.88
Ler	8.82	0.95	-5.49	-1.27	-2.91
Ws	21.19	4.52	-10.84	-0.32	-2.63

HPH	C24	Col	Cvi	*Ler*	Ws
C24		53.01	29.51	0.55	32.79
Col	43.72		-9.36	-3.80	-4.64
Cvi	33.33	-7.60		-11.70	-11.11
Ler	1.09	0.63	-9.36		-4.46
Ws	10.93	2.53	-15.79	-1.91	

D) Rosette Diameter

MPH	C24	Col	Cvi	*Ler*	Ws
C24	-6.54	229.35	197.98	71.06	215.17
Col	234.55	38.60	-8.96	18.02	13.29
Cvi	202.92	2.49	-20.78	9.90	-22.17
Ler	93.28	8.72	4.46	-8.09	23.56
Ws	147.81	1.16	2.46	14.37	6.86

HPH	C24	Col	Cvi	*Ler*	Ws
C24		196.26	187.01	54.67	186.45
Col	200.93		-20.78	17.34	12.00
Cvi	191.77	-10.82		-3.90	-31.60
Ler	74.77	8.09	-8.66		22.86
Ws	125.23	0.00	-9.96	13.71	

E) Shoot Biomass

MPH	C24	Col	Cvi	*Ler*	Ws
C24	6.80	224.56	267.02	214.91	363.99
Col	204.29	7.80	-17.54	-33.79	-4.24
Cvi	312.73	29.23	-0.87	-33.78	-30.33
Ler	136.65	21.68	13.27	-6.58	51.45
Ws	423.02	39.45	9.00	-6.36	37.00

HPH	C24	Col	Cvi	*Ler*	Ws
C24		211.21	247.45	135.09	203.71
Col	191.77		-24.96	-51.91	-38.53
Cvi	290.73	17.59		-48.62	-53.11
Ler	76.66	-11.63	-12.11		23.20
Ws	242.35	-10.50	-26.64	-23.82	

F) Final Height

MPH	C24	Col	Cvi	*Ler*	Ws
C24	3.59	38.24	47.02	59.10	79.17
Col	36.91	4.13	2.62	-13.54	1.90
Cvi	45.05	3.11	-5.59	15.34	5.33
Ler	57.51	-2.37	28.25	-12.93	22.61
Ws	68.99	20.73	24.98	32.39	21.26

HPH	C24	Col	Cvi	*Ler*	Ws
C24		32.68	40.11	30.31	56.70
Col	31.40		-5.94	-31.40	-13.95
Cvi	38.24	-5.48		-1.71	-3.79
Ler	29.00	-22.54	9.29		13.52
Ws	47.79	1.95	14.16	22.57	

G) Total Number of Siliques

MPH	C24	Col	Cvi	*Ler*	Ws
C24	3.64	-5.59	85.49	78.86	73.99
Col	2.14	12.36	0.89	-29.56	10.57
Cvi	84.52	19.98	-12.68	-12.69	-4.79
Ler	62.69	1.56	27.06	-41.56	32.59
Ws	134.25	32.28	59.80	-21.27	15.03

HPH	C24	Col	Cvi	*Ler*	Ws
C24		-10.30	43.63	29.93	32.31
Col	-2.95		-24.64	-50.42	-18.79
Cvi	42.88	-10.38		-20.31	-7.19
Ler	18.19	-28.52	15.97		23.93
Ws	78.14	-2.85	55.76	-26.42	

H) Total Number of Seeds

MPH	C24	Col	Cvi	*Ler*	Ws
C24	-2.00	1.39	109.19	139.85	121.93
Col	13.18	8.57	-1.78	-12.61	7.46
Cvi	114.69	20.89	-17.76	-25.17	-15.11
Ler	132.09	13.12	51.65	-45.62	50.15
Ws	194.66	44.92	73.14	-0.43	14.88

HPH	C24	Col	Cvi	*Ler*	Ws
C24		-8.93	49.62	78.03	66.75
Col	1.66		-34.05	-39.45	-24.76
Cvi	53.56	-18.83		-29.35	-21.21
Ler	72.28	-21.61	43.19		47.44
Ws	121.39	1.47	60.70	-2.23	

I) Average Silique Length

MPH	C24	Col	Cvi	*Ler*	Ws
C24	0.16	-2.23	17.03	32.36	14.66
Col	2.64	-7.55	9.99	24.90	7.89
Cvi	19.29	10.72	-0.95	18.38	2.33
Ler	35.17	18.55	34.48	-4.51	31.68
Ws	28.32	19.82	4.66	27.17	5.33

HPH	C24	Col	Cvi	*Ler*	Ws
C24		-2.87	15.35	11.88	3.11
Col	1.97		7.72	6.14	-2.41
Cvi	17.58	8.43		-1.11	-9.15
Ler	14.25	0.75	12.33		22.77
Ws	15.40	8.38	-7.08	18.56	

J) Average Number of Seeds per Silique

MPH	C24	Col	Cvi	*Ler*	Ws
C24	-2.26	8.32	16.09	37.13	30.45
Col	11.89	-1.54	2.75	31.32	3.59
Cvi	20.71	6.28	-5.56	-5.11	-9.77
Ler	46.20	18.06	19.22	-5.48	19.07
Ws	29.14	13.74	5.24	31.69	0.53

HPH	C24	Col	Cvi	*Ler*	Ws
C24		1.72	5.30	36.40	27.87
Col	5.07		-11.90	23.94	-4.52
Cvi	9.49	-8.87		-14.34	-16.64
Ler	45.42	11.42	7.62		16.11
Ws	26.59	4.84	-2.78	28.41	

K) Height at Flowering

MPH	C24	Col	Cvi	Ler	Ws
C24	-12.79	-61.18	-31.91	16.05	-18.69
Col	-45.65	4.74	-8.62	65.97	-0.90
Cvi	2.38	21.03	0.98	43.84	-2.05
Ler	77.78	75.76	46.92	43.75	93.82
Ws	-17.65	43.86	-22.25	91.27	7.22

HPH	C24	Col	Cvi	Ler	Ws
C24		-68.03	-33.33	-15.86	-20.46
Col	-55.24		-25.98	40.71	-16.93
Cvi	0.25	-1.96		2.94	-6.13
Ler	28.90	49.01	5.15		42.51
Ws	-19.44	20.59	-25.49	40.64	

LPH	C24	Col	Cvi	Ler	Ws
C24		-50.59	-30.43	86.93	-16.84
Col	-30.83		19.37	102.27	22.79
Cvi	4.60	58.10		138.64	2.41
Ler	186.36	114.20	143.75		202.84
Ws	-15.78	78.26	-18.72	198.86	

L) Lifespan

MPH	C24	Col	Cvi	Ler	Ws
C24	-0.20	-11.73	1.90	0.76	8.77
Col	-10.01	-2.60	2.31	11.34	8.56
Cvi	-0.76	-8.42	-7.92	-4.70	-6.06
Ler	9.41	7.56	8.05	2.42	27.53
Ws	6.26	10.86	4.24	0.81	15.66

HPH	C24	Col	Cvi	Ler	Ws
C24		-13.94	-1.10	-8.81	1.96
Col	-12.27		1.84	-1.49	-0.62
Cvi	-3.68	-8.84		-16.02	-14.36
Ler	-0.98	-4.83	-4.79		22.82
Ws	-0.39	1.49	-4.97	-2.91	

FIGURE S1 The percent MPH and HPH for all hybrids within the diallel for all 12 traits Within each matrix, the value in the cell indicates the percent difference from: the midparent value for mid-parent heterosis (MPH); the high-parent value for high-parent heterosis (HPH); and the low-parent value for low-parent heterosis (LPH) . LPH is only shown for height at flowering because no other trait had significant low-parent heterosis. The color of each cell indicates the significance level of the difference. The maternal genotype is on the vertical axis and the paternal genotype is on the horizontal axis. All 12 traits are shown for comparison purposes, though four are also included in main text.
A - days to bolting, - **B** days to flowering, **C** - days to mature seed, **D** - rosette diameter, **E** - shoot biomass, **F** - final height, **G** - total number of siliques, **H** - total number of seeds, **I** - silique length, **J** - average number of seeds per silique, **K** - height at flowering, **L** – lifespan.

Hybrid Larger	Hybrid Smaller
P < 0.0001	P < 0.0001
0.0001 < P < 0.001	0.0001 < P < 0.001
0.001 < P < 0.01	0.001 < P < 0.01
0.01 < P < 0.05	0.01 < P < 0.05
0.05 < P < 0.1	0.05 < P < 0.1

A

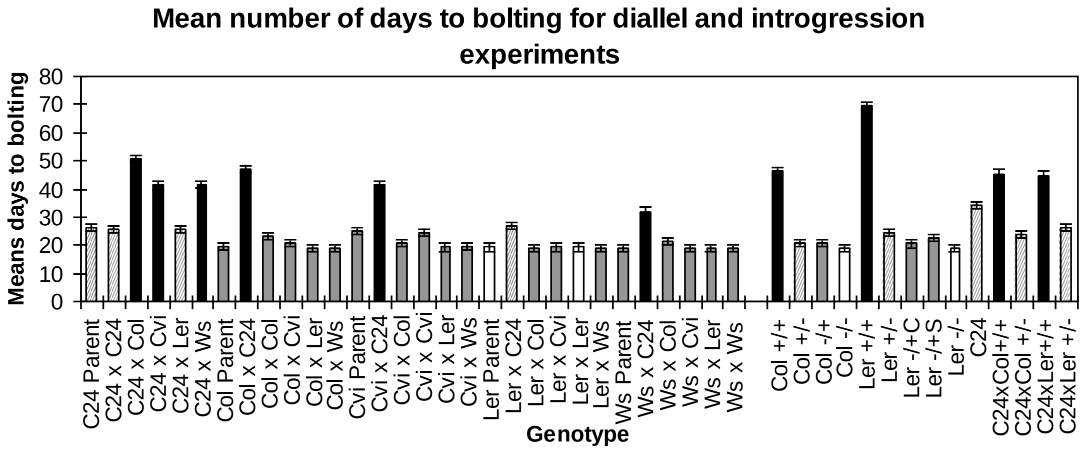
Mean number of days to bolting for diallel and introgression experiments
Means days to bolting
0
10
20
30
40
50
60
70
80
C24 Parent
C24 x C24
C24 x Col
C24 x Cvi
C24 x Ler
C24 x Ws
Col Parent
Col x C24
Col x Col
Col x Cvi
Col x Ler
Col x Ws
Cvi Parent
Cvi x C24
Cvi x Col
Cvi x Cvi
Cvi x Ler
Cvi x Ws
Ler Parent
Ler x C24
Ler x Col
Ler x Cvi
Ler x Ler
Ler x Ws
Ws Parent
Ws x C24
Ws x Col
Ws x Cvi
Ws x Ler
Ws x Ws
Col +/+
Col +/-
Col -/+
Col -/-
Ler +/+
Ler +/-
Ler -/+C
Ler -/+S
Ler -/-
C24
C24xCol+/+
C24xCol +/-
C24xLer+/+
C24xLer +/-
Genotype

B

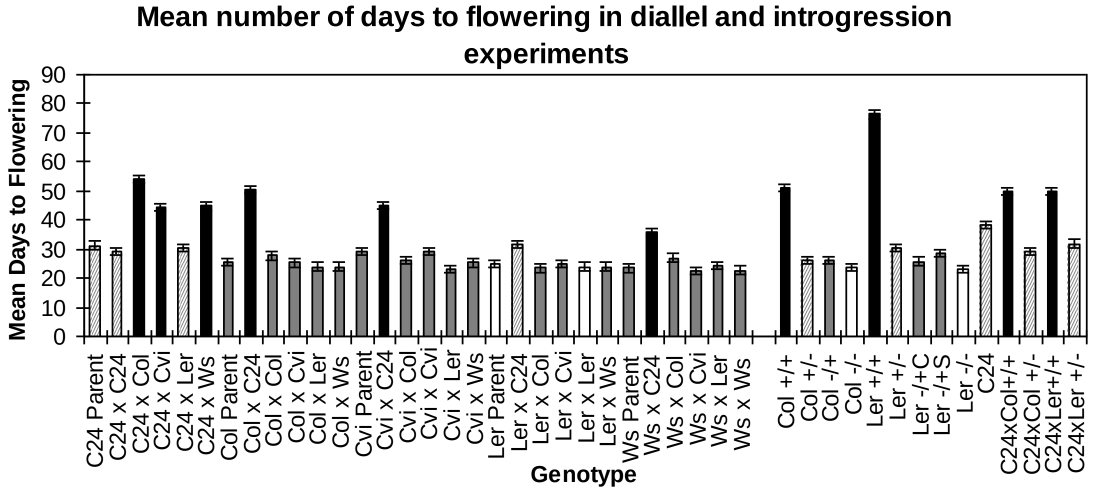
Mean number of days to flowering in diallel and introgression experiments
Mean Days to Flowering
0
10
20
30
40
50
60
70
80
90
C24 Parent
C24 x C24
C24 x Col
C24 x Cvi
C24 x Ler
C24 x Ws
Col Parent
Col x C24
Col x Col
Col x Cvi
Col x Ler
Col x Ws
Cvi Parent
Cvi x C24
Cvi x Col
Cvi x Cvi
Cvi x Ler
Cvi x Ws
Ler Parent
Ler x C24
Ler x Col
Ler x Cvi
Ler x Ler
Ler x Ws
Ws Parent
Ws x C24
Ws x Col
Ws x Cvi
Ws x Ler
Ws x Ws
Col +/+
Col +/-
Col -/+
Col -/-
Ler +/+
Ler +/-
Ler -/+C
Ler -/+S
Ler -/-
C24
C24xCol+/+
C24xCol +/-
C24xLer+/+
C24xLer +/-
Genotype

C

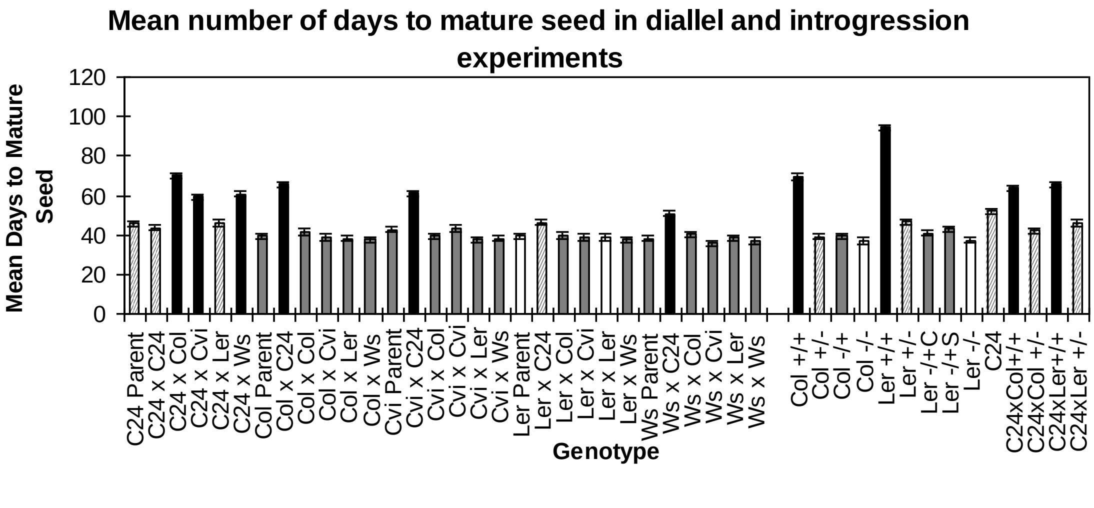
Mean number of days to mature seed in diallel and introgression experiments
Mean Days to Mature Seed
0
20
40
60
80
100
120
C24 Parent
C24 x C24
C24 x Col
C24 x Cvi
C24 x Ler
C24 x Ws
Col Parent
Col x C24
Col x Col
Col x Cvi
Col x Ler
Col x Ws
Cvi Parent
Cvi x C24
Cvi x Col
Cvi x Cvi
Cvi x Ler
Cvi x Ws
Ler Parent
Ler x C24
Ler x Col
Ler x Cvi
Ler x Ler
Ler x Ws
Ws Parent
Ws x C24
Ws x Col
Ws x Cvi
Ws x Ler
Ws x Ws
Col +/+
Col +/-
Col -/+
Col -/-
Ler +/+
Ler +/-
Ler -/+C
Ler -/+S
Ler -/-
C24
C24xCol+/+
C24xCol +/-
C24xLer+/+
C24xLer +/-
Genotype

p[

D

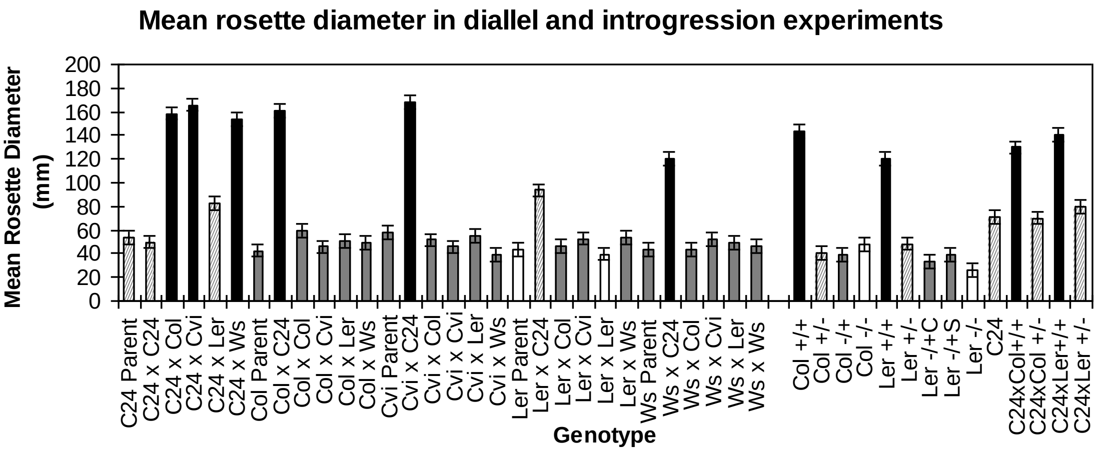
Mean rosette diameter in diallel and introgression experiments
Mean Rosette Diameter (mm)
0
20
40
60
80
100
120
140
160
180
200
C24 Parent
C24 x C24
C24 x Col
C24 x Cvi
C24 x Ler
C24 x Ws
Col Parent
Col x C24
Col x Col
Col x Cvi
Col x Ler
Col x Ws
Cvi Parent
Cvi x C24
Cvi x Col
Cvi x Cvi
Cvi x Ler
Cvi x Ws
Ler Parent
Ler x C24
Ler x Col
Ler x Cvi
Ler x Ler
Ler x Ws
Ws Parent
Ws x C24
Ws x Col
Ws x Cvi
Ws x Ler
Ws x Ws
Col +/+
Col +/-
Col -/+
Col -/-
Ler +/+
Ler +/-
Ler -/+C
Ler -/+S
Ler -/-
C24
C24xCol+/+
C24xCol +/-
C24xLer+/+
C24xLer +/-
Genotype

E

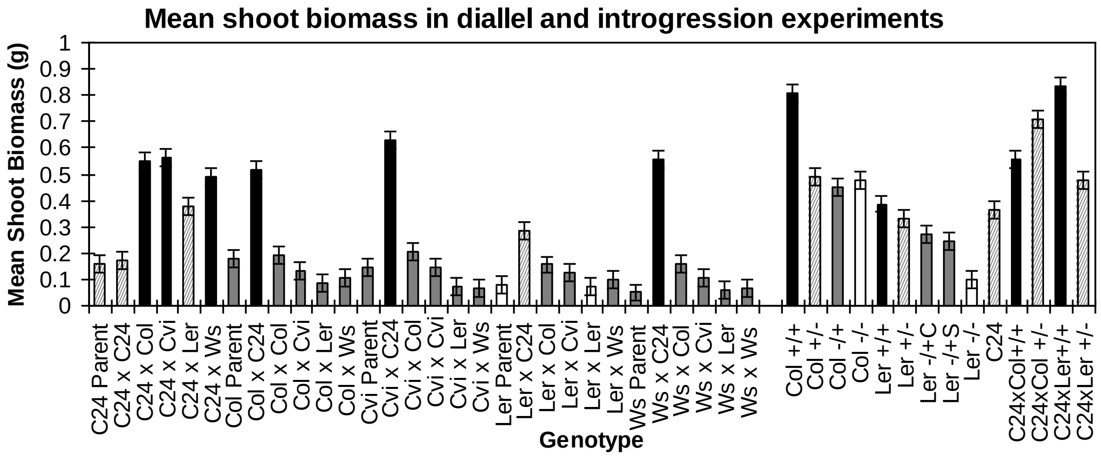
Mean shoot biomass in diallel and introgression experiments
Mean Shoot Biomass (g)
0
0.1
0.2
0.3
0.4
0.5
0.6
0.7
0.8
0.9
1
C24 Parent
C24 x C24
C24 x Col
C24 x Cvi
C24 x Ler
C24 x Ws
Col Parent
Col x C24
Col x Col
Col x Cvi
Col x Ler
Col x Ws
Cvi Parent
Cvi x C24
Cvi x Col
Cvi x Cvi
Cvi x Ler
Cvi x Ws
Ler Parent
Ler x C24
Ler x Col
Ler x Cvi
Ler x Ler
Ler x Ws
Ws Parent
Ws x C24
Ws x Col
Ws x Cvi
Ws x Ler
Ws x Ws
Col +/+
Col +/-
Col -/+
Col -/-
Ler +/+
Ler +/-
Ler -/+C
Ler -/+S
Ler -/-
C24
C24xCol+/+
C24xCol +/-
C24xLer+/+
C24xLer +/-
Genotype

F

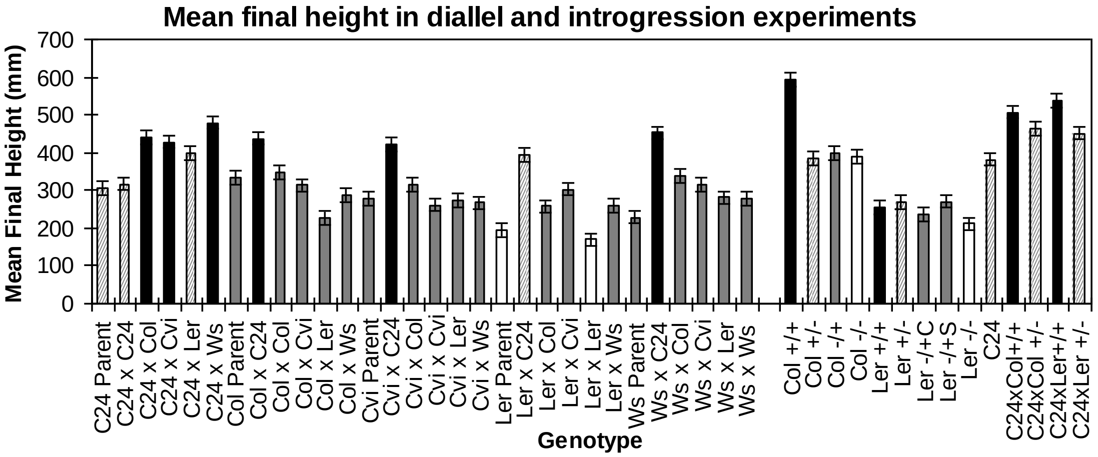
Mean final height in diallel and introgression experiments
Mean Final Height (mm)
0
100
200
300
400
500
600
700
C24 Parent
C24 x C24
C24 x Col
C24 x Cvi
C24 x Ler
C24 x Ws
Col Parent
Col x C24
Col x Col
Col x Cvi
Col x Ler
Col x Ws
Cvi Parent
Cvi x C24
Cvi x Col
Cvi x Cvi
Cvi x Ler
Cvi x Ws
Ler Parent
Ler x C24
Ler x Col
Ler x Cvi
Ler x Ler
Ler x Ws
Ws Parent
Ws x C24
Ws x Col
Ws x Cvi
Ws x Ler
Ws x Ws
Col +/+
Col +/-
Col -/+
Col -/-
Ler +/+
Ler +/-
Ler -/+C
Ler -/+S
Ler -/-
C24
C24xCol+/+
C24xCol +/-
C24xLer+/+
C24xLer +/-
Genotype

G

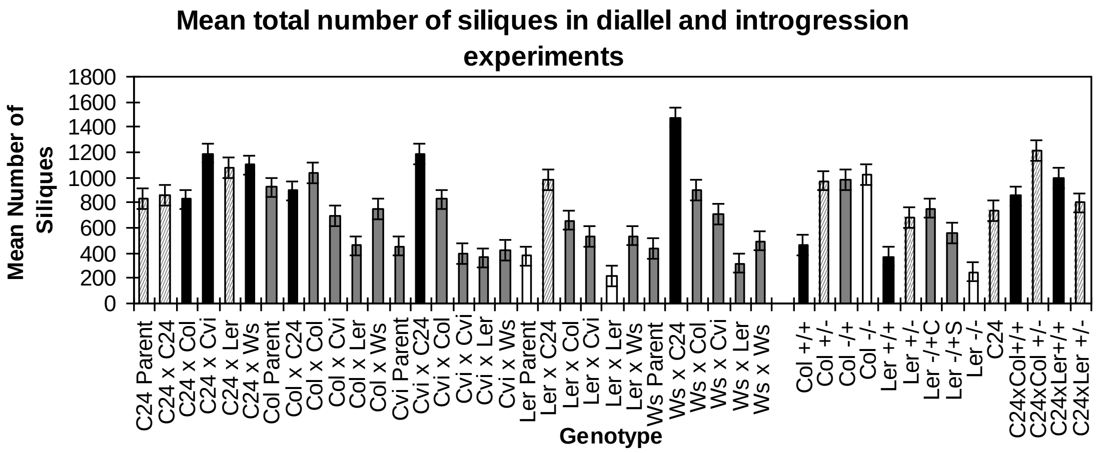
Mean total number of siliques in diallel and introgression experiments
Mean Number of Siliques
1800
1600
1400
1200
1000
800
600
400
200
0
C24 Parent
C24 x C24
C24 x Col
C24 x Cvi
C24 x Ler
C24 x Ws
Col Parent
Col x C24
Col x Col
Col x Cvi
Col x Ler
Col x Ws
Cvi Parent
Cvi x C24
Cvi x Col
Cvi x Cvi
Cvi x Ler
Cvi x Ws
Ler Parent
Ler x C24
Ler x Col
Ler x Cvi
Ler x Ler
Ler x Ws
Ws Parent
Ws x C24
Ws x Col
Ws x Cvi
Ws x Ler
Ws x Ws
Col +/+
Col +/-
Col -/+
Col -/-
Ler +/+
Ler +/-
Ler -/+C
Ler -/+S
Ler -/-
C24
C24xCol+/+
C24xCol +/-
C24xLer+/+
C24xLer +/-
Genotype

H

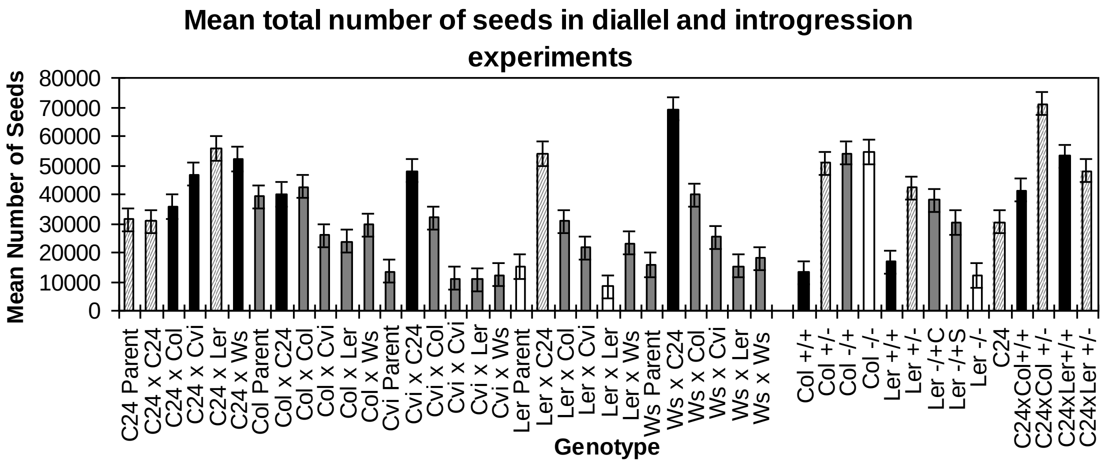
Mean total number of seeds in diallel and introgression experiments
Mean Number of Seeds
80000
70000
60000
50000
40000
30000
20000
10000
0
C24 Parent
C24 x C24
C24 x Col
C24 x Cvi
C24 x Ler
C24 x Ws
Col Parent
Col x C24
Col x Col
Col x Cvi
Col x Ler
Col x Ws
Cvi Parent
Cvi x C24
Cvi x Col
Cvi x Cvi
Cvi x Ler
Cvi x Ws
Ler Parent
Ler x C24
Ler x Col
Ler x Cvi
Ler x Ler
Ler x Ws
Ws Parent
Ws x C24
Ws x Col
Ws x Cvi
Ws x Ler
Ws x Ws
Col +/+
Col +/-
Col -/+
Col -/-
Ler +/+
Ler +/-
Ler -/+C
Ler -/+S
Ler -/-
C24
C24xCol+/+
C24xCol +/-
C24xLer+/+
C24xLer +/-
Genotype

I

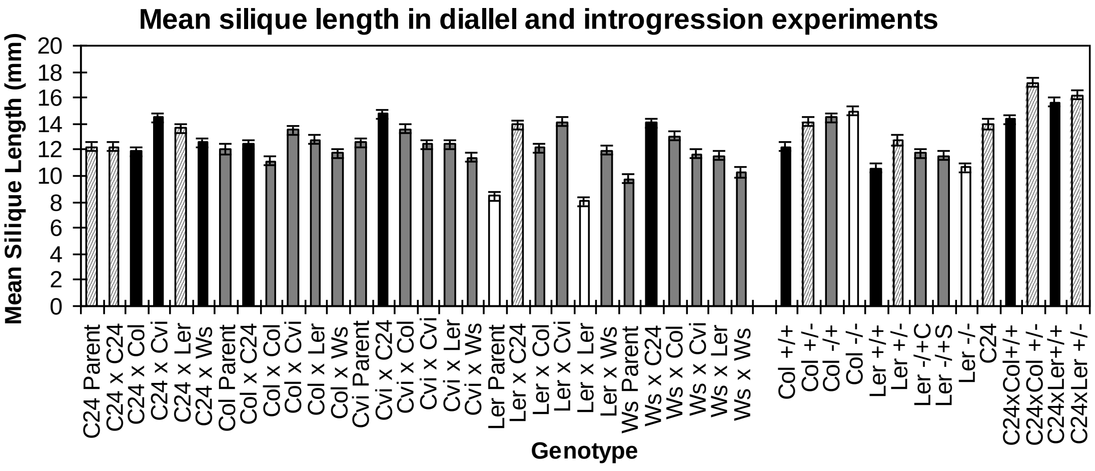
Mean silique length in diallel and introgression experiments
Mean Silique Length (mm)
20
18
16
14
12
10
8
6
4
2
0
C24 Parent
C24 x C24
C24 x Col
C24 x Cvi
C24 x Ler
C24 x Ws
Col Parent
Col x C24
Col x Col
Col x Cvi
Col x Ler
Col x Ws
Cvi Parent
Cvi x C24
Cvi x Col
Cvi x Cvi
Cvi x Ler
Cvi x Ws
Ler Parent
Ler x C24
Ler x Col
Ler x Cvi
Ler x Ler
Ler x Ws
Ws Parent
Ws x C24
Ws x Col
Ws x Cvi
Ws x Ler
Ws x Ws
Col +/+
Col +/-
Col -/+
Col -/-
Ler +/+
Ler +/-
Ler -/+C
Ler -/+S
Ler -/-
C24
C24xCol+/+
C24xCol +/-
C24xLer+/+
C24xLer +/-
Genotype

J

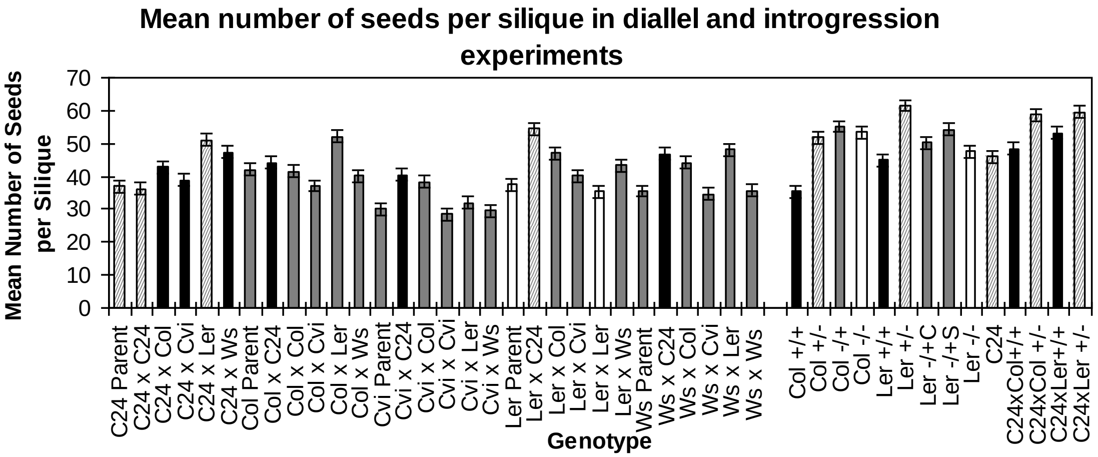
Mean number of seeds per silique in diallel and introgression experiments
Mean Number of Seeds per Silique
Genotype
C24 Parent
C24 x C24
C24 x Col
C24 x Cvi
C24 x Ler
C24 x Ws
Col Parent
Col x C24
Col x Col
Col x Cvi
Col x Ler
Col x Ws
Cvi Parent
Cvi x C24
Cvi x Col
Cvi x Cvi
Cvi x Ler
Cvi x Ws
Ler Parent
Ler x C24
Ler x Col
Ler x Cvi
Ler x Ler
Ler x Ws
Ws Parent
Ws x C24
Ws x Col
Ws x Cvi
Ws x Ler
Ws x Ws
Col +/+
Col +/-
Col -/+
Col -/-
Ler +/+
Ler +/-
Ler -/+C
Ler -/+S
Ler -/-
C24
C24xCol+/+
C24xCol +/-
C24xLer+/+
C24xLer +/-

K

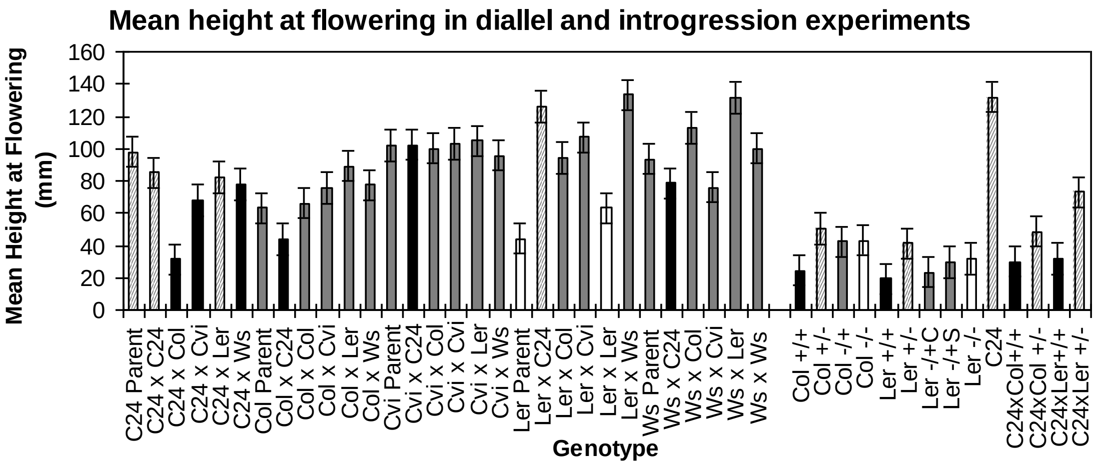
Mean height at flowering in diallel and introgression experiments
Mean Height at Flowering (mm)
Genotype

L

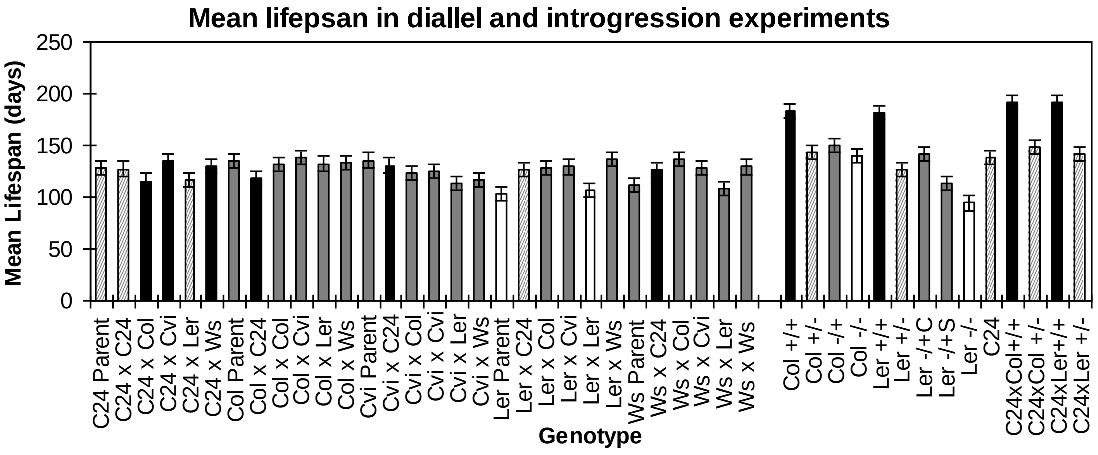
Mean lifepsan in diallel and introgression experiments
Mean Lifespan (days)
Genotype

Figure S2 Mean trait values for all genotypes in the diallel and introgression experiments. The means of all 12 traits (A-L) in the diallel and *FRI* and *FLC* introgression line experiments were plotted on the same chart to compare means across experiments. The color of the bar corresponds to the *FRI* and *FLC* status of the genotype: black have functional *FRI* and strong *FLC*; striped have functional *FRI* only; grey have functional *FLC* only; and white have non-functional alleles at both *FRI* and *FLC*. The means for the diallel span the density treatments since density had little effect on traits. The bars are the standard error of the mean estimates. **A -** days to bolting, - **B** days to flowering, **C** - days to mature seed, **D** - rosette diameter, **E** - shoot biomass, **F** - final height, **G** - total number of siliques, **H** - total number of seeds, **I** - silique length, **J** - average number of seeds per silique, **K** - height at flowering, **L** – lifespan.

A

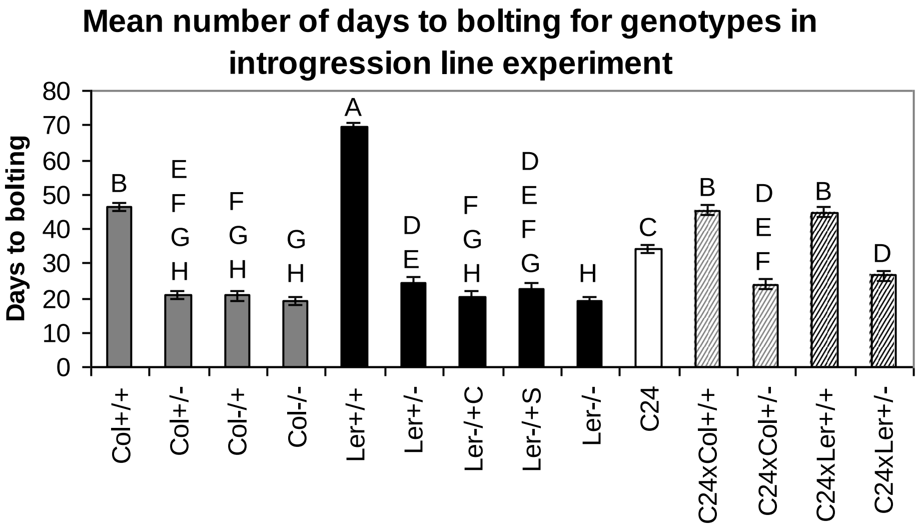
Mean number of days to bolting for genotypes in introgression line experiment
Days to bolting
80
70
60
50
40
30
20
10
0
B
E F G H
F G H
G H
A
D E
F G H
D E F G
H
C
B
D E F
B
D
Col+/+
Col+/-
Col-/+
Col-/-
Ler+/+
Ler+/-
Ler-/+C
Ler-/+S
Ler-/-
C24
C24xCol+/+
C24xCol+/-
C24xLer+/+
C24xLer+/-

B

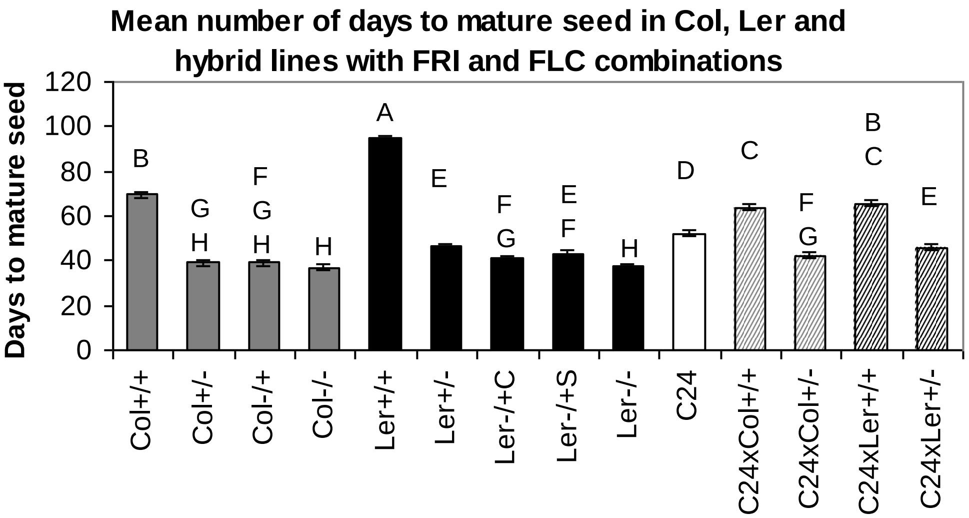
Mean number of days to mature seed in Col, Ler and hybrid lines with FRI and FLC combinations
Days to mature seed
120
100
80
60
40
20
0
B
G H
F G H
H
A
E
F G
E F
H
D
C
F G
B C
E
Col+/+
Col+/-
Col-/+
Col-/-
Ler+/+
Ler+/-
Ler-/+C
Ler-/+S
Ler-/-
C24
C24xCol+/+
C24xCol+/-
C24xLer+/+
C24xLer+/-

C

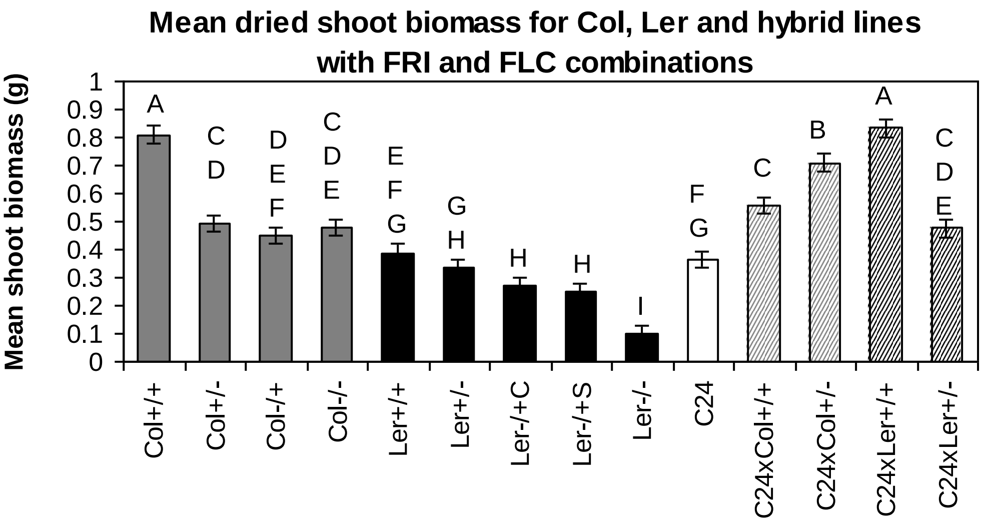

Mean dried shoot biomass for Col, Ler and hybrid lines with FRI and FLC combinations
Mean shoot biomass (g)
1
0.9
0.8
0.7
0.6
0.5
0.4
0.3
0.2
0.1
0
A
C D
D E F
C D E
E F G
G H
H
H
I
F G
C
B
A
C D E
Col+/+
Col+/-
Col-/+
Col-/-
Ler+/+
Ler+/-
Ler-/+C
Ler-/+S
Ler-/-
C24
C24xCol+/+
C24xCol+/-
C24xLer+/+
C24xLer+/-

D

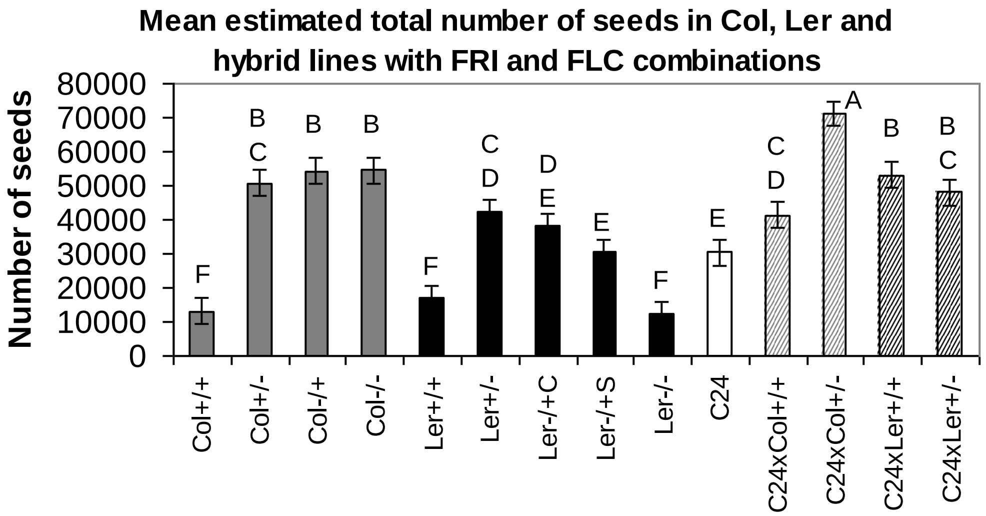

Mean estimated total number of seeds in Col, Ler and hybrid lines with FRI and FLC combinations
Number of seeds
80000
70000
60000
50000
40000
30000
20000
10000
0
F
B C
B
B
F
C D
D E
E
F
E
C D
A
B
B C
Col+/+
Col+/-
Col-/+
Col-/-
Ler+/+
Ler+/-
Ler-/+C
Ler-/+S
Ler-/-
C24
C24xCol+/+
C24xCol+/-
C24xLer+/+
C24xLer+/-

E

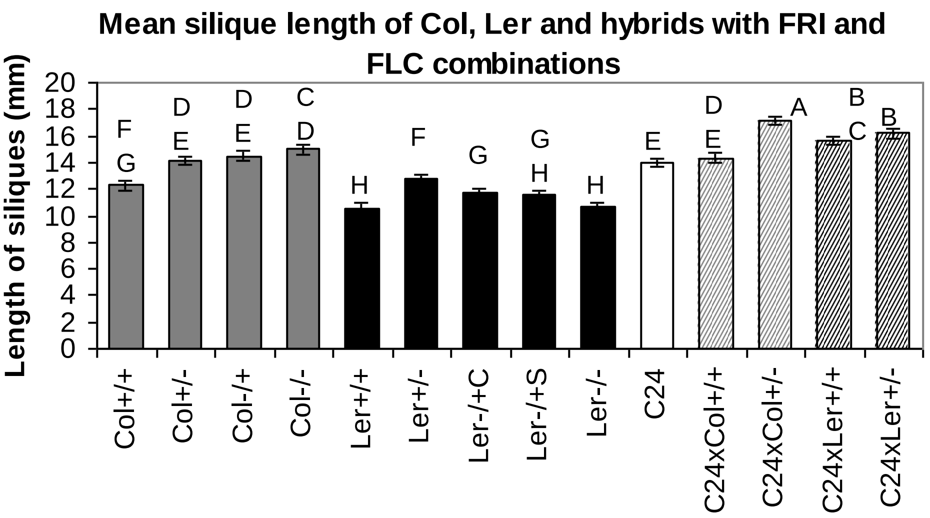

F

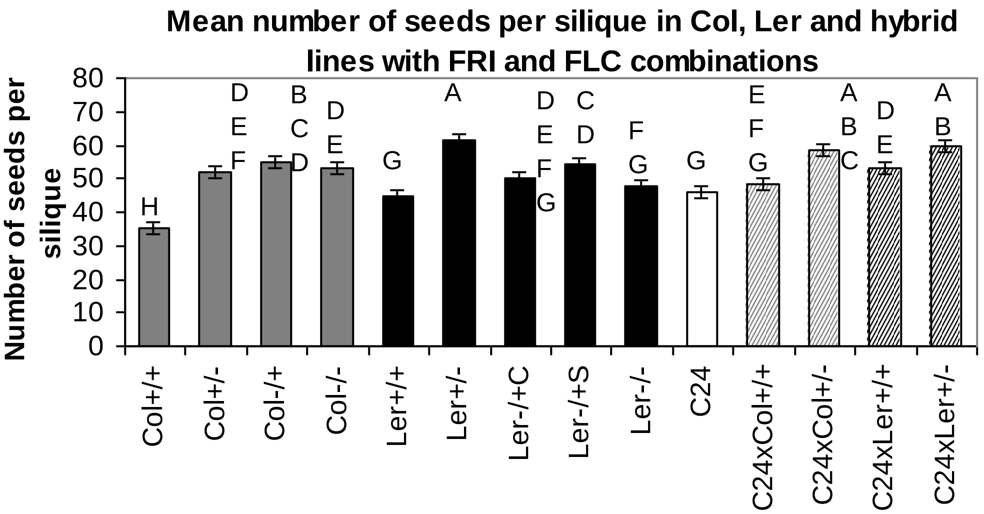

Figure S3 Means of the fourteen introgression experiment genotypes for the 6 traits not included in main text. The mean values of Col, L*er*, C24 x Col and C24 x L*er* lines with various *FRI* and *FLC* allele combinations. Functional or strong alleles are indicated by "+", while non-functional or weak alleles are indicated by "-". The status of the *FRI* allele is listed before the "/" and the status of *FLC* listed after. The color of the bars indicates the genotypic background: solid grey bars are Col inbred lines, black bars are L*er* inbred lines, white bars are C24 lines, striped grey and white bars are hybrids between Col and C24, and striped black and white bars are hybrids between L*er* and C24. Bars with different letters are significantly different at P < 0.05.
A: Mean number of days to bolting.
B: Mean number of days to mature seed.
C: Mean shoot biomass.
D: Mean estimated total number of seeds.
E: Mean silique length for all 14 lines.
F: Mean number of seeds per silique.

Table S1 Reciprocal and non-maternal estimates for hybrids and maternal and SCA estimates of selfed parental lines from the diallel analysis

Trait	Diallel component	Hybrid Genotypes										Parental Genotypes				
		C24 x Col	C24 x Cvi	C24 x *Ler*	C24 x Ws	Col x Cvi	Col x *Ler*	Col x Ws	Cvi x *Ler*	Cvi x Ws	*Ler* x Ws	C24	Col	Cvi	*Ler*	Ws
Days to bolting	REC	1.88*	0.00	-0.63	4.75**	0.13	0.00	-1.25	0.00	0.25	0.00	-	-	-	-	-
	MAT	-	-	-	-	-	-	-	-	-	-	1.20**	-0.60	0.03	0.13	-0.75*
	NMAT	0.08	-1.18	-1.70**	2.80**	0.75	0.73	-1.40*	0.10	-0.53	-0.88	-	-	-	-	-
	Selfed parent SCA	-	-	-	-	-	-	-	-	-	-	-20.04**	-6.24**	0.41	4.06**	-22.55**
Days to flowering	REC	1.88*	-0.25	-0.75	4.38**	-0.50	0.25	-1.50	-0.75	1.38	-0.13	-	-	-	-	-
	MAT	-	-	-	-	-	-	-	-	-	-	1.05**	-0.73*	0.28	0.23	-0.83*
	NMAT	0.10	-1.03	-1.58*	2.50**	0.50	1.20	-1.60*	-0.80	0.28	-1.18	-	-	-	-	-
	Selfed parent SCA	-	-	-	-	-	-	-	-	-	-	-19.62**	-6.32**	0.43	3.63**	-23.40**
Days to mature seed	REC	2.13*	-0.88	-0.13	5.00**	-0.38	-0.88	-1.42	-0.50	1.00	-0.50	-	-	-	-	-
	MAT	-	-	-	-	-	-	-	-	-	-	1.23**	-0.96*	0.35	0.20	-0.82*
	NMAT	-0.06	-1.75*	-1.15	2.96**	0.93	0.28	-1.28	-0.65	-0.17	-1.52*	-	-	-	-	-
	Selfed parent SCA	-	-	-	-	-	-	-	-	-	-	-20.87**	-6.66**	0.98	3.58**	-23.88**
Rosette diameter	REC	-1.25	-1.38	-5.38	16.38**	-2.88	2.00	2.63	1.38	-6.25	2.00	-	-	-	-	-
	MAT	-	-	-	-	-	-	-	-	-	-	1.68	0.60	-0.13	0.80	-2.95
	NMAT	-2.33	-3.18	-6.25	11.75**	-3.60	2.20	-0.93	2.30	-9.08**	-1.75	-	-	-	-	-
	Selfed parent SCA	-	-	-	-	-	-	-	-	-	-	-113.72**	-14.97**	-21.77**	3.78	-153.90**
Shoot biomass	REC	0.02	-0.04	0.05	-0.03	-0.04	-0.04	-0.02	-0.03	-0.02	0.02	-	-	-	-	-
	MAT	-	-	-	-	-	-	-	-	-	-	0.00	-0.02	0.01	0.01	0.01
	NMAT	-0.01	-0.03	0.05*	-0.02	-0.01	-0.01	0.01	-0.03	-0.01	0.02	-	-	-	-	-
	Selfed parent SCA	-	-	-	-	-	-	-	-	-	-	-0.45**	-0.03	-0.06	0.03	-0.55**
Final height	REC	2.13	2.88	2.00	13.63	-0.75	-14.75	-26.46	-15.25	-24.88	-10.38	-	-	-	-	-
	MAT	-	-	-	-	-	-	-	-	-	-	4.13	-8.82	-8.45	3.53	9.62
	NMAT	-10.82	-9.70	1.40	19.12	-0.38	-2.41	-8.03	-3.28	-6.81	-4.28	-	-	-	-	-
	Selfed parent SCA	-	-	-	-	-	-	-	-	-	-	-170.43**	11.86	-40.03*	-46.73*	-284.37**
Total siliques	REC	-33.88	3.13	48.75	-190.25*	-65.75	-101.00	-73.50	-82.63	-143.38	108.88	-	-	-	-	-
	MAT	-	-	-	-	-	-	-	-	-	-	-34.45	-41.28	-32.68	48.75	59.65
	NMAT	-40.70	4.90	131.95	-96.15	-57.15	-10.98	27.43	-1.20	-51.05	119.78	-	-	-	-	-
	Selfed parent SCA	-	-	-	-	-	-	-	-	-	-	-476.90**	146.10	-191.60	-98.05	-809.25**
Total seeds	REC§	-2.08	-0.62	0.90	-8.55*	-2.97	-3.50	-5.15	-5.50	-6.44	3.91	-	-	-	-	-
	MAT§	-	-	-	-	-	-	-	-	-	-	-2.07	-1.91	-1.67	2.40	3.25*
	NMAT§	-1.92	-0.21	5.34	-3.23	-2.74	0.81	0.01	-1.43	-1.53	4.75	-	-	-	-	-
	Selfed parent SCA§	-	-	-	-	-	-	-	-	-	-	-29.75**	7.13	-5.73	-9.92*	-48.68**
Silique length	REC	-0.30	-0.14	-0.15	-0.75**	-0.05	0.33	-0.65**	-0.85**	-0.13	0.21	-	-	-	-	-
	MAT	-	-	-	-	-	-	-	-	-	-	-0.27**	-0.02	-0.16	0.17	0.27**

	NMAT	-0.04	-0.03	0.30	-0.22	-0.19	0.51**	-0.37	-0.51**	0.29	0.30	-	-	-	-	-
	Selfed parent SCA	-	-	-	-	-	-	-	-	-	-	-1.74**	-0.87**	-1.25**	-3.20**	-8.02**
Seeds per silique	REC	-0.71	-0.78	-1.69	0.24	-0.64	2.64*	-1.97	-4.11**	-2.46	-2.30	-	-	-	-	-
	MAT	-	-	-	-	-	-	-	-	-	-	-0.59	0.15	-1.03*	0.17	1.30*
	NMAT	0.03	-1.22	-0.93	2.12*	-1.82	2.66**	-0.82	-2.90**	-0.13	-1.17	-	-	-	-	-
	Selfed parent SCA	-	-	-	-	-	-	-	-	-	-	-10.20**	-1.27	0.11	-11.12**	-26.56**
Height at flowering	REC	-6.25	-17.13*	-21.88**	-0.50	-12.25	-2.63	-17.54*	-1.13	9.88	0.88	-	-	-	-	-
	MAT	-	-	-	-	-	-	-	-	-	-	-9.15**	-5.23	7.63*	5.30	1.46
	NMAT	-2.33	-0.35	-7.43	10.11	0.61	7.91	-10.85	-3.45	3.71	-2.97	-	-	-	-	-
	Selfed parent SCA	-	-	-	-	-	-	-	-	-	-	17.65*	-4.69	4.35	-47.25**	-37.98
Lifespan	REC	-1.13	1.75	-5.00	1.50	7.25	2.25	-1.42	-7.63	-6.38	14.38*	-	-	-	-	-
	MAT	-	-	-	-	-	-	-	-	-	-	-0.58	1.84	-4.60	4.95*	-1.62
	NMAT	1.29	-2.28	0.53	0.46	0.81	5.36	-4.88	1.93	-3.39	7.81	-	-	-	-	-
	Selfed parent SCA	-	-	-	-	-	-	-	-	-	-	2.31	-3.13	-2.44	-9.24	-13.03

The REC and NMAT effects are given for the hybrid genotypes and the MAT and the SCA are given for the parental genotypes.

§ Value x 10^3

*** P < 0.05**

**** P < 0.01**

Table S2 Raw data from diallel analysis

	Rep	Density	Block	(DAS) Bolting	(mm) Rosette Diameter	(DAS) Flowering	(mm) Flowering Height	(DAS) Mature Pod	Average Silique Length	Average # Seeds per Pod	(mm) Final Height	Total Siliques	(days) Lifespan	(g) Stem Dry Weight	Estimated Total Seeds
COL_P	1	1	1	19	51	23	41	37	12.08	48	352	1100	132	0.206	52800
COLxCOL	1	1	1	35	92	39	95	53	10.76	36.3	372	1294	146	0.211	46972.2
COLxLER	1	1	1	19	51	23	57	37	13.32	51.65	239	623	128	0.11	32177.95
COLxWS	1	1	1	19	48	24	112	38	10.88	41.2	304	939	140	0.104	38686.8
COLxCVI	1	1	1	23	42	25	79	38	14	37.65	288	898	132	0.122	33809.7
COLxC24	1	1	1	46	181	51	40	67	12.24	41.5	455	886	98	0.654	36769
LER_P	1	1	1	19	48	25	39	39	9.44	44.7	228	597	135	0.153	26685.9
LERxCOL	1	1	1	19	39	25	67	46	12.48	45.5	391	898	104	0.255	40859
LERxLER	1	1	1	19	36	23	48	37	8.8	39.25	171	320	115	0.084	12560
LERxWS	1	1	1	19	57	23	128	37	11.44	44.55	280	575	145	0.108	25616.25
LERxCVI	1	1	1	19	51	24	88	38	14.84	42.15	352	748	132	0.107	31528.2
LERxC24	1	1	1	27	95	32	152	46	13.52	50.75	480	1032	93	0.37	52374
WS_P	1	1	1	19	47	23	103	37	10.36	37.15	271	817	149	0.053	30351.55
WSxCOL	1	1	1	24	36	28	101	40	12.72	39.1	357	857	126	0.175	33508.7
WSxLER	1	1	1	19	58	23	111	37	12.32	48.2	332	495	137	0.12	23859
WSxWS	1	1	1	19	46	23	84	37	10.92	37.95	303	782	141	0.119	29676.9
WSxCVI	1	1	1	19	55	22	77	35	12.2	36.2	300	1170	153	0.149	42354
WSxC24	1	1	1	35	138	39	69	54	13.72	46.9	464	1557	126	0.815	73023.3
CVI_P	1	1	1	27	67	30	81	44	13.6	30.8	260	561	140	0.132	17278.8
CVIxCOL	1	1	1	19	45	25	94	38	13.12	35.25	329	901	131	0.2	31760.25
CVIxLER	1	1	1	19	54	22	111	36	11.92	33.9	308	343	132	0.093	11627.7
CVIxWS	1	1	1	19	30	26	93	38	11.12	27.45	231	469	149	0.031	12874.05
CVIxCVI	1	1	1	23	44	28	109	44	12.08	24.85	257	562	133	0.185	13965.7
CVIxC24	1	1	1	43	179	46	80	61	15.48	42.1	408	1301	140	0.707	54772.1
C24_P	1	1	1	28	53	32	85	46	12.44	39.15	305	1197	132	0.237	46862.55
C24xCOL	1	1	1	51	149	55	35	69	11.36	40.8	443	958	119	0.533	39086.4
C24xLER	1	1	1	22	65	27	90	42	14.52	49.7	383	897	130	0.419	44580.9
C24xWS	1	1	1	38	154	41	71	57	12.96	46.9	466	1251	132	0.564	58671.9
C24xCVI	1	1	1	39	174	43	92	55	15.44	41.1	430	1583	139	0.601	65061.3
C24xC24	1	1	1	28	52	32	111	46	12.8	36.8	351	1106	133	0.246	40700.8
COL_P	1	2	2	21	35	28	82	40	11.44	39.1	302	568	131	0.062	22208.8
COLxCOL	1	2	2	19	51	24	53	37	11	43.3	297	791	128	0.108	34250.3
COLxLER	1	2	2	19	57	24	131	37	13.08	56.25	196	383	140	0.061	21543.75
COLxWS	1	2	2	19	49	24	77.66667	37.667	11.76	40.2333	286.3333	749	133.67	0.108333	29553.4
COLxCVI	1	2	2	19	43	24	47	37	13.88	37.5	284	480	142	0.115	18000
COLxC24	1	2	2	46	122	51	41	66	13.52	47.05	403	750	94	0.291	35287.5
LER_P	1	2	2	19	43	23	46	37	8.48	34.75	205	168	97	0.055	5838
LERxCOL	1	2	2	19	49	24	91	37	12.76	52.35	162	491	133	0.101	25703.85
LERxLER	1	2	2	20	46	28	116	44	7.32	33.35	80	58	94	0.052	1934.3
LERxWS	1	2	2	19	53	25	154	38	12.32	42.5	316	416	142	0.119	17680
LERxCVI	1	2	2	20	45	25	99	38	13.8	39.1	247	137	130	0.053	5356.7
LERxC24	1	2	2	27	87	31	127	46	13.44	53.45	251	627	156	0.161	33513.15
WS_P	1	2	2	19	40	22	89	36	8.72	35.65	259	128	66	.	4563.2

WSxCOL	1	2	2	21	45	27	111	40	13.32	47.9	358	795	137	0.104	38080.5
WSxLER	1	2	2	19	50	24	129	37	11.4	51.45	268	339	106	0.031	17441.55
WSxWS	1	2	2	19	44	23	116	37	9.56	33.7	265	338	138	0.042	11390.6
WSxCVI	1	2	2	19	52	22.5	76	36	11.68	34.55	316.5	709.5	129	0.106	25273.05
WSxC24	1	2	2	34	128	38	86	53	13.8	48.3	451	1393	142	0.495	67281.9
CVI_P	1	2	2	24	48	29	113	42	11.2	28.2	266	184	146	0.029	5188.8
CVIxCOL	1	2	2	20	51	26	106	40	13.24	38.2	270	408	133	0.078	15585.6
CVIxLER	1	2	2	19	52	23	110	37	12.36	37.95	301	167	105	0.031	6337.65
CVIxWS	1	2	2	19	46	25	85	38	13.32	35.2	298	436	84	0.063	15347.2
CVIxCVI	1	2	2	23	42	28	128	41	12.52	30.5	253	185	105	0.091	5642.5
CVIxC24	1	2	2	38	139	41	83	58	14.88	37.85	356	1107	131	0.543	41899.95
C24_P	1	2	2	24	47	29	107	42	11.6	32.45	300	504	139	0.031	16354.8
C24xCOL	1	2	2	46	158	50	30	67	12.64	47.7	476	1001	132	0.595	47747.7
C24xLER	1	2	2	27	97	32	110	47	14.08	57.7	391	1170	99	0.451	67509
C24xWS	1	2	2	40	137	43	78	58	12.84	48.45	488	1076	126	0.446	52132.2
C24xCVI	1	2	2	48	189	51	40	67	14.44	41.3	416	888	131	0.576	36674.4
C24xC24	1	2	2	24	54	28	71	43	12.48	36.8	288	670	132	0.107	24656
COL_P	2	1	3	20	46	25	65	40	12.6	40.2	408	1199	128	0.303	48199.8
COLxCOL	2	1	3	19	44	23	55	37	11	40.6	339	950	130	0.159	38570
COLxLER	2	1	3	19	45	24	81	39	12.52	52.7	267	379	134	0.071	19973.3
COLxWS	2	1	3	19	47	24	49	37	12.96	37.2	291	1050	134	0.178	39060
COLxCVI	2	1	3	21	47	26	73	39	13.32	37.5	386	934	125	0.196	35025
COLxC24	2	1	3	44	178	46	58	60	12.12	48.15	465	1178	126	0.657	56720.7
LER_P	2	1	3	20	42	26	53	41	9.2	40.4	153	575	111	0.086	23230
LERxCOL	2	1	3	19	52	23	135	38	12.6	45.75	306	926	130	0.206	42364.5
LERxLER	2	1	3	19	40	22	46	36	9	41.2	241	342	101	0.107	14090.4
LERxWS	2	1	3	19	49	24	117	37	12.32	41.9	231	753	132	0.115	31550.7
LERxCVI	2	1	3	19	68	23	80	39	14.2	42.1	328	832	129	0.213	35027.2
LERxC24	2	1	3	27	104	32	124	46	15	58.6	439	1462	125	0.455	85673.2
WS_P	2	1	3	19	45	24	104	39	10.6	36.35	227	623	133	0.087	22646.05
WSxCOL	2	1	3	19	46	25	100	39	13.28	46.6	296	1306	134	0.236	60859.6
WSxLER	2	1	3	19	52	23	136	39	10.84	45.1	245	259	71	0.051	11680.9
WSxWS	2	1	3	19	46	22	105	36	10.36	36.4	252	567	140	0.087	20638.8
WSxCVI	2	1	3	19	49	23	75	37	11.16	32.9	333	249	105	0.063	8192.1
WSxC24	2	1	3	29	114	33	102	47	14.64	47.7	445	1507	111	0.482	71883.9
CVI_P	2	1	3	23	48	28	111	42	12.28	25.75	314	710	130	0.25	18282.5
CVIxCOL	2	1	3	22	65	27	104	40	14.52	40.45	339	1202	124	0.347	48620.9
CVIxLER	2	1	3	19	62	23	90	37	13.08	35	239	351	77	0.083	12285
CVIxWS	2	1	3	19	51	23	115	37	10.4	26.7	308	640	135	0.144	17088
CVIxCVI	2	1	3	26	52	30	83	45	12.64	29.3	277	629	106	0.243	18429.7
CVIxC24	2	1	3	39	179	44	157	60	13.96	40.25	484	1449	132	0.845	58322.25
C24_P	2	1	3	30	71	36	127	52	12.4	38.25	331	1153	106	0.279	44102.25
C24xCOL	2	1	3	53	160	56	30	71	12.08	43.6	429	737	106	0.585	32133.2
C24xLER	2	1	3	24	59	28	49	45	13.92	54.75	403	1284	138	0.317	70299
C24xWS	2	1	3	39	156	42	85	58	12.88	49.75	518	1178	131	0.5	58605.5
C24xCVI	2	1	3	39	180	42	65	58	15.04	40.2	431	1303	136	0.721	52380.6
C24xC24	2	1	3	26	51	28	80	43	11.88	33	339	1210	111	0.251	39930

COL_P	2	2	4	19	39	26	65	41	12.08	41.25	269	822	147	0.134	33907.5
COLxCOL	2	2	4	19	50	25	62	39	11.8	45.75	378	1110	120	0.282	50782.5
COLxLER	2	2	4	19	50	25	87	39	12.24	48.3	211	444	128	0.097	21445.2
COLxWS	2	2	4	19	52	24	72	38	11.44	42.3	264	258	127	0.043	10913.4
COLxCVI	2	2	4	21	51	26	103	41	12.96	35.85	294	468	154	0.096	16777.8
COLxC24	2	2	4	52	163	54	36	70	11.92	40.4	426	766	154	0.455	30946.4
LER_P	2	2	4	19	40	25	38	40	6.6	29.75	195	164	71	0.025	4879
LERxCOL	2	2	4	19	47	22	84	38	10.72	44.2	172	322	145	0.061	14232.4
LERxLER	2	2	4	19	37	23	43	38	7.08	27.6	188	159	114	0.055	4388.4
LERxWS	2	2	4	19	56	24	134	38	11.8	44.75	214	400	130	0.051	17900
LERxCVI	2	2	4	19	47	27	162	40	13.64	37.65	285	396	126	0.135	14909.4
LERxC24	2	2	4	27	88	32	101	47	13.84	54.75	409	804	132	0.157	44019
WS_P	2	2	4	19	43	25	78	40	9.32	33	160	162	99	0.03	5346
WSxCOL	2	2	4	22	48	28	139	43	12.92	43.1	346	626	149	0.116	26980.6
WSxLER	2	2	4	19	39	27	150	41	11.68	47.35	279	180	120	0.041	8523
WSxWS	2	2	4	19	51	23	96	38	10.24	34.85	292	303	98	0.026	10559.55
WSxCVI	2	2	4	19	52	22.5	76	36	11.68	34.55	316.5	709.5	129	0.106	25273.05
WSxC24	2	2	4	30	102	34	58	49	14.2	44.45	449	1459	130	0.423	64852.55
CVI_P	2	2	4	27	68	30	103	43	13.2	35.75	269	367	127	0.167	13120.25
CVIxCOL	2	2	4	22	45	27	96	40	13.64	39.7	320	795	107	0.204	31561.5
CVIxLER	2	2	4	20	54	25	109	41	12.36	21.3	242	591	142	0.09	12588.3
CVIxWS	2	2	4	21	31	27	90	39	10.84	29.15	230	146	97	0.033	4255.9
CVIxCVI	2	2	4	26	45	30	92	43	12.56	29.15	260	215	156	0.054	6267.25
CVIxC24	2	2	4	45	177	49	89	65	14.8	41.85	444	888	120	0.433	37162.8
C24_P	2	2	4	24	43	28	72	43	12.4	38.15	288	467	134	0.1	17816.05
C24xCOL	2	2	4	53	167	56	30	73	11.36	39.35	418	613	106	0.481	24121.55
C24xLER	2	2	4	30	110	34	80	50	12.12	41.9	418	964	99	0.334	40391.6
C24xWS	2	2	4	49	166	53	77	70	11.68	44.15	446	889	132	0.455	39249.35
C24xCVI	2	2	4	39	120	42	75	57	13.08	33.25	438	996	131	0.35	33117
C24xC24	2	2	4	24	43	28	79	43	11.76	38.05	290	456	134	0.087	17350.8
COLxWS	1	2	2	34	103	37	69	53	12.72	46.35	358	1130	154	.	52375.5
WSxCVI	1	2	2	20	53	27	118	41	11.68	44.4	303	436	136	0.041	19358.4
WSxCVI	2	2	4	19	49	24	72	38	11.32	38.85	298	198	114	0.032	7692.3

Col x Ws in block 2 has been substituted with the average from the Col x Ws genotypes in blocks 1, 3 and 4. Ws x Cvi in blocks 2 and 4 have been substituted with the averages of the Ws x Cvi genotypes in blocks 1 and 3. The original values of these three genotypes are listed at the end and shaded in grey. A density value of 1 indicates low density and a density value of 2 indicates high density.

Table S3 Raw data for the number of seeds per silique averaged for the diallel experiment

BLOCK 1	1	2	3	4	5	6	7	8	9	10	11	12	13	14	15	16	17	18	19	20	Average # of Seed / Silique
Col Parent	20	49	39	49	49	40	58	52	54	55	37	51	61	53	47	41	42	53	55	55	48
Col x Col	36	46	17	37	18	40	24	18	41	39	44	58	40	27	33	28	47	36	52	45	36.3
Col x Ler	37	58	49	59	44	56	51	61	54	56	37	51	22	56	46	55	64	60	55	62	51.65
Col x Ws	24	41	24	38	39	46	40	30	33	44	38	33	56	43	50	49	47	57	46	46	41.2
Col x Cvi	41	47	32	39	43	40	29	33	27	32	44	29	35	45	42	40	37	39	34	45	37.65
Col x C24	56	54	59	54	48	32	23	35	46	48	20	44	32	40	43	38	36	43	35	44	41.5
Ler Parent	36	43	43	35	57	35	50	44	54	53	36	39	33	54	48	48	49	47	48	42	44.7
Ler x Col	48	49	44	39	57	48	42	37	38	51	67	55	45	37	46	50	46	37	30	44	45.5
Ler x Ler	25	10	50	27	35	37	31	51	35	36	48	42	51	50	46	12	54	47	46	52	39.25
Ler x Ws	53	32	45	51	50	50	46	44	36	40	34	50	43	48	56	43	42	48	42	38	44.55
Ler x Cvi	28	30	45	37	60	47	44	48	42	36	47	37	47	31	49	47	36	51	37	44	42.15
Ler x C24	23	57	65	60	49	44	58	52	44	30	63	54	48	55	62	51	55	45	57	43	50.75
Ws Parent	38	37	37	36	44	46	40	39	48	28	38	41	34	37	24	40	31	37	30	38	37.15
Ws x Col	36	44	42	56	24	49	38	46	38	42	27	55	14	41	39	38	29	31	47	46	39.1
Ws x Ler	41	35	44	58	46	58	54	36	27	52	55	48	56	46	57	42	52	50	53	54	48.2
Ws x Ws	41	24	27	41	37	35	33	42	34	41	50	39	35	32	50	35	47	31	48	37	37.95
Ws x Cvi	29	24	37	34	42	29	47	33	40	39	44	30	35	31	41	37	39	40	45	28	36.2
Ws x C24	45	41	15	51	47	50	39	52	45	52	51	50	45	54	56	47	51	47	51	49	46.9
Cvi Parent	9	19	26	34	39	46	29	48	34	28	40	32	37	33	29	23	24	30	14	42	30.8
Cvi x Col	33	27	39	13	40	41	44	31	35	36	39	40	38	40	28	35	41	39	24	42	35.25
Cvi x Ler	3	11	16	40	29	41	36	37	36	46	36	42	31	43	39	42	32	37	45	36	33.9
Cvi x Ws	27	21	25	34	32	28	34	28	27	29	19	20	31	26	32	33	25	30	20	28	27.45
Cvi x Cvi	39	12	11	30	9	24	19	28	10	24	22	28	18	29	31	44	32	36	24	27	24.85
Cvi x C24	53	40	39	49	42	37	40	37	40	44	33	41	48	48	37	39	37	45	47	46	42.1
C24 Parent	35	32	47	44	39	47	43	40	39	37	39	42	33	39	37	40	41	38	34	37	39.15
C24 x Col	57	21	28	20	51	50	50	55	20	45	43	37	39	52	34	47	36	49	45	37	40.8
C24 x Ler	16	58	40	57	52	59	60	57	65	43	46	36	32	61	53	56	50	58	50	45	49.7
C24 x Ws	50	46	58	35	37	52	31	47	39	43	48	47	52	39	51	58	58	50	48	49	46.9
C24 x Cvi	45	48	43	35	48	44	43	50	31	35	42	57	30	35	46	27	42	43	32	46	41.1
C24 x C24	23	33	35	38	40	42	40	43	41	31	41	29	37	46	42	34	35	43	29	34	36.8

BLOCK 2	1	2	3	4	5	6	7	8	9	10	11	12	13	14	15	16	17	18	19	20	Average # of Seed / Silique
Col Parent	9	46	33	38	42	21	44	8	35	18	53	40	53	52	55	50	51	44	40	50	39.1
Col x Col	22	46	40	8	53	45	45	65	55	41	46	33	35	36	52	42	46	53	53	50	43.3
Col x Ler	57	57	60	61	63	39	43	57	59	52	44	57	55	53	62	66	57	61	64	58	56.25
Col x Ws	52	47	53	56	39	45	37	48	52	45	53	36	46	43	45	50	37	47	46	50	46.35
Col x Cvi	39	15	15	39	29	41	42	32	46	44	46	41	35	41	35	49	44	32	38	47	37.5
Col x C24	37	40	54	43	54	58	47	56	62	53	57	54	40	41	45	34	30	51	56	29	47.05
Ler Parent	52	39	46	38	30	36	40	38	28	29	36	32	39	34	8	39	40	32	24	35	34.75
Ler x Col	41	47	24	58	51	59	53	52	56	64	53	53	55	60	55	59	55	48	47	57	52.35
Ler x Ler	48	46	33	46	40	52	34	45	36	21	19	42	24	34	21	31	18	25	26	26	33.35
Ler x Ws	23	23	35	45	34	46	45	46	55	50	39	45	57	33	35	43	44	58	44	50	42.5
Ler x Cvi	35	30	33	46	49	40	46	47	53	22	26	36	47	45	46	37	35	37	32	40	39.1
Ler x C24	51	60	61	57	57	55	62	56	63	50	56	52	41	59	50	47	51	39	49	53	53.45
Ws Parent	33	34	37	19	31	26	37	37	35	33	27	22	42	36	45	47	38	38	55	41	35.65
Ws x Col	48	53	55	47	59	46	45	51	50	40	34	53	41	54	50	46	47	42	49	48	47.9
Ws x Ler	36	48	49	49	45	52	50	48	52	57	53	59	60	54	63	52	54	39	54	55	51.45
Ws x Ws	30	26	37	36	39	39	34	44	44	22	30	20	25	35	28	41	42	33	33	36	33.7
Ws x Cvi	44	47	44	45	42	48	48	50	42	40	41	49	40	45	42	41	43	49	38	50	44.4
Ws x C24	32	38	23	58	46	31	59	56	50	53	55	46	49	58	55	62	54	50	43	48	48.3
Cvi Parent	17	12	21	43	34	32	24	32	36	30	13	39	27	29	25	28	35	36	31	20	28.2
Cvi x Col	36	27	41	46	38	33	37	42	46	41	43	41	11	46	45	39	41	34	38	39	38.2
Cvi x Ler	38	38	38	32	39	40	35	45	36	38	37	39	48	39	25	42	45	43	30	32	37.95
Cvi x Ws	34	36	21	36	44	40	36	37	51	35	22	38	28	38	30	37	40	33	26	42	35.2
Cvi x Cvi	35	33	34	29	40	35	13	37	26	32	25	20	39	33	34	33	24	26	34	28	30.5
Cvi x C24	24	41	42	42	40	25	24	44	34	36	41	44	29	44	47	38	49	41	29	43	37.85
C24 Parent	40	38	28	40	44	31	33	22	27	30	32	29	26	34	35	31	36	32	31	30	32.45
C24 x Col	45	48	48	51	34	47	34	39	55	56	54	54	51	40	44	55	49	53	48	49	47.7
C24 x Ler	63	58	64	56	65	53	50	58	58	59	50	56	54	56	53	62	56	60	63	60	57.7
C24 x Ws	51	49	57	55	52	50	56	55	51	48	34	50	39	51	47	43	45	45	47	44	48.45
C24 x Cvi	39	26	43	53	49	19	33	49	50	45	43	33	54	48	34	45	42	45	35	41	41.3
C24 x C24	44	42	22	47	44	45	43	45	30	36	32	26	27	29	33	34	38	43	37	39	36.8

BLOCK 3	1	2	3	4	5	6	7	8	9	10	11	12	13	14	15	16	17	18	19	20	Average # of Seed / Silique
Col Parent	4	8	3	48	48	43	45	45	44	44	56	41	52	54	45	43	42	39	54	46	40.2

Col x Col	37	35	34	43	42	38	36	63	48	52	43	49	45	16	36	39	40	44	34	38	40.6
Col x Ler	54	64	23	56	4	65	37	57	65	43	61	53	63	63	63	64	63	42	54	60	52.7
Col x Ws	31	43	32	36	9	54	22	44	48	20	21	52	37	55	33	26	42	39	51	49	37.2
Col x Cvi	56	36	40	18	31	49	33	49	32	22	36	39	34	37	44	45	38	41	33	37	37.5
Col x C24	59	51	50	51	53	57	39	48	47	54	42	48	48	47	46	55	45	46	42	35	48.15
Ler Parent	21	34	49	36	44	44	47	38	42	40	39	26	43	43	50	44	46	49	31	42	40.4
Ler x Col	54	29	49	38	45	49	43	41	49	40	26	49	43	48	50	51	58	54	46	53	45.75
Ler x Ler	70	35	41	30	45	40	41	41	46	43	34	41	41	43	47	29	40	35	47	35	41.2
Ler x Ws	48	45	44	34	41	30	43	45	34	27	43	41	49	37	35	55	45	46	55	41	41.9
Ler x Cvi	44	33	44	34	21	51	45	45	42	43	44	47	50	35	40	50	38	42	51	43	42.1
Ler x C24	45	64	49	58	58	62	62	62	64	68	63	51	53	61	61	53	58	69	66	45	58.6
Ws Parent	34	36	38	37	32	27	36	34	42	37	40	35	35	36	34	41	33	46	33	41	36.35
Ws x Col	18	23	32	49	47	52	53	54	56	56	50	45	50	54	56	41	57	48	44	47	46.6
Ws x Ler	35	47	38	41	26	47	56	54	45	44	46	45	56	56	40	49	46	50	47	34	45.1
Ws x Ws	36	45	25	40	28	38	40	29	45	36	24	37	35	35	45	39	41	39	30	41	36.4
Ws x Cvi	35	40	32	35	22	39	33	38	37	18	24	31	27	37	34	28	30	43	38	37	32.9
Ws x C24	45	49	32	37	57	50	36	51	38	52	42	48	43	48	59	45	59	50	52	61	47.7
Cvi Parent	21	22	35	27	19	29	28	31	28	15	25	30	16	36	14	43	29	26	25	16	25.75
Cvi x Col	27	46	41	40	38	42	46	43	47	32	37	48	41	52	35	46	38	39	29	42	40.45
Cvi x Ler	18	25	50	17	41	7	41	30	40	17	35	37	39	47	41	37	39	45	49	45	35
Cvi x Ws	21	16	27	30	30	28	27	38	24	35	19	21	38	33	25	20	32	17	23	30	26.7
Cvi x Cvi	38	23	31	34	41	32	34	22	14	10	19	21	20	32	32	28	42	36	38	39	29.3
Cvi x C24	41	38	25	32	47	45	30	39	23	43	49	40	43	40	43	44	51	41	45	46	40.25
C24 Parent	45	46	33	32	35	33	45	40	21	34	41	37	41	45	43	31	43	44	41	35	38.25
C24 x Col	54	46	44	36	55	33	26	25	28	52	45	47	51	31	52	46	52	59	43	47	43.6
C24 x Ler	53	55	62	63	55	65	65	53	34	39	59	58	50	51	42	64	56	54	58	59	54.75
C24 x Ws	57	52	56	50	60	54	57	17	27	54	58	57	50	61	37	47	55	50	45	51	49.75
C24 x Cvi	38	26	43	25	52	41	42	41	47	36	39	35	33	46	31	47	45	44	51	42	40.2
C24 x C24	40	21	42	15	38	16	41	39	40	23	30	24	40	30	29	35	41	37	37	42	33

BLOCK 4

	1	2	3	4	5	6	7	8	9	10	11	12	13	14	15	16	17	18	19	20	Average # of Seed / Silique
Col Parent	4	33	23	42	26	61	54	47	30	45	31	15	48	57	41	56	54	57	50	51	41.25
Col x Col	19	47	39	48	53	53	56	56	48	57	54	56	31	51	18	46	44	42	51	46	45.75
Col x Ler	42	51	45	45	39	38	50	58	56	50	53	48	49	50	54	53	47	43	43	52	48.3
Col x Ws	24	37	37	31	34	28	47	53	47	48	46	49	48	51	47	46	43	43	44	43	42.3

Col x Cvi	23	45	11	28	42	27	48	42	42	35	56	40	19	44	28	42	39	31	34	41	35.85
Col x C24	26	22	43	47	33	39	35	25	41	38	43	47	54	49	41	46	45	46	38	50	40.4
Ler Parent	38	33	28	33	43	27	34	38	39	25	26	37	10	28	22	30	23	31	14	36	29.75
Ler x Col	30	50	28	45	44	53	46	54	34	44	55	53	56	35	45	48	41	36	53	34	44.2
Ler x Ler	37	37	21	34	27	26	34	33	28	20	31	28	27	29	21	28	28	14	24	25	27.6
Ler x Ws	48	53	41	44	36	33	37	49	48	50	45	46	44	50	37	35	40	58	57	44	44.75
Ler x Cvi	16	17	38	38	40	43	28	40	34	44	38	45	41	44	47	42	41	43	46	28	37.65
Ler x C24	54	50	59	63	66	59	62	64	65	45	53	55	54	49	46	46	50	49	54	52	54.75
Ws Parent	34	34	30	32	40	31	24	33	34	35	34	38	37	25	34	32	29	37	35	32	33
Ws x Col	19	36	50	42	46	42	52	52	48	45	51	52	38	49	25	46	47	45	40	37	43.1
Ws x Ler	8	47	38	47	53	43	57	60	38	55	56	54	39	57	47	50	59	48	51	40	47.35
Ws x Ws	36	4	26	20	34	37	44	42	45	39	36	46	41	45	30	34	42	31	31	34	34.85
Ws x Cvi	37	35	27	48	41	31	43	42	46	38	33	40	29	32	32	41	48	46	44	44	38.85
Ws x C24	21	46	31	41	28	49	36	48	54	57	51	57	50	33	54	50	54	34	46	49	44.45
Cvi Parent	34	43	39	40	30	28	33	27	41	39	32	45	45	41	44	24	31	31	33	35	35.75
Cvi x Col	37	44	45	39	38	34	41	40	40	45	35	34	43	33	39	36	40	40	43	48	39.7
Cvi x Ler	2	21	21	24	34	19	27	22	9	28	21	26	19	22	16	33	15	28	17	22	21.3
Cvi x Ws	28	26	43	32	34	29	28	27	22	27	29	30	24	27	36	24	29	29	37	22	29.15
Cvi x Cvi	14	21	9	16	38	27	35	36	23	39	22	36	34	40	37	34	29	43	27	23	29.15
Cvi x C24	42	56	44	38	41	47	41	47	40	47	45	47	38	40	35	37	39	35	38	40	41.85
C24 Parent	16	45	52	45	38	36	35	40	24	39	41	43	43	29	42	41	30	48	40	36	38.15
C24 x Col	30	42	28	38	48	37	35	42	32	45	41	27	37	41	51	40	41	50	38	44	39.35
C24 x Ler	35	33	51	47	46	54	35	36	42	60	44	46	52	44	35	37	41	26	40	34	41.9
C24 x Ws	39	46	53	45	43	34	45	38	33	40	49	38	41	53	48	49	51	50	45	43	44.15
C24 x Cvi	33	36	34	36	35	23	36	28	30	31	26	34	36	34	29	39	37	36	38	34	33.25
C24 x C24	17	44	45	40	24	56	34	40	32	40	31	50	36	36	47	43	35	42	31	38	38.05

Table S4 Raw data for the average silique length in the diallel experiment

BLOCK 1	1	2	3	4	5	6	7	8	9	10	11	12	13	14	15	16	17	18	19	20	21	22	23	24	25	Average Silique length
Col Parent	12	12	11	13	12	12	12	12	12	13	12	12	13	13	11	12	11	13	11	12	12	13	12	12	12	12.08
Col x Col	12	11	12	10	10	9	8	11	10	13	12	10	12	10	10	10	11	11	10	12	11	11	10	12	11	10.76
Col x Ler	12	14	15	15	13	14	14	14	14	13	15	15	13	12	13	13	13	13	12	14	13	12	12	13	12	13.32
Col x Ws	10	11	11	11	10	10	10	12	12	11	11	10	11	11	12	11	12	11	11	10	11	12	10	11	10	10.88
Col x Cvi	13	15	16	14	14	16	13	12	14	15	15	12	13	14	15	13	14	15	15	14	14	16	13	11	14	14
Col x C24	14	13	15	13	14	14	10	13	9	8	11	12	12	13	11	12	11	14	10	12	10	13	13	15	14	12.24
Ler Parent	10	10	10	9	9	10	9	10	10	10	8	9	8	9	8	10	10	10	10	10	9	10	9	9	10	9.44
Ler x Col	15	12	14	13	14	14	12	14	13	11	14	12	11	12	13	13	14	11	11	11	12	11	11	12	12	12.48
Ler x Ler	11	10	10	9	10	11	9	9	9	9	8	8	10	9	7	9	7	8	9	9	8	8	8	7	8	8.8
Ler x Ws	12	11	10	12	9	12	11	13	11	11	13	13	13	13	12	10	14	14	11	12	9	9	11	10	10	11.44
Ler x Cvi	15	13	13	16	16	15	14	16	15	15	14	16	15	15	16	15	15	15	16	13	15	14	12	16	16	14.84
Ler x C24	11	16	16	16	13	16	13	13	14	16	14	12	13	14	13	13	14	13	14	13	13	11	12	13	12	13.52
Ws Parent	11	10	11	12	11	10	10	9	12	10	7	12	11	10	11	12	12	11	9	8	10	10	10	10	10	10.36
Ws x Col	12	12	13	10	15	10	14	13	13	12	15	11	11	12	13	13	14	14	13	13	13	13	14	12	13	12.72
Ws x Ler	14	14	11	12	12	12	11	13	14	10	12	14	11	11	11	12	13	12	13	12	11	13	14	12	14	12.32
Ws x Ws	11	12	11	11	9	10	11	11	11	10	12	13	10	11	12	11	10	11	10	10	11	12	10	12	11	10.92
Ws x Cvi	14	14	11	13	12	11	13	12	12	12	11	11	14	11	12	11	12	11	14	12	14	13	13	13	9	12.2
Ws x C24	14	14	11	14	13	15	14	15	15	14	13	14	14	14	15	13	13	14	13	13	15	13	13	13	14	13.72
Cvi Parent	12	15	14	16	17	13	16	15	16	16	14	11	12	8	11	14	12	15	11	12	14	16	15	12	13	13.6
Cvi x Col	13	13	16	10	13	14	14	12	13	15	13	14	14	13	14	12	12	12	14	13	14	12	14	12	12	13.12
Cvi x Ler	14	14	12	11	15	12	12	12	11	12	10	14	11	11	11	11	12	10	10	11	13	14	14	10	11	11.92
Cvi x Ws	11	12	11	12	13	8	12	12	13	12	12	11	12	11	9	11	12	11	11	10	11	11	10	10	10	11.12
Cvi x Cvi	19	12	12	11	8	9	13	13	11	13	8	10	11	15	16	12	13	11	12	12	11	10	13	15	12	12.08
Cvi x C24	17	16	16	18	15	15	15	16	15	16	14	16	15	14	15	15	15	14	16	15	16	16	15	15	17	15.48
C24 Parent	15	14	13	15	13	12	13	12	13	12	13	13	11	10	12	12	11	12	12	11	11	12	14	13	12	12.44
C24 x Col	13	10	11	12	11	12	10	13	8	10	12	11	9	12	12	13	10	12	11	13	11	12	12	12	12	11.36
C24 x Ler	18	16	16	18	18	15	16	17	16	12	14	15	15	15	14	13	14	13	14	12	13	12	14	11	12	14.52
C24 x Ws	15	16	14	14	14	13	12	11	14	13	13	13	14	13	13	12	13	14	12	12	13	11	13	10	12	12.96
C24 x Cvi	17	17	17	15	16	16	17	16	13	14	15	16	13	13	14	13	17	15	15	14	18	17	17	16	15	15.44
C24 x C24	16	16	14	14	13	14	12	12	13	13	12	13	13	14	13	12	11	12	12	11	12	12	12	13	11	12.8

BLOCK 2

	1	2	3	4	5	6	7	8	9	10	11	12	13	14	15	16	17	18	19	20	21	22	23	24	25	**Average Silique length**
Col Parent	11	13	11	7	9	13	10	14	13	13	13	13	12	11	12	12	12	11	12	11	10	10	11	11	11	11.44
Col x Col	11	11	12	8	11	11	12	11	12	11	13	12	13	12	9	10	10	10	11	11	12	11	9	11	11	11
Col x Ler	15	13	13	10	11	14	14	15	13	15	14	13	14	14	16	14	13	12	12	12	12	11	11	12	14	13.08
Col x Ws	15	12	16	14	13	11	11	13	12	11	14	10	13	14	12	12	14	10	11	14	14	12	14	12	14	12.72
Col x Cvi	16	15	14	13	14	13	15	15	15	16	12	13	11	14	12	14	14	14	14	13	13	16	14	14	13	13.88
Col x C24	15	13	16	14	15	12	14	16	14	16	14	15	15	13	15	15	10	14	12	11	9	10	13	15	12	13.52
Ler Parent	9	9	9	6	9	7	9	10	9	9	10	8	8	8	7	8	10	9	8	8	9	9	7	9	8	8.48
Ler x Col	12	12	12	14	13	13	14	14	13	13	13	15	13	14	12	12	14	13	12	12	12	12	12	11	12	12.76
Ler x Ler	10	10	10	10	10	11	9	9	7	6	5	7	7	6	5	7	5	5	5	8	5	4	.	.	.	7.32
Ler x Ws	13	13	13	11	13	15	13	13	11	13	12	15	12	14	12	11	11	14	12	12	10	13	11	11	10	12.32
Ler x Cvi	15	14	14	15	14	16	15	15	15	11	14	15	15	15	12	13	13	12	14	15	15	12	13	12	11	13.8
Ler x C24	15	15	12	14	12	14	13	13	14	13	13	13	14	13	14	14	15	14	13	13	13	15	13	12	12	13.44
Ws Parent	8	8	9	9	9	10	9	10	9	9	10	10	8	9	8	9	7	8	8	10	8	7	9	9	8	8.72
Ws x Col	14	13	14	13	14	13	13	14	14	13	15	15	12	9	13	13	12	15	14	14	13	13	13	14	13	13.32
Ws x Ler	13	12	13	11	14	9	13	12	11	12	13	10	13	11	11	11	12	12	10	9	11	11	10	11	10	11.4
Ws x Ws	10	9	10	10	9	8	7	10	11	11	10	9	11	10	10	8	10	10	10	9	9	11	10	8	9	9.56
Ws x Cvi	12	11	11	12	12	12	12	11	11	11	12	12	13	13	12	13	12	11	11	12	11	11	11	11	12	11.68
Ws x C24	13	15	10	15	12	15	14	13	13	15	15	13	11	12	13	13	14	13	13	15	17	15	17	15	14	13.8
Cvi Parent	9	11	15	14	13	12	12	13	11	8	14	12	12	10	11	10	10	12	11	11	10	10	11	9	9	11.2
Cvi x Col	14	15	14	14	15	13	14	15	8	14	14	13	12	14	14	13	13	13	13	13	13	13	12	12	13	13.24
Cvi x Ler	11	12	11	13	13	12	12	11	13	11	13	15	11	11	15	11	12	15	11	11	10	11	14	15	15	12.36
Cvi x Ws	14	14	14	12	13	13	15	15	10	15	13	16	15	14	13	12	13	15	13	12	12	12	13	13	12	13.32
Cvi x Cvi	13	15	14	9	15	15	15	13	12	16	12	12	13	12	12	12	14	11	8	11	9	13	12	12	13	12.52
Cvi x C24	10	16	15	14	16	16	15	15	15	14	13	15	14	16	16	14	15	16	16	15	16	15	15	15	15	14.88
C24 Parent	13	15	14	14	14	11	12	11	12	11	12	13	13	12	12	11	10	10	11	10	10	10	10	8	11	11.6
C24 x Col	14	11	12	13	13	12	11	13	13	11	14	12	14	13	13	14	13	11	12	13	13	12	12	14	13	12.64
C24 x Ler	12	15	15	14	13	12	14	13	13	12	14	13	14	12	16	13	14	15	15	15	16	15	16	15	16	14.08
C24 x Ws	16	14	15	15	13	15	12	14	12	13	14	12	12	12	12	13	13	13	12	12	9	12	14	11	11	12.84
C24 x Cvi	14	15	11	10	16	15	16	16	17	16	15	15	13	14	16	15	14	13	13	15	14	16	14	14	14	14.44
C24 x C24	11	15	15	15	15	13	13	14	12	12	12	13	13	12	11	10	13	12	11	13	9	11	12	13	12	12.48

BLOCK 3

	1	2	3	4	5	6	7	8	9	10	11	12	13	14	15	16	17	18	19	20	21	22	23	24	25	**Average Silique Length**
Col Parent	13	12	15	13	12	13	14	13	14	12	11	13	11	12	14	13	13	12	11	13	12	12	12	12	13	12.6

Col x Col	13	11	12	11	12	12	12	9	11	12	8	10	11	13	10	12	8	12	11	12	12	9	11	12	9	11
Col x Ler	11	14	15	12	14	12	12	14	11	13	12	13	12	11	13	13	12	13	12	11	10	14	14	11	14	12.52
Col x Ws	11	12	15	14	13	14	14	13	13	14	14	13	12	11	13	13	13	14	12	12	12	13	13	13	13	12.96
Col x Cvi	10	13	13	16	13	14	15	15	16	14	14	13	16	12	12	9	13	13	13	14	14	13	14	14	10	13.32
Col x C24	12	14	13	13	12	10	14	12	12	13	12	12	13	13	14	12	11	12	12	12	9	8	12	13	13	12.12
Ler Parent	8	10	11	10	9	11	10	9	9	10	10	9	9	9	9	9	9	9	9	9	7	9	9	9	8	9.2
Ler x Col	13	12	14	15	15	13	13	13	13	12	14	13	12	13	14	12	12	11	14	11	11	11	11	11	12	12.6
Ler x Ler	11	10	10	8	9	9	9	9	10	9	10	8	8	8	8	8	10	8	10	9	9	9	10	8	8	9
Ler x Ws	12	10	11	14	14	15	13	14	12	13	13	10	13	11	14	13	12	11	13	12	12	11	11	12	12	12.32
Ler x Cvi	14	15	15	15	15	15	12	16	13	15	14	15	16	13	14	12	12	15	14	13	14	13	16	15	14	14.2
Ler x C24	14	16	15	16	16	15	15	15	15	15	15	15	16	16	16	16	14	13	14	15	15	13	13	16	16	15
Ws Parent	11	10	10	11	12	11	11	11	11	12	12	10	11	11	12	11	11	11	10	9	9	9	9	11	9	10.6
Ws x Col	13	14	13	15	15	13	15	14	14	15	13	12	13	14	12	12	13	13	15	14	12	13	12	12	11	13.28
Ws x Ler	12	12	12	13	11	10	13	11	11	10	10	13	12	8	8	8	13	12	13	12	10	11	8	9	9	10.84
Ws x Ws	11	9	12	12	10	10	10	11	11	11	11	11	10	10	10	10	10	11	10	10	9	10	9	12	9	10.36
Ws x Cvi	10	11	12	10	11	11	12	12	13	13	11	12	11	11	11	10	12	12	11	9	10	11	10	11	12	11.16
Ws x C24	15	14	16	15	14	15	14	15	14	17	14	15	16	14	13	14	15	13	15	14	15	15	15	15	14	14.64
Cvi Parent	14	10	13	11	11	13	12	14	13	9	11	12	11	10	15	9	14	10	12	16	15	14	13	13	12	12.28
Cvi x Col	15	15	16	16	13	15	15	13	15	14	15	15	15	15	14	14	15	15	14	14	13	15	14	14	14	14.52
Cvi x Ler	13	16	15	15	14	12	13	11	12	11	13	15	10	12	14	13	14	13	15	16	14	13	11	10	12	13.08
Cvi x Ws	8	11	11	11	12	9	11	11	11	12	11	9	10	10	11	11	10	10	11	12	11	10	8	9	10	10.4
Cvi x Cvi	15	13	11	12	15	14	16	13	10	12	13	11	8	10	11	13	13	16	12	14	12	14	13	11	14	12.64
Cvi x C24	14	13	12	12	15	16	13	13	10	14	14	14	14	15	15	14	15	13	14	15	14	12	16	16	16	13.96
C24 Parent	14	14	15	11	13	11	10	11	9	11	13	12	13	12	12	13	9	13	15	13	13	13	12	14	14	12.4
C24 x Col	15	13	11	13	8	10	10	8	15	14	12	13	13	14	9	12	11	13	13	13	11	12	12	13	14	12.08
C24 x Ler	16	15	14	15	15	15	15	14	14	14	13	12	13	13	14	14	13	14	13	14	13	14	14	14	13	13.92
C24 x Ws	13	14	16	15	15	14	14	15	14	11	9	12	13	14	11	11	13	12	12	13	11	12	13	14	11	12.88
C24 x Cvi	13	12	16	14	15	18	16	16	16	17	14	15	16	13	14	13	14	15	18	16	14	14	16	15	16	15.04
C24 x C24	11	16	13	10	14	13	10	14	10	12	12	10	12	12	12	12	13	12	11	10	11	12	12	11	12	11.88

BLOCK 4

	1	2	3	4	5	6	7	8	9	10	11	12	13	14	15	16	17	18	19	20	21	22	23	24	25	**Average Silique Length**
Col Parent	13	13	10	12	11	12	13	13	14	13	11	12	12	12	12	11	8	12	12	12	13	13	13	12	13	12.08
Col x Col	13	14	14	12	12	14	8	12	12	11	11	13	12	11	10	11	11	10	12	11	12	14	12	12	11	11.8
Col x Ler	13	13	13	13	13	13	11	12	11	12	12	12	12	13	13	12	12	12	12	11	12	13	12	13	11	12.24
Col x Ws	13	12	13	12	13	13	11	12	13	12	11	11	11	11	11	11	10	11	10	11	11	12	11	10	10	11.44

Col x Cvi	15	15	11	13	14	13	13	14	12	14	14	12	14	13	13	13	15	14	10	13	10	11	12	14	12	12.96
Col x C24	11	11	13	12	12	9	11	13	12	9	11	12	12	14	13	11	11	13	12	13	12	14	13	13	11	11.92
Ler Parent	8	8	7	7	8	8	7	8	7	7	6	6	5	8	5	6	7	7	6	6	5	4	7	7	5	6.6
Ler x Col	12	10	11	13	13	10	14	10	11	11	11	11	12	10	10	11	10	11	10	10	10	9	10	9	9	10.72
Ler x Ler	8	8	7	8	7	9	7	7	7	8	7	8	8	6	6	7	7	7	7	6	7	6	6	7	6	7.08
Ler x Ws	12	10	14	11	12	12	11	12	11	11	12	13	10	13	11	12	10	12	13	11	14	14	11	10	13	11.8
Ler x Cvi	13	14	13	14	15	15	13	16	15	15	13	14	15	13	15	10	14	14	14	14	14	13	10	13	12	13.64
Ler x C24	15	13	14	13	16	15	14	16	13	11	14	14	14	13	14	13	13	14	15	13	13	15	14	13	14	13.84
Ws Parent	10	11	9	10	9	8	10	11	10	10	9	10	10	9	8	8	9	8	9	9	11	8	9	8	10	9.32
Ws x Col	15	14	14	13	13	13	13	14	15	13	12	12	12	13	13	12	13	13	12	13	13	13	12	13	10	12.92
Ws x Ler	10	12	13	12	13	13	12	13	12	12	12	12	13	12	12	11	11	12	11	11	10	10	10	12	11	11.68
Ws x Ws	10	10	11	11	11	9	10	11	10	12	10	11	11	11	11	11	10	9	9	10	10	10	9	10	9	10.24
Ws x Cvi	13	13	14	14	12	13	11	12	11	11	12	12	11	11	11	8	11	12	11	11	10	9	11	9	10	11.32
Ws x C24	14	10	14	12	13	15	12	16	15	15	15	16	15	15	15	15	13	15	13	15	14	16	13	14	15	14.2
Cvi Parent	12	16	15	13	13	13	12	12	14	12	14	10	14	13	15	13	13	14	14	16	12	11	17	10	12	13.2
Cvi x Col	15	15	14	16	15	15	14	15	13	14	15	13	15	11	14	14	13	12	12	13	12	12	14	12	13	13.64
Cvi x Ler	9	10	13	8	13	13	15	13	13	13	13	15	10	15	13	14	12	12	11	13	14	14	12	9	12	12.36
Cvi x Ws	13	12	12	12	12	12	11	12	12	10	9	11	11	10	10	10	9	10	11	11	10	10	10	11	10	10.84
Cvi x Cvi	12	12	10	12	13	14	11	14	14	10	11	12	13	14	13	13	12	12	13	14	13	13	13	13	13	12.56
Cvi x C24	13	17	15	15	13	14	16	14	15	15	15	15	15	15	16	15	14	13	15	15	14	15	15	15	16	14.8
C24 Parent	13	14	15	15	14	13	12	12	11	12	13	12	10	12	13	12	12	12	12	12	13	11	12	11	12	12.4
C24 x Col	11	12	13	12	11	10	12	10	9	11	11	12	10	8	10	13	13	12	11	11	11	14	12	13	12	11.36
C24 x Ler	10	12	13	12	11	13	15	15	12	11	11	12	10	11	11	11	13	12	14	13	11	12	13	13	12	12.12
C24 x Ws	10	13	15	12	12	11	10	11	9	11	12	13	11	12	12	13	12	13	13	12	10	11	11	11	12	11.68
C24 x Cvi	14	13	14	11	13	13	14	13	14	13	13	12	14	13	14	13	12	13	12	13	12	14	13	13	14	13.08
C24 x C24	14	14	15	12	12	13	11	11	10	11	12	12	10	11	12	12	11	12	12	10	10	12	11	12	12	11.76

Silique length measurements are in millimeters.

Table S5 Raw data from the *FRI* and *FLC* experiment

Genotype FRI/FLC	Block	Bolting Date (DAS)	Rosette Diameter (mm)	Flowering Date (DAS)	Flowering Height (mm)	Maturity Date (DAS)	Final Height (mm)	Lifespan (days)	Stem Weight (grams)	Average Silique Length	Average Number of Seeds per pod	Total Number of Siliques	Estimated Total Number of Seeds
Col-/-	1	19	44	24	49	36	430	137	0.567	14.8	52.8	953	50318.4
Col-/+	1	20	36	26	50	39	414	158	0.487	14.2	55.6	1031	57323.6
Col+/-	1	20	45	25	47	39	394	154	0.49	14.6	52.2	1088	56793.6
Col+/+	1	54	154	59	41	78	517	186	0.894	10.8	28.4	245	6958
Ler-/-	1	19	30	23	34	37	219	108	0.147	10.4	46.4	364	16889.6
Ler-/+C	1	21	42	27	25	44	245	148	0.354	12.8	54.4	825	44880
Ler-/+S	1	21	40	27	21	41	278	117	0.3	12	54.2	673	36476.6
Ler+/-	1	21	37	27	20	42	269	134	0.309	13	60.4	622	37568.8
Ler+/+	1	77	121	83	17	105	192	202	0.45	9.25	31.25	169	5281.25
C24	1	34	72	39	120	53	433	156	0.388	14.2	46	744	34224
C24xCol-/-	1	21	51	27	42	40	441	147	0.621	17.6	62.4	992	61900.8
C24xCol-/+	1	.	.	.	.	.	.	.	.	.	.	.	.
C24xLer-/-	1	23	67	27	42	42	432	150	0.396	17.4	58.8	732	43041.6
C24xLer-/+	1	46	146	52	41	68	548	183	0.8	17	54	978	52812
Col-/-	2	19	55	23	55	36	379	144	0.443	15.2	51.8	1063	55063.4
Col-/+	2	20	37	25	44	38	366	154	0.456	14.6	53.8	935	50303
Col+/-	2	21	36	26	51	37	401	145	0.567	13.6	49.4	979	48362.6
Col+/+	2	40	128	44	31	60	614	196	0.791	13.2	53.8	836	44976.8
Ler-/-	2	19	23	23	21	37	233	110	0.132	11.2	54.4	327	17788.8
Ler-/+C	2	20	34	25	16	40	255	140	0.285	11.2	49.2	749	36850.8
Ler-/+S	2	21	29	27	19	41	294	119	0.26	11.8	54.8	527	28879.6
Ler+/-	2	23	44	29	12	44	257	115	0.286	13.4	61.8	395	24411
Ler+/+	2	60	132	68	23	.	.	.	.	.	.	.	.
C24	2	34	66	37	123	52	310	140	0.456	11.4	25.6	996	25497.6
C24xCol-	2	25	70	30	70	43	448	151	0.609	16	55.6	1100	61160
C24xCol+	2	40	115	44	29	58	465	188	0.496	15.2	50.2	693	34788.6
C24xLer-	2	28	89	34	78	48	436	155	0.449	15.6	57.4	730	41902
C24xLer+	2	47	134	53	19	68	496	204	0.729	15.4	47.8	927	44310.6

Col-/-	3	20	41	25	43	37	389	143	0.521	14.6	49.6	1090	54064
Col-/+	3	21	45	27	39	40	395	153	0.436	15.4	57.8	991	57279.8
Col+/-	3	20	38	25	40	38	398	146	0.441	15	53	744	39432
Col+/+	3	44	126	48	11	68	676	176	0.947	11.8	28.8	406	11692.8
Ler-/-	3	19	28	23	36	37	220	70	0.106	10.6	47.2	234	11044.8
Ler-/+C	3	21	24	26	29	41	239	145	0.228	12.2	51	669	34119
Ler-/+S	3	30	71	35	80	50	263	118	0.299	11.4	57.6	692	39859.2
Ler+/-	3	30	72	35	92	50	236	144	0.34	12.8	63	814	51282
Ler+/+	3	67	107	73	14	93	294	182	0.357	10.6	49.8	389	19372.2
C24	3	33	69	37	126	51	413	139	0.319	14.4	46.2	700	32340
C24xCol-	3	24	74	29	27	43	472	155	0.752	16.8	57.2	1288	73673.6
C24xCol+	3	44	137	47	48	60	569	192	0.611	13.8	49.8	842	41931.6
C24xLer-	3	25	64	31	72	45	449	146	0.443	15	53.4	627	33481.8
C24xLer+	3	45	122	51	23	66	523	209	0.756	15.8	56.8	923	52426.4
Col-/-	4	19	43	23	29	37	385	144	0.493	14.4	52.4	783	41029.2
Col-/+	4	21	36	26	34	39	432	138	0.409	14.8	57.4	1001	57457.4
Col+/-	4	23	46	29	86	42	377	146	0.565	14.6	56.8	1179	66967.2
Col+/+	4	72	148	78	26	94	511	142	0.659	.	.	.	.
Ler-/-	4	19	26	23	39	37	238	95	0.075	10.8	45	224	10080
Ler-/+C	4	19	38	23	36	37	229	135	0.269	11.2	46.8	837	39171.6
Ler-/+S	4	20	25	25	28	38	277	121	0.253	10.2	45.2	643	29063.6
Ler+/-	4	27	62	33	28	49	260	133	0.418	13	66	943	62238
Ler+/+	4	67	121	75	33	91	258	183	0.335	11.2	52.6	455	23933
C24	4	36	72	41	134	54	414	145	0.381	14.4	47.4	648	30715.2
C24xCol-	4	23	64	29	49	42	461	149	0.784	18.6	62.6	1414	88516.4
C24xCol+	4	48	109	53	14	68	487	198	0.468	15.8	52	734	38168
C24xLer-	4	27	95	33	118	47	436	131	0.534	15.4	60	952	57120
C24xLer+	4	48	137	53	20	69	567	184	1.013	14.8	53.6	985	52796
Col-/-	5	19	48	24	42	37	337	134	0.389	15.6	56	1120	62720
Col-/+	5	20	46	25	48	39	378	148	0.487	13.2	50.6	1015	51359
Col+/-	5	20	44	25	34	39	383	144	0.437	14	51.6	988	50980.8
Col+/+	5	47	155	52	16	69	676	180	0.898	12.6	48	525	25200
Ler-/-	5	19	22	23	14	37	162	83	0.057	10	43.8	124	5431.2
Ler-/+C	5	21	31	27	18	42	245	141	0.266	11.6	52.4	816	42758.4

Ler-/+S	5	22	31	28	15	42	258	98	0.088	11.8	54.4	216	11750.4
Ler+/-	5	24	42	31	71	49	277	108	0.275	11.6	56.8	551	31296.8
Ler+/+	5	65	113	71	19	90	281	156	0.419	11.4	46.4	446	20694.4
C24	5	33	73	37	119	50	328	111	0.244	14.8	47.8	508	24282.4
C24xCol-	5	23	60	28	30	42	486	151	0.759	17.4	56.6	1047	59260.2
C24xCol+	5	51	142	55	31	69	547	182	1.069	13.6	47.2	1027	48474.4
C24xLer-	5	28	102	33	77	47	453	123	0.434	16.4	63.4	724	45901.6
C24xLer+	5	43	157	47	41	62	509	190	0.928	15	53.4	1086	57992.4
Col-/-	6	19	54	24	40	39	414	135	0.465	15.2	57.2	1116	63835.2
Col-/+	6	22	32	28	39	41	401	145	0.429	14.8	55.8	925	51615
Col+/-	6	21	35	26	45	39	357	129	0.45	13	48.4	869	42059.6
Col+/+	6	47	152	52	21	72	574	181	0.669	12.8	36	256	9216
Ler-/-	6	19	25	24	46	38	195	99	0.08	11	49	232	11368
Ler-/+C	6	21	31	27	17	42	202	140	0.219	11.4	47.6	632	30083.2
Ler-/+S	6	23	39	30	15	46	254	109	0.287	12.2	59.6	606	36117.6
Ler+/-	6	22	33	28	24	45	307	122	0.369	12.6	61	772	47092
Ler+/+	6	81	125	88	10	.	.	.	.	.	.	.	.
C24	6	35	76	40	168	53	396	141	0.394	14.6	43.6	810	35316
C24xCol-	6	28	102	33	73	43	469	139	0.73	16.4	58	1419	82302
C24xCol+	6	44	147	49	31	64	468	193	0.654	13.2	44.4	1001	44444.4
C24xLer-	6	28	62	33	52	47	503	148	0.596	17.4	64.8	1030	66744
C24xLer+	6	40	147	44	46	61	580	181	0.773	15.8	54	1077	58158

A period indicates that no data was available.

Table S6 Raw data for the number of seeds per silique and silique length measurements in the *FRI* and *FLC* experiments

Genotype FRI/FLC	Block	Seeds per Silique 1	Seeds per Silique 2	Seeds per Silique 3	Seeds per Silique 4	Seeds per Silique 5	Average # seeds / Silique	Silique Length 1	Silique Length 2	Silique Length 3	Silique Length 4	Silique Length 5	Average Silique Length
Col-/-	1	16	15	15	15	13	14.8	59	47	56	58	44	52.8
Col-/+	1	15	14	14	15	13	14.2	57	63	52	57	49	55.6
Col+/-	1	16	16	13	13	15	14.6	46	55	61	46	53	52.2
Col+/+	1	11	13	12	9	9	10.8	21	35	29	28	29	28.4
Ler-/-	1	11	11	9	11	10	10.4	44	56	43	47	42	46.4
Ler-/+C	1	13	13	13	13	12	12.8	51	54	53	56	58	54.4
Ler-/+S	1	11	12	13	12	12	12.0	50	54	50	53	64	54.2
Ler+/-	1	13	13	13	13	13	13.0	59	60	62	59	62	60.4
Ler+/+	1	8	11	11	7	.	9.25	23	41	36	25	.	31.25
C24	1	16	15	14	13	13	14.2	51	44	46	50	39	46.0
C24xCol-/-	1	18	18	18	17	17	17.6	58	61	62	64	67	62.4
C24xCol-/+	1	.	.	.	.	.	.	.	.	.	.	.	.
C24xLer-/-	1	19	19	17	16	16	17.4	63	62	60	57	52	58.8
C24xLer-/+	1	18	17	18	17	15	17.0	54	57	58	55	46	54.0
Col-/-	2	16	15	16	15	14	15.2	49	51	50	57	52	51.8
Col-/+	2	15	14	14	15	15	14.6	50	51	52	57	59	53.8
Col+/-	2	15	15	12	13	13	13.6	45	66	42	50	44	49.4
Col+/+	2	15	13	13	12	13	13.2	66	55	53	48	47	53.8
Ler-/-	2	11	11	11	11	12	11.2	52	49	53	54	64	54.4
Ler-/+C	2	12	11	11	11	11	11.2	44	52	51	46	53	49.2
Ler-/+S	2	11	12	13	12	11	11.8	46	50	55	65	58	54.8
Ler+/-	2	14	14	13	13	13	13.4	68	64	52	65	60	61.8
Ler+/+	2	.	.	.	.	.	.	.	.	.	.	.	.
C24	2	12	12	11	11	11	11.4	25	26	27	23	27	25.6
C24xCol-	2	16	17	16	16	15	16.0	56	50	56	54	62	55.6
C24xCol+	2	15	16	16	15	14	15.2	51	49	50	48	53	50.2
C24xLer-	2	17	16	15	15	15	15.6	67	55	54	58	53	57.4
C24xLer+	2	16	17	17	14	13	15.4	47	58	51	43	40	47.8

Col-/-	3	13	16	15	15	14	14.6	39	50	55	56	48	49.6
Col-/+	3	16	16	15	16	14	15.4	60	55	58	56	60	57.8
Col+/-	3	15	16	16	14	14	15.0	47	57	56	49	56	53.0
Col+/+	3	12	12	12	11	12	11.8	26	25	33	30	30	28.8
Ler-/-	3	12	11	11	9	10	10.6	52	52	41	50	41	47.2
Ler-/+C	3	13	12	13	11	12	12.2	44	54	58	50	49	51.0
Ler-/+S	3	12	11	12	11	11	11.4	67	57	63	51	50	57.6
Ler+/-	3	12	13	13	13	13	12.8	51	64	74	60	66	63.0
Ler+/+	3	11	10	11	11	10	10.6	42	43	58	55	51	49.8
C24	3	15	15	15	14	13	14.4	46	46	50	43	46	46.2
C24xCol-	3	15	19	18	16	16	16.8	47	62	64	59	54	57.2
C24xCol+	3	14	14	13	13	15	13.8	42	51	48	52	56	49.8
C24xLer-	3	16	15	15	15	14	15.0	57	50	58	55	47	53.4
C24xLer+	3	18	17	16	14	14	15.8	62	57	58	51	56	56.8
Col-/-	4	17	14	13	14	14	14.4	54	48	49	54	57	52.4
Col-/+	4	15	15	14	15	15	14.8	54	51	62	56	64	57.4
Col+/-	4	15	15	15	14	14	14.6	59	62	51	54	58	56.8
Col+/+	4	.	.	.	.	.	.	.	.	.	.	.	.
Ler-/-	4	11	11	11	10	11	10.8	43	41	46	48	47	45.0
Ler-/+C	4	12	12	11	11	10	11.2	47	50	42	47	48	46.8
Ler-/+S	4	11	9	10	10	11	10.2	45	39	40	51	51	45.2
Ler+/-	4	14	13	12	14	12	13.0	66	63	65	71	65	66.0
Ler+/+	4	12	12	10	11	11	11.2	53	53	56	49	52	52.6
C24	4	16	13	15	14	14	14.4	52	43	47	50	45	47.4
C24xCol-	4	19	19	20	18	17	18.6	58	59	64	59	73	62.6
C24xCol+	4	16	16	16	15	16	15.8	56	52	58	50	44	52.0
C24xLer-	4	16	16	15	15	15	15.4	59	65	55	62	59	60.0
C24xLer+	4	16	16	15	13	14	14.8	57	57	51	46	57	53.6
Col-/-	5	17	15	16	15	15	15.6	59	60	58	53	50	56.0
Col-/+	5	14	13	13	13	13	13.2	45	51	54	50	53	50.6
Col+/-	5	15	15	14	13	13	14.0	57	46	53	48	54	51.6
Col+/+	5	14	13	13	12	11	12.6	44	57	58	45	36	48.0
Ler-/-	5	12	10	10	9	9	10.0	49	40	48	42	40	43.8
Ler-/+C	5	12	12	12	12	10	11.6	57	46	57	53	49	52.4

Ler-/+S	5	12	12	13	12	10	11.8	54	57	55	56	50	54.4
Ler+/-	5	13	13	13	10	9	11.6	62	59	64	52	47	56.8
Ler+/+	5	12	12	11	11	11	11.4	43	51	44	44	50	46.4
C24	5	17	15	14	15	13	14.8	51	56	46	43	43	47.8
C24xCol-	5	19	19	17	17	15	17.4	53	66	50	63	51	56.6
C24xCol+	5	15	15	13	13	12	13.6	51	51	46	45	43	47.2
C24xLer-	5	18	17	17	15	15	16.4	66	62	67	64	58	63.4
C24xLer+	5	16	15	15	15	14	15.0	63	50	50	57	47	53.4
Col-/-	6	17	14	15	16	14	15.2	59	43	51	69	64	57.2
Col-/+	6	15	14	15	15	15	14.8	58	47	60	57	57	55.8
Col+/-	6	12	14	13	13	13	13.0	42	50	50	48	52	48.4
Col+/+	6	13	13	13	12	13	12.8	27	36	40	31	46	36.0
Ler-/-	6	11	11	11	12	10	11.0	48	41	55	58	43	49.0
Ler-/+C	6	12	12	11	11	11	11.4	49	51	47	48	43	47.6
Ler-/+S	6	13	11	13	12	12	12.2	63	53	61	61	60	59.6
Ler+/-	6	12	13	13	13	12	12.6	46	72	65	69	53	61.0
Ler+/+	6	.	.	.	.	.	.	.	.	.	.	.	.
C24	6	17	15	15	13	13	14.6	49	49	42	43	35	43.6
C24xCol-	6	16	17	17	16	16	16.4	55	60	64	53	58	58.0
C24xCol+	6	13	13	14	13	13	13.2	34	49	53	45	41	44.4
C24xLer-	6	18	18	17	17	17	17.4	64	63	63	68	66	64.8
C24xLer+	6	15	17	16	15	16	15.8	41	60	58	58	53	54.0

Silique length measurements are in millimeters.
