# Supplementary material for: An Evaluation of Arabidopsis thaliana Hybrid Traits and Their Genetic Control
Source: G3 (Bethesda). 2011 Dec 1;1(7):571–9. doi: 10.1534/g3.111.001156 (PMC3276180; doi:10.1534/g3.111.001156)
Supplement: Supporting Information [file supp_1.7.571_FigureS1.pdf]

A) Days to Bolting

MPH	C24	Col	Cvi	*Ler*	Ws
C24	-3.77	119.46	59.42	12.57	82.42
Col	103.24	16.46	-6.67	-2.56	-1.94
Cvi	59.42	-7.78	-2.97	-13.48	-11.86
Ler	18.03	-2.56	-13.48	0.00	-0.65
Ws	40.66	10.97	-14.12	-0.65	0.00

HPH	C24	Col	Cvi	*Ler*	Ws
C24		91.51	55.66	-2.83	56.60
Col	77.36		-16.83	-3.80	-3.80
Cvi	55.66	-17.82		-23.76	-22.77
Ler	1.89	-3.80	-23.76		-1.30
Ws	20.75	8.86	-24.75	-1.30	

B) Days to Flowering

MPH	C24	Col	Cvi	*Ler*	Ws
C24	-7.20	91.19	47.11	8.04	63.47
Col	77.97	8.82	-7.76	-4.48	-2.04
Cvi	48.76	-4.11	-0.85	-13.89	-4.27
Ler	13.39	-6.47	-8.33	-3.03	-0.52
Ws	31.51	10.20	-14.69	0.52	-3.19

HPH	C24	Col	Cvi	*Ler*	Ws
C24		73.60	42.40	-3.20	43.20
Col	61.60		-13.68	-5.88	-5.88
Cvi	44.00	-10.26		-20.51	-13.68
Ler	1.60	-7.84	-15.38		-3.03
Ws	15.20	5.88	-23.08	-2.02	

C) Days to Mature Seed

MPH	C24	Col	Cvi	*Ler*	Ws
C24	-4.37	64.22	33.90	8.24	45.07
Col	54.25	5.06	-5.78	-3.49	-2.80
Cvi	37.85	-3.95	1.17	-7.93	-5.88
Ler	8.82	0.95	-5.49	-1.27	-2.91
Ws	21.19	4.52	-10.84	-0.32	-2.63

HPH	C24	Col	Cvi	*Ler*	Ws
C24		53.01	29.51	0.55	32.79
Col	43.72		-9.36	-3.80	-4.64
Cvi	33.33	-7.60		-11.70	-11.11
Ler	1.09	0.63	-9.36		-4.46
Ws	10.93	2.53	-15.79	-1.91	

D) Rosette Diameter

MPH	C24	Col	Cvi	*Ler*	Ws
C24	-6.54	229.35	197.98	71.06	215.17
Col	234.55	38.60	-8.96	18.02	13.29
Cvi	202.92	2.49	-20.78	9.90	-22.17
Ler	93.28	8.72	4.46	-8.09	23.56
Ws	147.81	1.16	2.46	14.37	6.86

HPH	C24	Col	Cvi	*Ler*	Ws
C24		196.26	187.01	54.67	186.45
Col	200.93		-20.78	17.34	12.00
Cvi	191.77	-10.82		-3.90	-31.60
Ler	74.77	8.09	-8.66		22.86
Ws	125.23	0.00	-9.96	13.71	

E) Shoot Biomass

MPH	C24	Col	Cvi	*Ler*	Ws
C24	6.80	224.56	267.02	214.91	363.99
Col	204.29	7.80	-17.54	-33.79	-4.24
Cvi	312.73	29.23	-0.87	-33.78	-30.33
Ler	136.65	21.68	13.27	-6.58	51.45
Ws	423.02	39.45	9.00	-6.36	37.00

HPH	C24	Col	Cvi	*Ler*	Ws
C24		211.21	247.45	135.09	203.71
Col	191.77		-24.96	-51.91	-38.53
Cvi	290.73	17.59		-48.62	-53.11
Ler	76.66	-11.63	-12.11		23.20
Ws	242.35	-10.50	-26.64	-23.82	

F) Final Height

MPH	C24	Col	Cvi	Ler	Ws
C24	3.59	38.24	47.02	59.10	79.17
Col	36.91	4.13	2.62	-13.54	1.90
Cvi	45.05	3.11	-5.59	15.34	5.33
Ler	57.51	-2.37	28.25	-12.93	22.61
Ws	68.99	20.73	24.98	32.39	21.26

HPH	C24	Col	Cvi	Ler	Ws
C24		32.68	40.11	30.31	56.70
Col	31.40		-5.94	-31.40	-13.95
Cvi	38.24	-5.48		-1.71	-3.79
Ler	29.00	-22.54	9.29		13.52
Ws	47.79	1.95	14.16	22.57	

G) Total Number of Siliques

MPH	C24	Col	Cvi	Ler	Ws
C24	3.64	-5.59	85.49	78.86	73.99
Col	2.14	12.36	0.89	-29.56	10.57
Cvi	84.52	19.98	-12.68	-12.69	-4.79
Ler	62.69	1.56	27.06	-41.56	32.59
Ws	134.25	32.28	59.80	-21.27	15.03

HPH	C24	Col	Cvi	Ler	Ws
C24		-10.30	43.63	29.93	32.31
Col	-2.95		-24.64	-50.42	-18.79
Cvi	42.88	-10.38		-20.31	-7.19
Ler	18.19	-28.52	15.97		23.93
Ws	78.14	-2.85	55.76	-26.42	

H) Total Number of Seeds

MPH	C24	Col	Cvi	Ler	Ws
C24	-2.00	1.39	109.19	139.85	121.93
Col	13.18	8.57	-1.78	-12.61	7.46
Cvi	114.69	20.89	-17.76	-25.17	-15.11
Ler	132.09	13.12	51.65	-45.62	50.15
Ws	194.66	44.92	73.14	-0.43	14.88

HPH	C24	Col	Cvi	Ler	Ws
C24		-8.93	49.62	78.03	66.75
Col	1.66		-34.05	-39.45	-24.76
Cvi	53.56	-18.83		-29.35	-21.21
Ler	72.28	-21.61	43.19		47.44
Ws	121.39	1.47	60.70	-2.23	

I) Average Silique Length

MPH	C24	Col	Cvi	Ler	Ws
C24	0.16	-2.23	17.03	32.36	14.66
Col	2.64	-7.55	9.99	24.90	7.89
Cvi	19.29	10.72	-0.95	18.38	2.33
Ler	35.17	18.55	34.48	-4.51	31.68
Ws	28.32	19.82	4.66	27.17	5.33

HPH	C24	Col	Cvi	Ler	Ws
C24		-2.87	15.35	11.88	3.11
Col	1.97		7.72	6.14	-2.41
Cvi	17.58	8.43		-1.11	-9.15
Ler	14.25	0.75	12.33		22.77
Ws	15.40	8.38	-7.08	18.56	

J) Average Number of Seeds per Silique

MPH	C24	Col	Cvi	Ler	Ws
C24	-2.26	8.32	16.09	37.13	30.45
Col	11.89	-1.54	2.75	31.32	3.59
Cvi	20.71	6.28	-5.56	-5.11	-9.77
Ler	46.20	18.06	19.22	-5.48	19.07
Ws	29.14	13.74	5.24	31.69	0.53

HPH	C24	Col	Cvi	Ler	Ws
C24		1.72	5.30	36.40	27.87
Col	5.07		-11.90	23.94	-4.52
Cvi	9.49	-8.87		-14.34	-16.64
Ler	45.42	11.42	7.62		16.11
Ws	26.59	4.84	-2.78	28.41	

K) Height at Flowering

MPH	C24	Col	Cvi	Ler	Ws
C24	-12.79	-61.18	-31.91	16.05	-18.69
Col	-45.65	4.74	-8.62	65.97	-0.90
Cvi	2.38	21.03	0.98	43.84	-2.05
Ler	77.78	75.76	46.92	43.75	93.82
Ws	-17.65	43.86	-22.25	91.27	7.22

HPH	C24	Col	Cvi	Ler	Ws
C24		-68.03	-33.33	-15.86	-20.46
Col	-55.24		-25.98	40.71	-16.93
Cvi	0.25	-1.96		2.94	-6.13
Ler	28.90	49.01	5.15		42.51
Ws	-19.44	20.59	-25.49	40.64	

LPH	C24	Col	Cvi	Ler	Ws
C24		-50.59	-30.43	86.93	-16.84
Col	-30.83		19.37	102.27	22.79
Cvi	4.60	58.10		138.64	2.41
Ler	186.36	114.20	143.75		202.84
Ws	-15.78	78.26	-18.72	198.86	

L) Lifespan

MPH	C24	Col	Cvi	Ler	Ws
C24	-0.20	-11.73	1.90	0.76	8.77
Col	-10.01	-2.60	2.31	11.34	8.56
Cvi	-0.76	-8.42	-7.92	-4.70	-6.06
Ler	9.41	7.56	8.05	2.42	27.53
Ws	6.26	10.86	4.24	0.81	15.66

HPH	C24	Col	Cvi	Ler	Ws
C24		-13.94	-1.10	-8.81	1.96
Col	-12.27		1.84	-1.49	-0.62
Cvi	-3.68	-8.84		-16.02	-14.36
Ler	-0.98	-4.83	-4.79		22.82
Ws	-0.39	1.49	-4.97	-2.91	

FIGURE S1 The percent MPH and HPH for all hybrids within the diallel for all 12 traits Within each matrix, the value in the cell indicates the percent difference from: the midparent value for mid-parent heterosis (MPH); the high-parent value for high-parent heterosis (HPH); and the low-parent value for low-parent heterosis (LPH) . LPH is only shown for height at flowering because no other trait had significant low-parent heterosis. The color of each cell indicates the significance level of the difference. The maternal genotype is on the vertical axis and the paternal genotype is on the horizontal axis. All 12 traits are shown for comparison purposes, though four are also included in main text.
A - days to bolting, - **B** days to flowering, **C** - days to mature seed, **D** - rosette diameter, **E** - shoot biomass, **F** - final height, **G** - total number of siliques, **H** - total number of seeds, **I** - silique length, **J** - average number of seeds per silique, **K** - height at flowering, **L** – lifespan.

Hybrid Larger	Hybrid Smaller
P < 0.0001	P < 0.0001
0.0001 < P < 0.001	0.0001 < P < 0.001
0.001 < P < 0.01	0.001 < P < 0.01
0.01 < P < 0.05	0.01 < P < 0.05
0.05 < P < 0.1	0.05 < P < 0.1
