# Supplementary material for: An Evaluation of Arabidopsis thaliana Hybrid Traits and Their Genetic Control
Source: G3 (Bethesda). 2011 Dec 1;1(7):571–9. doi: 10.1534/g3.111.001156 (PMC3276180; doi:10.1534/g3.111.001156)
Supplement: Supporting Information [file supp_1.7.571_FigureS2.pdf]

A

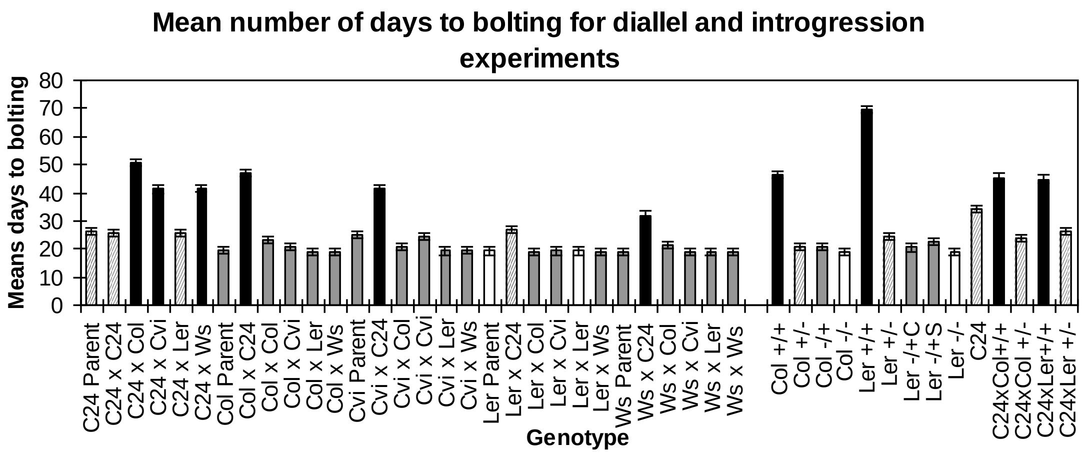
Mean number of days to bolting for diallel and introgression experiments
Means days to bolting
0
10
20
30
40
50
60
70
80
C24 Parent
C24 x C24
C24 x Col
C24 x Cvi
C24 x Ler
C24 x Ws
Col Parent
Col x C24
Col x Col
Col x Cvi
Col x Ler
Col x Ws
Cvi Parent
Cvi x C24
Cvi x Col
Cvi x Cvi
Cvi x Ler
Cvi x Ws
Ler Parent
Ler x C24
Ler x Col
Ler x Cvi
Ler x Ler
Ler x Ws
Ws Parent
Ws x C24
Ws x Col
Ws x Cvi
Ws x Ler
Ws x Ws
Col +/+
Col +/-
Col -/+
Col -/-
Ler +/+
Ler +/-
Ler -/+C
Ler -/+S
Ler -/-
C24
C24xCol+/+
C24xCol +/-
C24xLer+/+
C24xLer +/-
Genotype

B

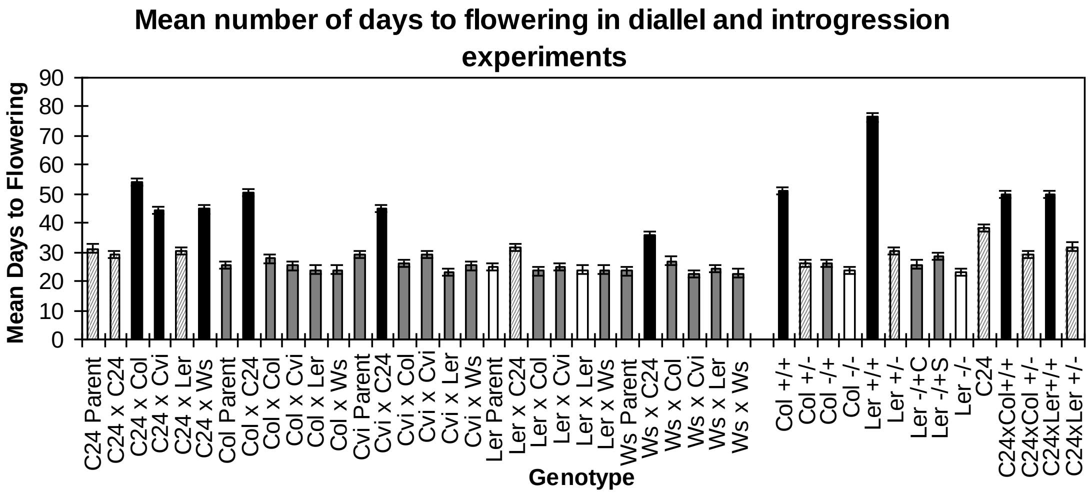
Mean number of days to flowering in diallel and introgression experiments
Mean Days to Flowering
0
10
20
30
40
50
60
70
80
90
C24 Parent
C24 x C24
C24 x Col
C24 x Cvi
C24 x Ler
C24 x Ws
Col Parent
Col x C24
Col x Col
Col x Cvi
Col x Ler
Col x Ws
Cvi Parent
Cvi x C24
Cvi x Col
Cvi x Cvi
Cvi x Ler
Cvi x Ws
Ler Parent
Ler x C24
Ler x Col
Ler x Cvi
Ler x Ler
Ler x Ws
Ws Parent
Ws x C24
Ws x Col
Ws x Cvi
Ws x Ler
Ws x Ws
Col +/+
Col +/-
Col -/+
Col -/-
Ler +/+
Ler +/-
Ler -/+C
Ler -/+S
Ler -/-
C24
C24xCol+/+
C24xCol +/-
C24xLer+/+
C24xLer +/-
Genotype

C

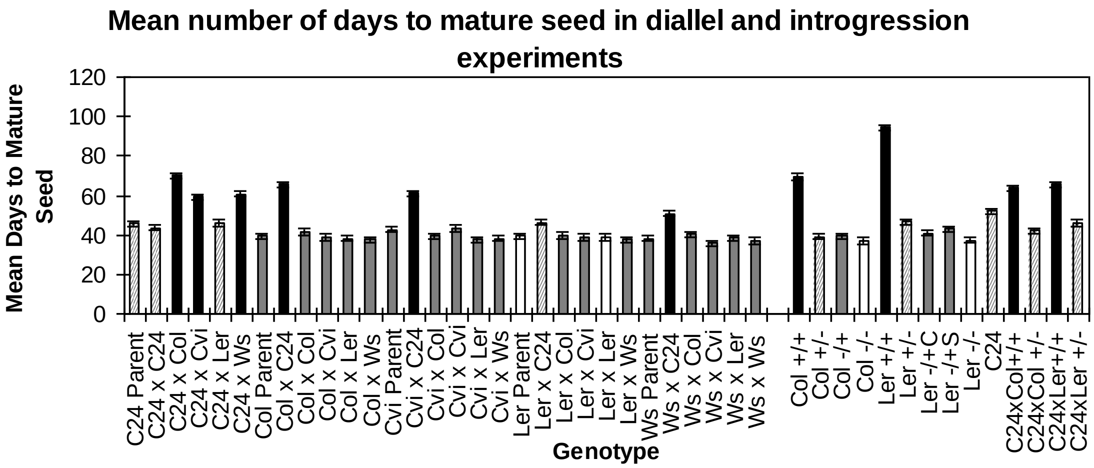
Mean number of days to mature seed in diallel and introgression experiments
Mean Days to Mature Seed
0
20
40
60
80
100
120
C24 Parent
C24 x C24
C24 x Col
C24 x Cvi
C24 x Ler
C24 x Ws
Col Parent
Col x C24
Col x Col
Col x Cvi
Col x Ler
Col x Ws
Cvi Parent
Cvi x C24
Cvi x Col
Cvi x Cvi
Cvi x Ler
Cvi x Ws
Ler Parent
Ler x C24
Ler x Col
Ler x Cvi
Ler x Ler
Ler x Ws
Ws Parent
Ws x C24
Ws x Col
Ws x Cvi
Ws x Ler
Ws x Ws
Col +/+
Col +/-
Col -/+
Col -/-
Ler +/+
Ler +/-
Ler -/+C
Ler -/+S
Ler -/-
C24
C24xCol+/+
C24xCol +/-
C24xLer+/+
C24xLer +/-
Genotype

p[

D

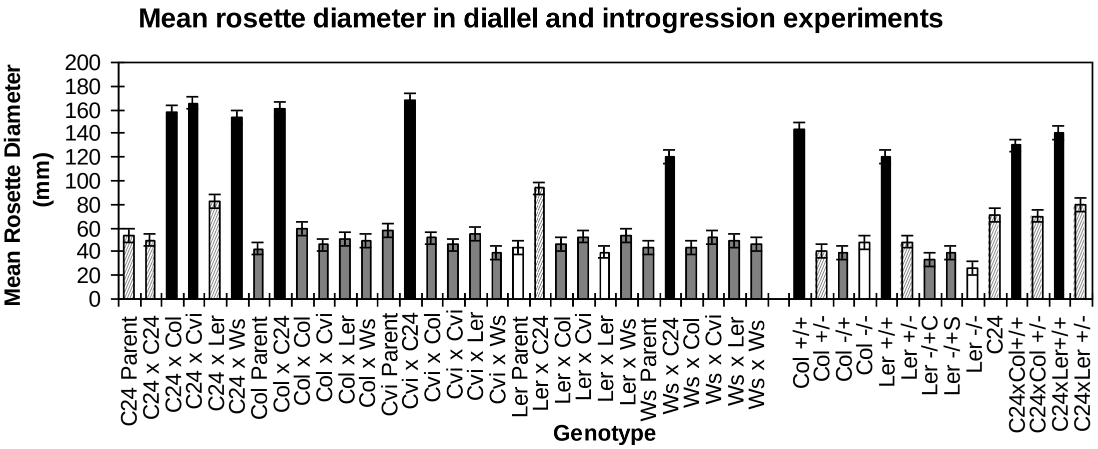
Mean rosette diameter in diallel and introgression experiments
Mean Rosette Diameter (mm)
0
20
40
60
80
100
120
140
160
180
200
C24 Parent
C24 x C24
C24 x Col
C24 x Cvi
C24 x Ler
C24 x Ws
Col Parent
Col x C24
Col x Col
Col x Cvi
Col x Ler
Col x Ws
Cvi Parent
Cvi x C24
Cvi x Col
Cvi x Cvi
Cvi x Ler
Cvi x Ws
Ler Parent
Ler x C24
Ler x Col
Ler x Cvi
Ler x Ler
Ler x Ws
Ws Parent
Ws x C24
Ws x Col
Ws x Cvi
Ws x Ler
Ws x Ws
Col +/+
Col +/-
Col -/+
Col -/-
Ler +/+
Ler +/-
Ler -/+C
Ler -/+S
Ler -/-
C24
C24xCol+/+
C24xCol +/-
C24xLer+/+
C24xLer +/-
Genotype

E

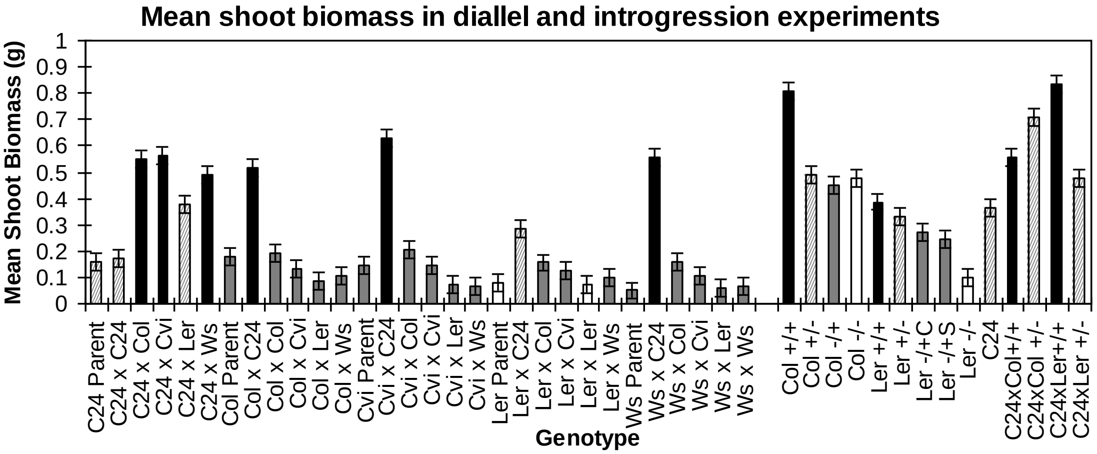
Mean shoot biomass in diallel and introgression experiments
Mean Shoot Biomass (g)
0
0.1
0.2
0.3
0.4
0.5
0.6
0.7
0.8
0.9
1
C24 Parent
C24 x C24
C24 x Col
C24 x Cvi
C24 x Ler
C24 x Ws
Col Parent
Col x C24
Col x Col
Col x Cvi
Col x Ler
Col x Ws
Cvi Parent
Cvi x C24
Cvi x Col
Cvi x Cvi
Cvi x Ler
Cvi x Ws
Ler Parent
Ler x C24
Ler x Col
Ler x Cvi
Ler x Ler
Ler x Ws
Ws Parent
Ws x C24
Ws x Col
Ws x Cvi
Ws x Ler
Ws x Ws
Col +/+
Col +/-
Col -/+
Col -/-
Ler +/+
Ler +/-
Ler -/+C
Ler -/+S
Ler -/-
C24
C24xCol+/+
C24xCol +/-
C24xLer+/+
C24xLer +/-
Genotype

F

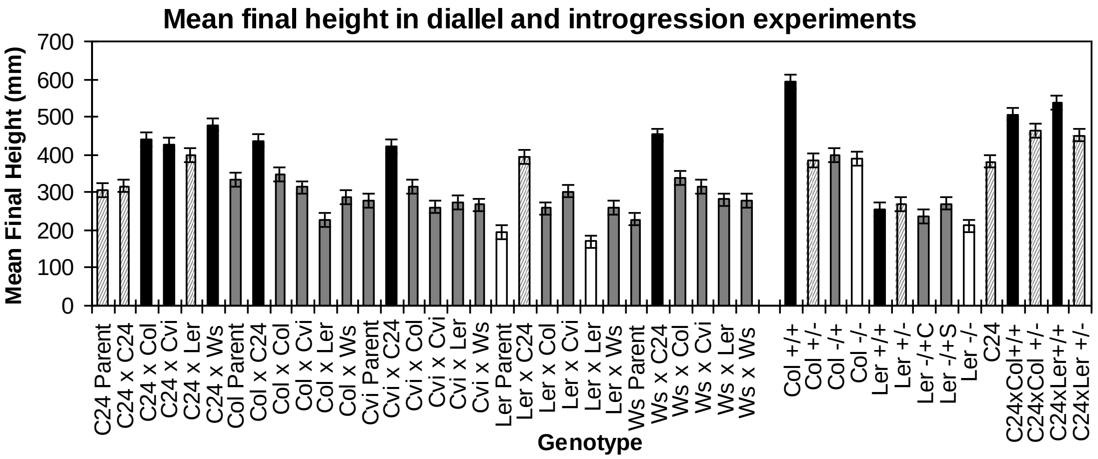
Mean final height in diallel and introgression experiments
Mean Final Height (mm)
0
100
200
300
400
500
600
700
C24 Parent
C24 x C24
C24 x Col
C24 x Cvi
C24 x Ler
C24 x Ws
Col Parent
Col x C24
Col x Col
Col x Cvi
Col x Ler
Col x Ws
Cvi Parent
Cvi x C24
Cvi x Col
Cvi x Cvi
Cvi x Ler
Cvi x Ws
Ler Parent
Ler x C24
Ler x Col
Ler x Cvi
Ler x Ler
Ler x Ws
Ws Parent
Ws x C24
Ws x Col
Ws x Cvi
Ws x Ler
Ws x Ws
Col +/+
Col +/-
Col -/+
Col -/-
Ler +/+
Ler +/-
Ler -/+C
Ler -/+S
Ler -/-
C24
C24xCol+/+
C24xCol +/-
C24xLer+/+
C24xLer +/-
Genotype

G

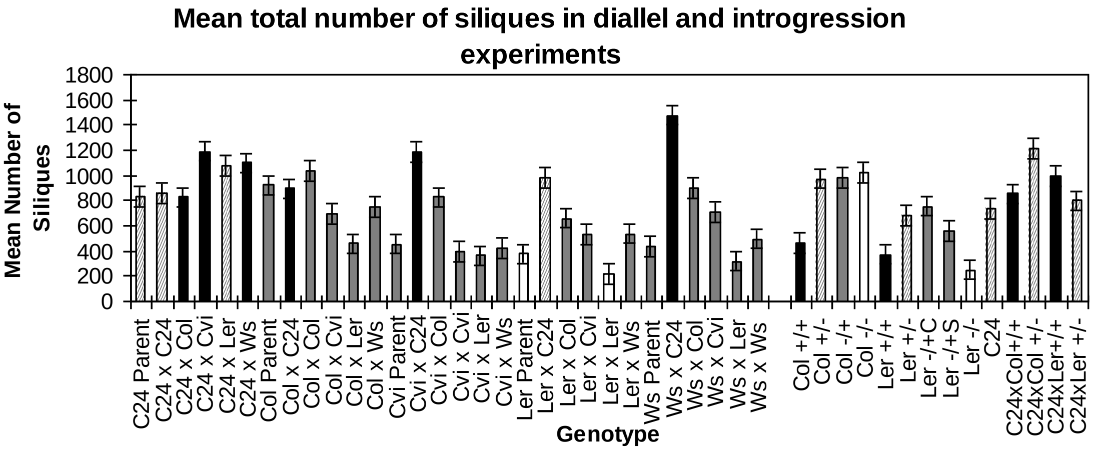
Mean total number of siliques in diallel and introgression experiments
Mean Number of Siliques
0
200
400
600
800
1000
1200
1400
1600
1800
C24 Parent
C24 x C24
C24 x Col
C24 x Cvi
C24 x Ler
C24 x Ws
Col Parent
Col x C24
Col x Col
Col x Cvi
Col x Ler
Col x Ws
Cvi Parent
Cvi x C24
Cvi x Col
Cvi x Cvi
Cvi x Ler
Cvi x Ws
Ler Parent
Ler x C24
Ler x Col
Ler x Cvi
Ler x Ler
Ler x Ws
Ws Parent
Ws x C24
Ws x Col
Ws x Cvi
Ws x Ler
Ws x Ws
Col +/+
Col +/-
Col -/+
Col -/-
Ler +/+
Ler +/-
Ler -/+C
Ler -/+S
Ler -/-
C24
C24xCol+/+
C24xCol +/-
C24xLer+/+
C24xLer +/-
Genotype

H

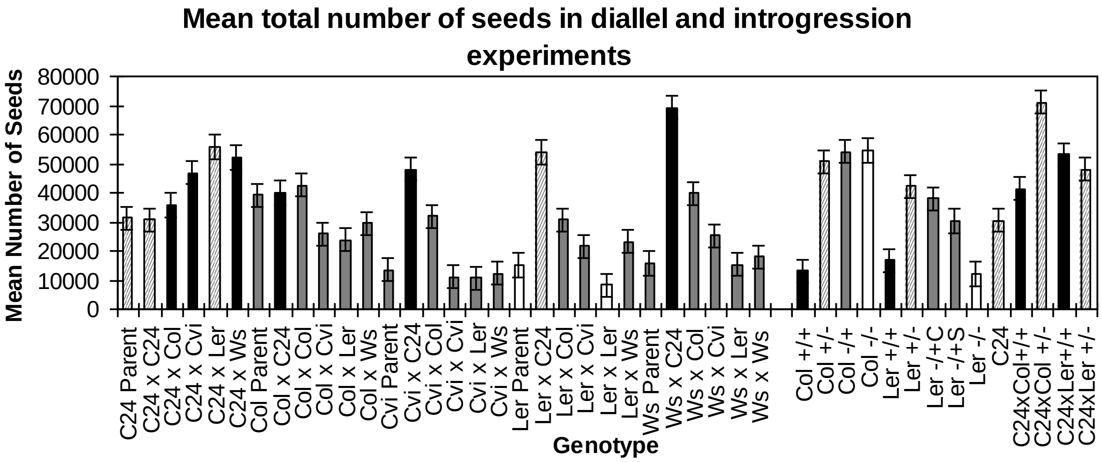
Mean total number of seeds in diallel and introgression experiments
Mean Number of Seeds
0
10000
20000
30000
40000
50000
60000
70000
80000
C24 Parent
C24 x C24
C24 x Col
C24 x Cvi
C24 x Ler
C24 x Ws
Col Parent
Col x C24
Col x Col
Col x Cvi
Col x Ler
Col x Ws
Cvi Parent
Cvi x C24
Cvi x Col
Cvi x Cvi
Cvi x Ler
Cvi x Ws
Ler Parent
Ler x C24
Ler x Col
Ler x Cvi
Ler x Ler
Ler x Ws
Ws Parent
Ws x C24
Ws x Col
Ws x Cvi
Ws x Ler
Ws x Ws
Col +/+
Col +/-
Col -/+
Col -/-
Ler +/+
Ler +/-
Ler -/+C
Ler -/+S
Ler -/-
C24
C24xCol+/+
C24xCol +/-
C24xLer+/+
C24xLer +/-
Genotype

I

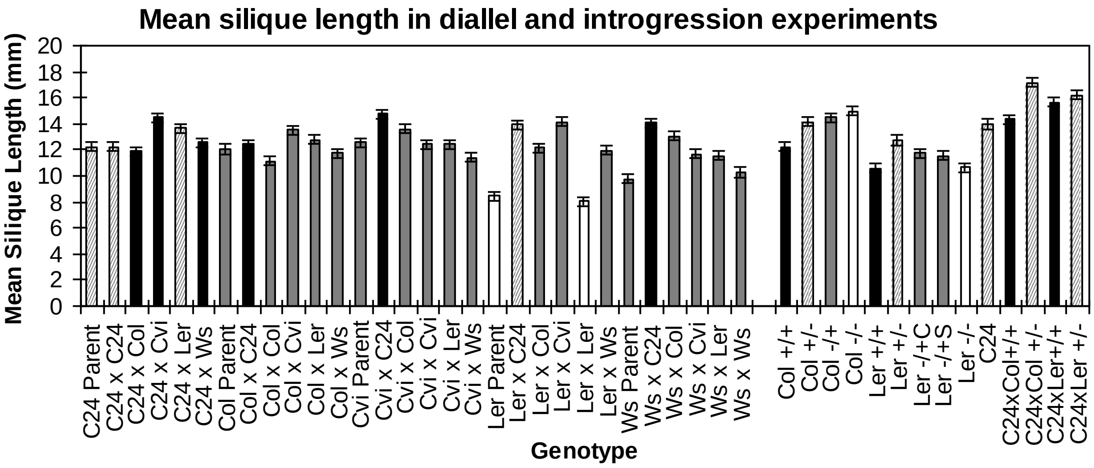
Mean silique length in diallel and introgression experiments
Mean Silique Length (mm)
0
2
4
6
8
10
12
14
16
18
20
C24 Parent
C24 x C24
C24 x Col
C24 x Cvi
C24 x Ler
C24 x Ws
Col Parent
Col x C24
Col x Col
Col x Cvi
Col x Ler
Col x Ws
Cvi Parent
Cvi x C24
Cvi x Col
Cvi x Cvi
Cvi x Ler
Cvi x Ws
Ler Parent
Ler x C24
Ler x Col
Ler x Cvi
Ler x Ler
Ler x Ws
Ws Parent
Ws x C24
Ws x Col
Ws x Cvi
Ws x Ler
Ws x Ws
Col +/+
Col +/-
Col -/+
Col -/-
Ler +/+
Ler +/-
Ler -/+C
Ler -/+S
Ler -/-
C24
C24xCol+/+
C24xCol +/-
C24xLer+/+
C24xLer +/-
Genotype

J

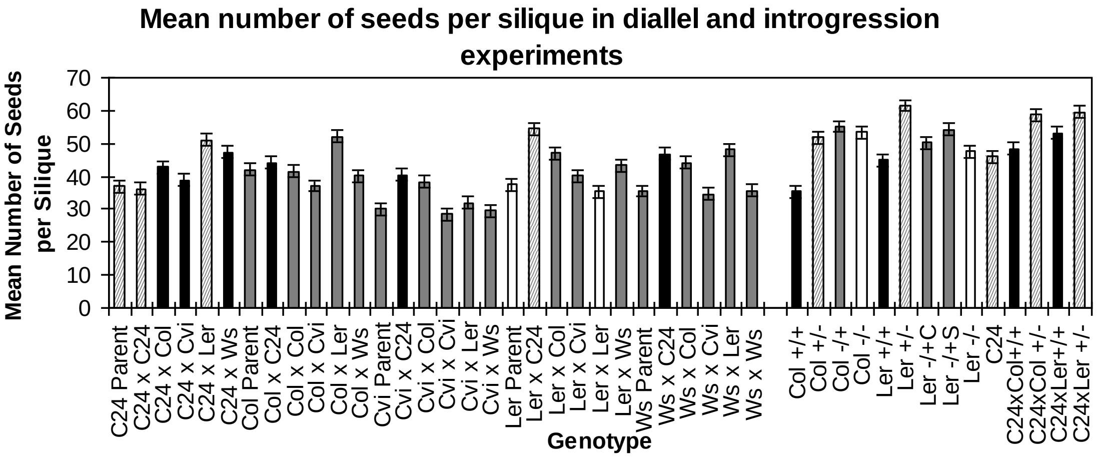
Mean number of seeds per silique in diallel and introgression experiments
Mean Number of Seeds per Silique
Genotype
C24 Parent
C24 x C24
C24 x Col
C24 x Cvi
C24 x Ler
C24 x Ws
Col Parent
Col x C24
Col x Col
Col x Cvi
Col x Ler
Col x Ws
Cvi Parent
Cvi x C24
Cvi x Col
Cvi x Cvi
Cvi x Ler
Cvi x Ws
Ler Parent
Ler x C24
Ler x Col
Ler x Cvi
Ler x Ler
Ler x Ws
Ws Parent
Ws x C24
Ws x Col
Ws x Cvi
Ws x Ler
Ws x Ws
Col +/+
Col +/-
Col -/+
Col -/-
Ler +/+
Ler +/-
Ler -/+C
Ler -/+S
Ler -/-
C24
C24xCol+/+
C24xCol +/-
C24xLer+/+
C24xLer +/-

K

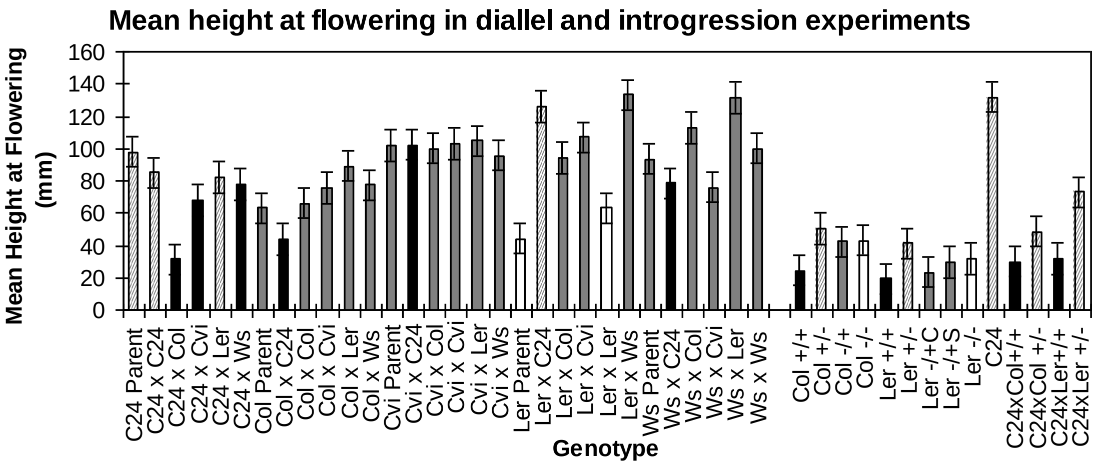
Mean height at flowering in diallel and introgression experiments
Mean Height at Flowering (mm)
Genotype

L

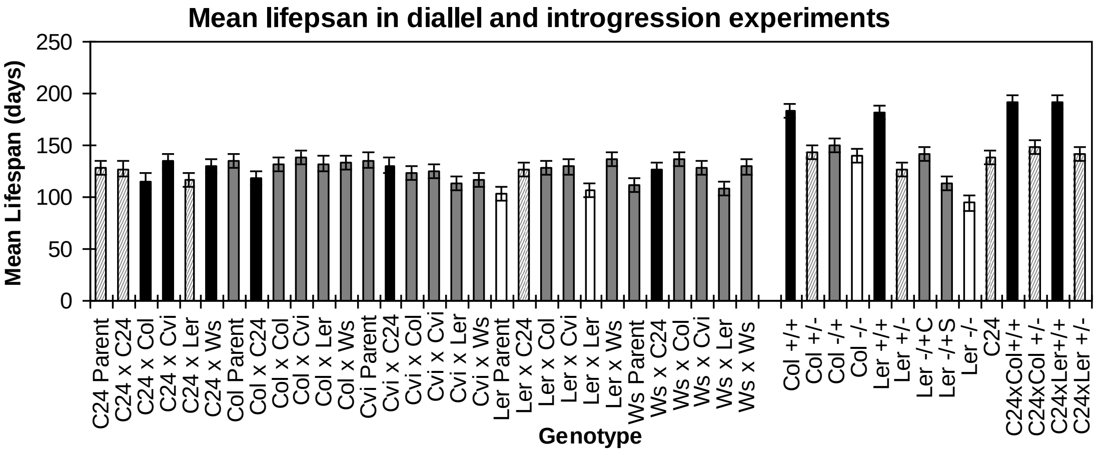
Mean lifepsan in diallel and introgression experiments
Mean Lifespan (days)
Genotype

Figure S2 Mean trait values for all genotypes in the diallel and introgression experiments. The means of all 12 traits (A-L) in the diallel and *FRI* and *FLC* introgression line experiments were plotted on the same chart to compare means across experiments. The color of the bar corresponds to the *FRI* and *FLC* status of the genotype: black have functional *FRI* and strong *FLC*; striped have functional *FRI* only; grey have functional *FLC* only; and white have non-functional alleles at both *FRI* and *FLC*. The means for the diallel span the density treatments since density had little effect on traits. The bars are the standard error of the mean estimates. **A -** days to bolting, - **B** days to flowering, **C** - days to mature seed, **D** - rosette diameter, **E** - shoot biomass, **F** - final height, **G** - total number of siliques, **H** - total number of seeds, **I** - silique length, **J** - average number of seeds per silique, **K** - height at flowering, **L** – lifespan.
