# Supplementary material for: An Evaluation of Arabidopsis thaliana Hybrid Traits and Their Genetic Control
Source: G3 (Bethesda). 2011 Dec 1;1(7):571–9. doi: 10.1534/g3.111.001156 (PMC3276180; doi:10.1534/g3.111.001156)
Supplement: Supporting Information [file supp_1.7.571_FigureS3.pdf]

A

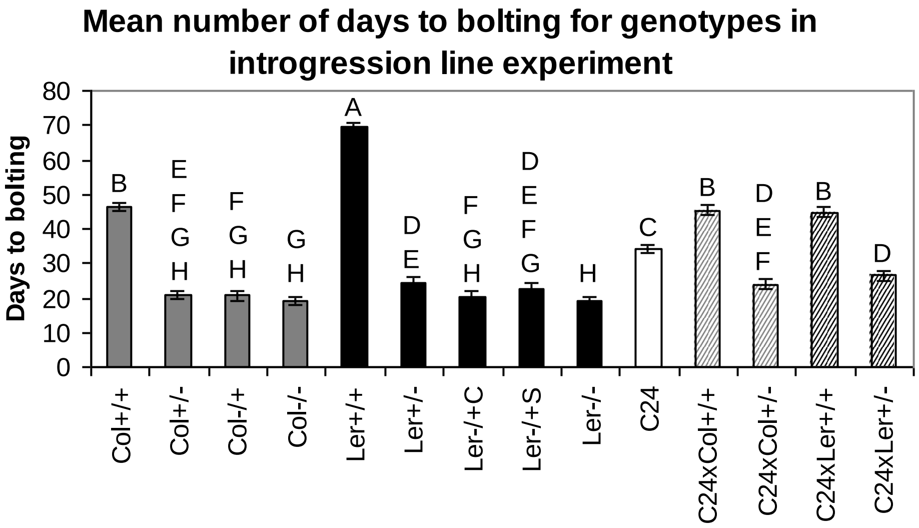

Mean number of days to bolting for genotypes in introgression line experiment
Days to bolting
0
10
20
30
40
50
60
70
80
B
E F G H
F G H
G H
A
D E
F G H
D E F G
H
C
B
D E F
B
D
Col+/+
Col+/-
Col-/+
Col-/-
Ler+/+
Ler+/-
Ler-/+C
Ler-/+S
Ler-/-
C24
C24xCol+/+
C24xCol+/-
C24xLer+/+
C24xLer+/-

B

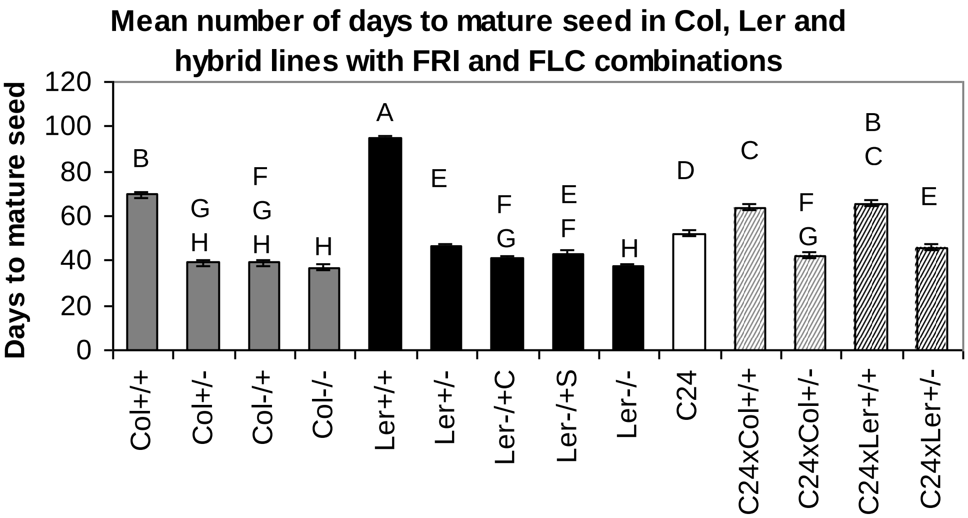

Mean number of days to mature seed in Col, Ler and hybrid lines with FRI and FLC combinations
Days to mature seed
0
20
40
60
80
100
120
B
G H
F G H
H
A
E
F G
E F
H
D
C
F G
B C
E
Col+/+
Col+/-
Col-/+
Col-/-
Ler+/+
Ler+/-
Ler-/+C
Ler-/+S
Ler-/-
C24
C24xCol+/+
C24xCol+/-
C24xLer+/+
C24xLer+/-

C

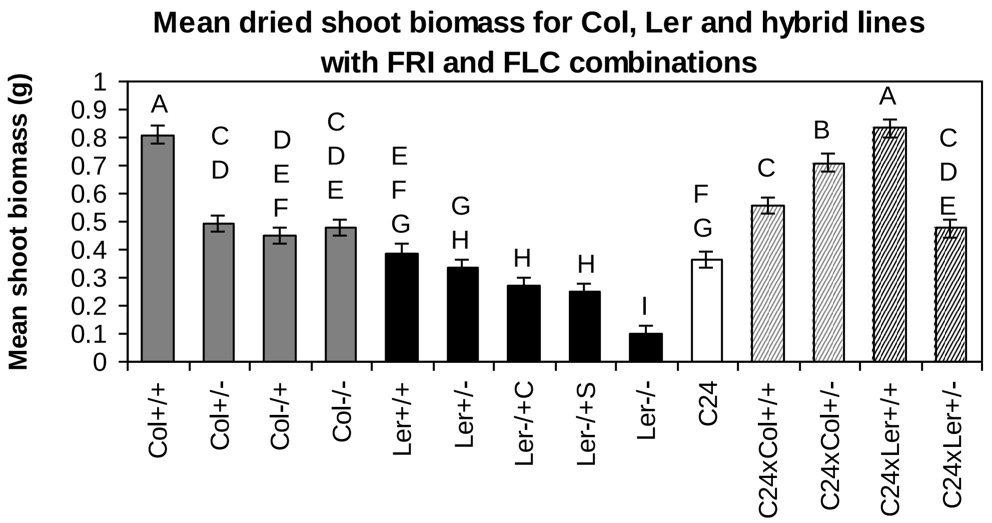
Mean dried shoot biomass for Col, Ler and hybrid lines with FRI and FLC combinations
Mean shoot biomass (g)
1
0.9
0.8
0.7
0.6
0.5
0.4
0.3
0.2
0.1
0
A
C D
D E F
C D E
E F G
G H
H
H
I
F G
C
B
A
C D E
Col+/+
Col+/-
Col-/+
Col-/-
Ler+/+
Ler+/-
Ler-/+C
Ler-/+S
Ler-/-
C24
C24xCol+/+
C24xCol+/-
C24xLer+/+
C24xLer+/-

D

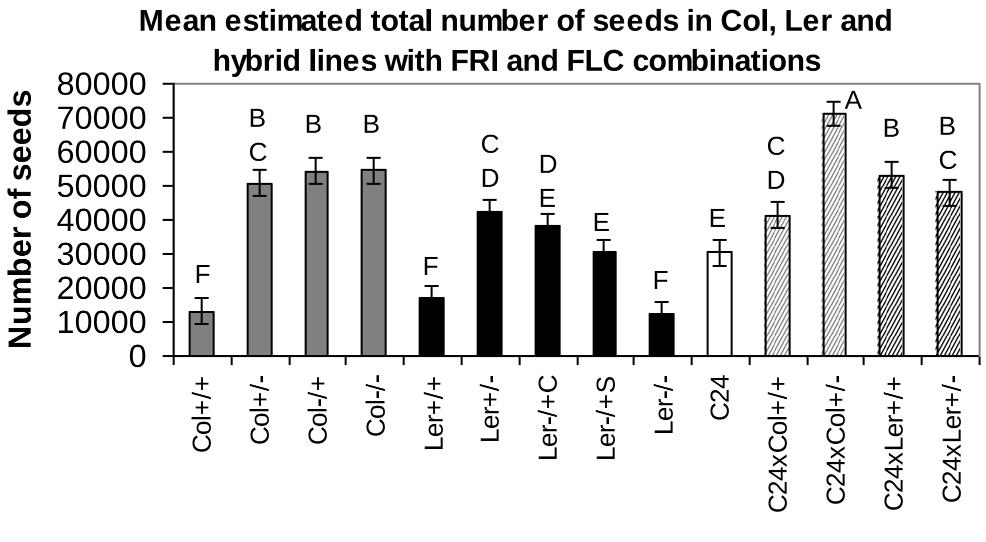
Mean estimated total number of seeds in Col, Ler and hybrid lines with FRI and FLC combinations
Number of seeds
80000
70000
60000
50000
40000
30000
20000
10000
0
F
B C
B
B
F
C D
D E
E
F
E
C D
A
B
B C
Col+/+
Col+/-
Col-/+
Col-/-
Ler+/+
Ler+/-
Ler-/+C
Ler-/+S
Ler-/-
C24
C24xCol+/+
C24xCol+/-
C24xLer+/+
C24xLer+/-

E

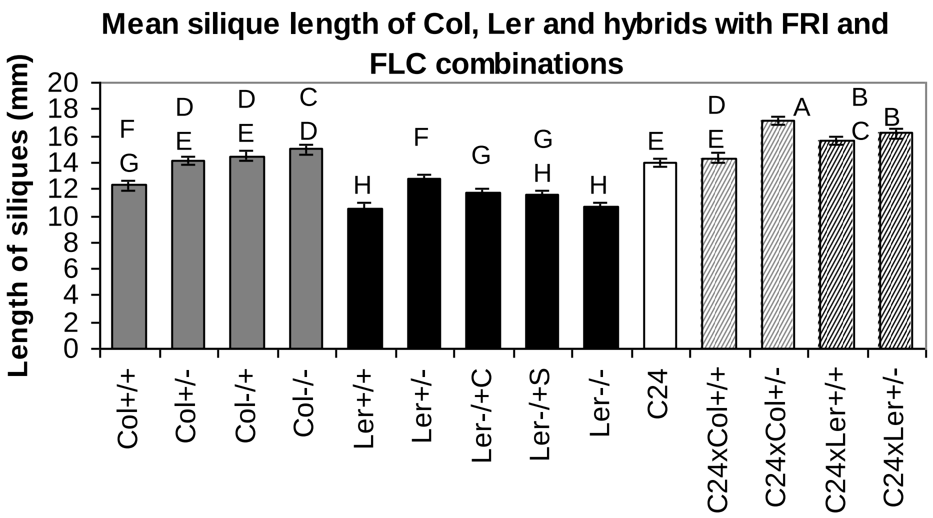

F

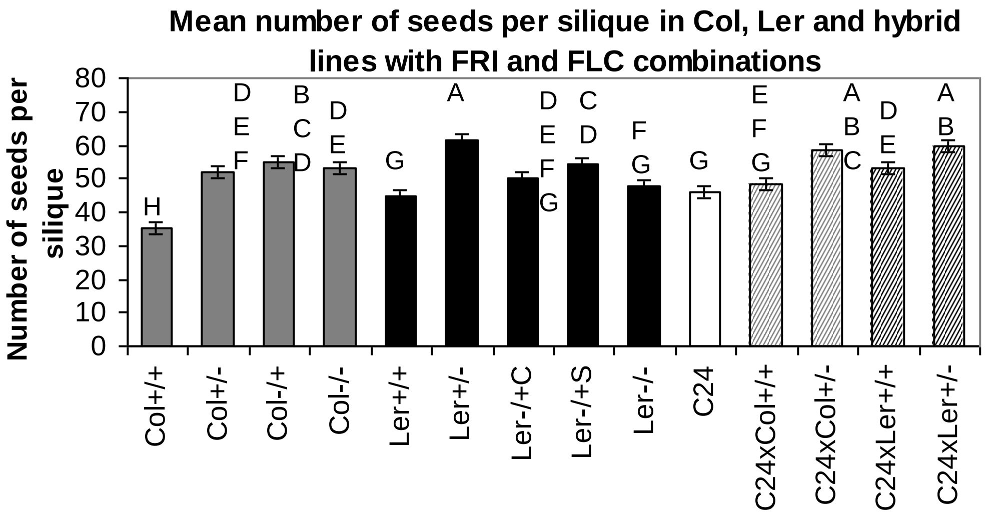

Figure S3 Means of the fourteen introgression experiment genotypes for the 6 traits not included in main text. The mean values of Col, L*er*, C24 x Col and C24 x L*er* lines with various *FRI* and *FLC* allele combinations. Functional or strong alleles are indicated by "+", while non-functional or weak alleles are indicated by "-". The status of the *FRI* allele is listed before the "/" and the status of *FLC* listed after. The color of the bars indicates the genotypic background: solid grey bars are Col inbred lines, black bars are L*er* inbred lines, white bars are C24 lines, striped grey and white bars are hybrids between Col and C24, and striped black and white bars are hybrids between L*er* and C24. Bars with different letters are significantly different at $P < 0.05$.
A: Mean number of days to bolting.
B: Mean number of days to mature seed.
C: Mean shoot biomass.
D: Mean estimated total number of seeds.
E: Mean silique length for all 14 lines.
F: Mean number of seeds per silique.
