# Supplementary material for: An Evaluation of Arabidopsis thaliana Hybrid Traits and Their Genetic Control
Source: G3 (Bethesda). 2011 Dec 1;1(7):571–9. doi: 10.1534/g3.111.001156 (PMC3276180; doi:10.1534/g3.111.001156)
Supplement: Supporting Information [file supp_1.7.571_FileS1.pdf]

File S1

Supporting Materials and Methods

Plant growth and trait measurements

All seeds were sterilized and incubated at 4º for four days then placed on germination media. Seedlings were germinated under continuous light at room temperature. After two weeks the seedlings were moved to 3.5" x 3.5" pots with LA-4 Sunshine Mix (Sun Gro) with one pellet of 14-14-14 fertilizer and placed in a growth chamber (Conviron). Light intensity from fluorescent bulbs was measured at the shelf level. Plants were watered with deionized tap water approximately every three days or as needed.

Days to bolting was the time between germination and when a visible bud was formed. Days to flowering was the time between germination and when a flower fully opened. Days to mature seed was time until a silique was fully browned and opened to release mature seeds. Height at flowering was measured from the base of the stem to the top of the plant. For silique number, all mature siliques were collected and counted for each plant throughout development. (In the diallel, 87,306 siliques were counted, and in the introgression experiment 61,818 siliques were counted.) The silique length was averaged from 25 siliques per plant in the diallel and five siliques per plant in the introgression experiment. The number of seeds within a silique was averaged from 20 siliques per plant in the diallel and five siliques per plant in the introgression experiment. (In the diallel, 97,410 seeds were counted, and in the introgression experiment, 20,710 seeds were counted.) The final plant height was measured from the base of the stem to the top of the tallest branch at death. The lifespan was considered the time from sowing on germination media until death. Shoot biomass was determined by drying the stem (with no rosette leaves) at 80º for 48 hours then weighing it. The total number of seeds was estimated by multiplying the total number of siliques and the average number of seeds per silique for each plant. Plants were checked daily.

Seed source

The five *A. thaliana* parental lines, Columbia (Col), Wassilewskija (Ws), Landsberg *erecta* (L*er*), Cape Verde Islands (Cvi), and C24, were obtained from Dr. Stephen Chatfield and originally ordered from Lehle Seeds (catalogue numbers WT-2, WT-8A, WT-4, WT-18, and WT-23, respectively). Manual self pollinations of parent lines were made to control for differences in seeds produced by selfing versus manual crossing (Meyer *et al*. 2004).

We obtained Col and Ler genotypes into which a number of FRI and FLC alleles had been introgressed. Non-functional *FLC* alleles were produced with fast neutron radiation mutagenesis (*flc*-3) (Col -/-), while the functional *FRI* allele was introgressed into wild-type Col -/+ and Col -/- from the Sf2 accession (producing Col +/+ and Col +/-, respectively) (Lee and Amasino 1995; Michaels and Amasino 1999). In the L*er* background (wild-type L*er* -/-), functional alleles of *FRI* and *FLC* were introgressed from the Sf2 accession (producing L*er* -/+, L*er* +/-, and L*er* +/+) (Lee *et al*. 1994). An additional line was used with a functional *FLC* allele from Col introgressed into L*er* (L*er* -/+ *FLC*-Col) (Koornneef *et al*. 1994). Wild-type seeds were obtained from Dr. Stephen Chatfield, all other seeds were obtained from Dr. Richard Amasino, except for L*er* -/+ *FLC*-Col, which was ordered from The Arabidopsis Biological Research Center (ABRC) (Alonso et al. 2003). The introgressed segment is referred to using the name of introgressed gene of interest (i.e. *FRI* or *FLC*) (Lee *et al*. 1993; Koornneef *et al*. 1994; Lee *et al*. 1994; Michaels and Amasino 1999; Michaels and Amasino 2001).

C24 hybrids were generated by crossing the following lines: C24 +/- with Col -/+; C24 +/- with L*er* -/+ (*FLC*-Col); C24 +/- with Col -/-; and C24 +/- with L*er* -/-, respectively

Statistical analyses

DIALLEL-SAS05, the program that executed Griffing's diallel analysis, required balanced data to compute diallel statistics. Therefore, the trait values for the three plants with incorrect genotypes (as determined by CAPS marker analysis) were substituted with pseudoreplicates - estimated trait values calculated from other plants of the correct genotype.

We used the following model for the split plot analysis of diallel data

$$Y_{ijk} = \mu + \alpha_i + \eta_{k(i)} + \beta_j + \alpha\beta_{ij} + \varepsilon_{k(ij)}$$

where α_i is the whole plot factor (density); $\eta_{k(i)}$ is the replication nested within the density; β_j is the subplot factor (genotype); $\alpha\beta_{ij}$ is the interaction between genotype and density; and $\varepsilon_{k(ij)}$ is the residual error.

Among the introgression lines and their hybrids, we used the following model:

$$Y_{ij} = \mu + \alpha_i + \beta_j + \varepsilon_{ij}$$

where α_i is the genotype (treatment) effect, β_j is the block effect, and ε_{ij} is the residual error.

Among inbreds, five sets of contrasts were performed to determine the effects of *FRI* and *FLC*. (1) To evaluate the effect of *FRI* in a background with a non-functional *FLC* allele, the +/- line was compared to the -/- line. (2) The effect of *FRI* in a background with a functional *FLC* was determined by contrasting the -/+ and +/+ lines. (3) The effect of *FLC* with a non-functional *FRI* was determined by contrasting the -/- and -/+ lines. (4) The effect of *FLC* when a functional *FRI* is present was determined by contrasting +/- and +/+ lines. Finally, (5) the effect of both genes together was determined by comparing the -/- and +/+ varieties.

References

Alonso, J. et al., 2003. Genome-wide insertional mutagenesis of *Arabidopsis thaliana*. Science 301: 653-657.

Koornneef, M., H. B. Vries, C. Hanhart, W. Soppe and T. Peeters, 1994 The phenotype of some late-flowering mutants is enhanced by a locus on chromosome 5 that is not effective in the Landsberg *erecta* wild-type. Plant J. **6:** 911-919.

Lee, I., A. Bleecker and R. Amasino, 1993 Analysis of naturally occurring late flowering in *Arabidopsis thaliana*. Mol. Gen. Genet. **237:** 171-176.

Lee, I., S. D. Michaels, A. S. Masshardt and R. M. Amasino, 1994 The late-flowering phenotype of *FRIGIDA* and mutations in *LUMINIDEPENDENS* is suppressed in the Landsberg *erecta* strain of Arabidopsis. Plant J. **6:** 903-909.

Lee, I., and R. M. Amasino, 1995 Effect of vernalization, photoperiod, and light quality on the flowering phenotype of Arabidopsis plants containing the FRIGIDA gene. Plant Physiol. **108:** 157-162.

Meyer, R. C., O. Torjek, M. Becher and T. Altmann, 2004 Heterosis of biomass production in Arabidopsis. Establishment during early development. Plant Physiol. **134:** 1813-1823.

Michaels, S. D., and R. M. Amasino, 1999 FLOWERING LOCUS C encodes a novel MADS domain protein that acts as a repressor of flowering. Plant Cell **11:** 949-956.

Michaels, S. D., and R. M. Amasino, 2001 Loss of FLOWERING LOCUS C activity eliminates the late-flowering phenotype of FRIGIDA and autonomous pathway mutations but not responsiveness to vernalization. Plant Cell **13**: 935-942.
