# Supplementary material for: An Evaluation of Arabidopsis thaliana Hybrid Traits and Their Genetic Control
Source: G3 (Bethesda). 2011 Dec 1;1(7):571–9. doi: 10.1534/g3.111.001156 (PMC3276180; doi:10.1534/g3.111.001156)
Supplement: Supporting Information [file supp_1.7.571_TableS1.pdf]

Table S1 Reciprocal and non-maternal estimates for hybrids and maternal and SCA estimates of selfed parental lines from the diallel analysis

Trait	Diallel component	Hybrid Genotypes										Parental Genotypes				
		C24 x Col	C24 x Cvi	C24 x L*er*	C24 x Ws	Col x Cvi	Col x L*er*	Col x Ws	Cvi x L*er*	Cvi x Ws	L*er* x Ws	C24	Col	Cvi	L*er*	Ws
Days to bolting	REC	1.88*	0.00	-0.63	4.75**	0.13	0.00	-1.25	0.00	0.25	0.00	-	-	-	-	-
	MAT	-	-	-	-	-	-	-	-	-	-	1.20**	-0.60	0.03	0.13	-0.75*
	NMAT	0.08	-1.18	-1.70**	2.80**	0.75	0.73	-1.40*	0.10	-0.53	-0.88	-	-	-	-	-
	Selfed parent SCA	-	-	-	-	-	-	-	-	-	-	-20.04**	-6.24**	0.41	4.06**	-22.55**
Days to flowering	REC	1.88*	-0.25	-0.75	4.38**	-0.50	0.25	-1.50	-0.75	1.38	-0.13	-	-	-	-	-
	MAT	-	-	-	-	-	-	-	-	-	-	1.05**	-0.73*	0.28	0.23	-0.83*
	NMAT	0.10	-1.03	-1.58*	2.50**	0.50	1.20	-1.60*	-0.80	0.28	-1.18	-	-	-	-	-
	Selfed parent SCA	-	-	-	-	-	-	-	-	-	-	-19.62**	-6.32**	0.43	3.63**	-23.40**
Days to mature seed	REC	2.13*	-0.88	-0.13	5.00**	-0.38	-0.88	-1.42	-0.50	1.00	-0.50	-	-	-	-	-
	MAT	-	-	-	-	-	-	-	-	-	-	1.23**	-0.96*	0.35	0.20	-0.82*
	NMAT	-0.06	-1.75*	-1.15	2.96**	0.93	0.28	-1.28	-0.65	-0.17	-1.52*	-	-	-	-	-
	Selfed parent SCA	-	-	-	-	-	-	-	-	-	-	-20.87**	-6.66**	0.98	3.58**	-23.88**
Rosette diameter	REC	-1.25	-1.38	-5.38	16.38**	-2.88	2.00	2.63	1.38	-6.25	2.00	-	-	-	-	-
	MAT	-	-	-	-	-	-	-	-	-	-	1.68	0.60	-0.13	0.80	-2.95
	NMAT	-2.33	-3.18	-6.25	11.75**	-3.60	2.20	-0.93	2.30	-9.08**	-1.75	-	-	-	-	-
	Selfed parent SCA	-	-	-	-	-	-	-	-	-	-	-113.72**	-14.97**	-21.77**	3.78	-153.90**
Shoot biomass	REC	0.02	-0.04	0.05	-0.03	-0.04	-0.04	-0.02	-0.03	-0.02	0.02	-	-	-	-	-
	MAT	-	-	-	-	-	-	-	-	-	-	0.00	-0.02	0.01	0.01	0.01
	NMAT	-0.01	-0.03	0.05*	-0.02	-0.01	-0.01	0.01	-0.03	-0.01	0.02	-	-	-	-	-
	Selfed parent SCA	-	-	-	-	-	-	-	-	-	-	-0.45**	-0.03	-0.06	0.03	-0.55**
Final height	REC	2.13	2.88	2.00	13.63	-0.75	-14.75	-26.46	-15.25	-24.88	-10.38	-	-	-	-	-
	MAT	-	-	-	-	-	-	-	-	-	-	4.13	-8.82	-8.45	3.53	9.62
	NMAT	-10.82	-9.70	1.40	19.12	-0.38	-2.41	-8.03	-3.28	-6.81	-4.28	-	-	-	-	-
	Selfed parent SCA	-	-	-	-	-	-	-	-	-	-	-170.43**	11.86	-40.03*	-46.73*	-284.37**
Total siliques	REC	-33.88	3.13	48.75	-190.25*	-65.75	-101.00	-73.50	-82.63	-143.38	108.88	-	-	-	-	-
	MAT	-	-	-	-	-	-	-	-	-	-	-34.45	-41.28	-32.68	48.75	59.65
	NMAT	-40.70	4.90	131.95	-96.15	-57.15	-10.98	27.43	-1.20	-51.05	119.78	-	-	-	-	-
	Selfed parent SCA	-	-	-	-	-	-	-	-	-	-	-476.90**	146.10	-191.60	-98.05	-809.25**
Total seeds	REC[§]	-2.08	-0.62	0.90	-8.55*	-2.97	-3.50	-5.15	-5.50	-6.44	3.91	-	-	-	-	-
	MAT[§]	-	-	-	-	-	-	-	-	-	-	-2.07	-1.91	-1.67	2.40	3.25*
	NMAT[§]	-1.92	-0.21	5.34	-3.23	-2.74	0.81	0.01	-1.43	-1.53	4.75	-	-	-	-	-
	Selfed parent SCA[§]	-	-	-	-	-	-	-	-	-	-	-29.75**	7.13	-5.73	-9.92*	-48.68**
Silique length	REC	-0.30	-0.14	-0.15	-0.75**	-0.05	0.33	-0.65**	-0.85**	-0.13	0.21	-	-	-	-	-
	MAT	-	-	-	-	-	-	-	-	-	-	-0.27**	-0.02	-0.16	0.17	0.27**

	NMAT	-0.04	-0.03	0.30	-0.22	-0.19	0.51**	-0.37	-0.51**	0.29	0.30	-	-	-	-	-
	Selfed parent SCA	-	-	-	-	-	-	-	-	-	-	-1.74**	-0.87**	-1.25**	-3.20**	-8.02**
Seeds per silique	REC	-0.71	-0.78	-1.69	0.24	-0.64	2.64*	-1.97	-4.11**	-2.46	-2.30	-	-	-	-	-
	MAT	-	-	-	-	-	-	-	-	-	-	-0.59	0.15	-1.03*	0.17	1.30*
	NMAT	0.03	-1.22	-0.93	2.12*	-1.82	2.66**	-0.82	-2.90**	-0.13	-1.17	-	-	-	-	-
	Selfed parent SCA	-	-	-	-	-	-	-	-	-	-	-10.20**	-1.27	0.11	-11.12**	-26.56**
Height at flowering	REC	-6.25	-17.13*	-21.88**	-0.50	-12.25	-2.63	-17.54*	-1.13	9.88	0.88	-	-	-	-	-
	MAT	-	-	-	-	-	-	-	-	-	-	-9.15**	-5.23	7.63*	5.30	1.46
	NMAT	-2.33	-0.35	-7.43	10.11	0.61	7.91	-10.85	-3.45	3.71	-2.97	-	-	-	-	-
	Selfed parent SCA	-	-	-	-	-	-	-	-	-	-	17.65*	-4.69	4.35	-47.25**	-37.98
Lifespan	REC	-1.13	1.75	-5.00	1.50	7.25	2.25	-1.42	-7.63	-6.38	14.38*	-	-	-	-	-
	MAT	-	-	-	-	-	-	-	-	-	-	-0.58	1.84	-4.60	4.95*	-1.62
	NMAT	1.29	-2.28	0.53	0.46	0.81	5.36	-4.88	1.93	-3.39	7.81	-	-	-	-	-
	Selfed parent SCA	-	-	-	-	-	-	-	-	-	-	2.31	-3.13	-2.44	-9.24	-13.03

The REC and NMAT effects are given for the hybrid genotypes and the MAT and the SCA are given for the parental genotypes.

§ Value x 10^3

*** P < 0.05**

**** P < 0.01**
