# Supplementary material for: An Evaluation of Arabidopsis thaliana Hybrid Traits and Their Genetic Control
Source: G3 (Bethesda). 2011 Dec 1;1(7):571–9. doi: 10.1534/g3.111.001156 (PMC3276180; doi:10.1534/g3.111.001156)
Supplement: Supporting Information [file supp_1.7.571_TableS2.pdf]

Table S2 Raw data from diallel analysis

	Rep	Density	Block	(DAS) Bolting	(mm) Rosette Diameter	(DAS) Flowering	(mm) Flowering Height	(DAS) Mature Pod	Average Silique Length	Average # Seeds per Pod	(mm) Final Height	Total Siliques	(days) Lifespan	(g) Stem Dry Weight	Estimated Total Seeds
COL_P	1	1	1	19	51	23	41	37	12.08	48	352	1100	132	0.206	52800
COLxCOL	1	1	1	35	92	39	95	53	10.76	36.3	372	1294	146	0.211	46972.2
COLxLER	1	1	1	19	51	23	57	37	13.32	51.65	239	623	128	0.11	32177.95
COLxWS	1	1	1	19	48	24	112	38	10.88	41.2	304	939	140	0.104	38686.8
COLxCVI	1	1	1	23	42	25	79	38	14	37.65	288	898	132	0.122	33809.7
COLxC24	1	1	1	46	181	51	40	67	12.24	41.5	455	886	98	0.654	36769
LER_P	1	1	1	19	48	25	39	39	9.44	44.7	228	597	135	0.153	26685.9
LERxCOL	1	1	1	19	39	25	67	46	12.48	45.5	391	898	104	0.255	40859
LERxLER	1	1	1	19	36	23	48	37	8.8	39.25	171	320	115	0.084	12560
LERxWS	1	1	1	19	57	23	128	37	11.44	44.55	280	575	145	0.108	25616.25
LERxCVI	1	1	1	19	51	24	88	38	14.84	42.15	352	748	132	0.107	31528.2
LERxC24	1	1	1	27	95	32	152	46	13.52	50.75	480	1032	93	0.37	52374
WS_P	1	1	1	19	47	23	103	37	10.36	37.15	271	817	149	0.053	30351.55
WSxCOL	1	1	1	24	36	28	101	40	12.72	39.1	357	857	126	0.175	33508.7
WSxLER	1	1	1	19	58	23	111	37	12.32	48.2	332	495	137	0.12	23859
WSxWS	1	1	1	19	46	23	84	37	10.92	37.95	303	782	141	0.119	29676.9
WSxCVI	1	1	1	19	55	22	77	35	12.2	36.2	300	1170	153	0.149	42354
WSxC24	1	1	1	35	138	39	69	54	13.72	46.9	464	1557	126	0.815	73023.3
CVI_P	1	1	1	27	67	30	81	44	13.6	30.8	260	561	140	0.132	17278.8
CVIxCOL	1	1	1	19	45	25	94	38	13.12	35.25	329	901	131	0.2	31760.25
CVIxLER	1	1	1	19	54	22	111	36	11.92	33.9	308	343	132	0.093	11627.7
CVIxWS	1	1	1	19	30	26	93	38	11.12	27.45	231	469	149	0.031	12874.05
CVIxCVI	1	1	1	23	44	28	109	44	12.08	24.85	257	562	133	0.185	13965.7
CVIxC24	1	1	1	43	179	46	80	61	15.48	42.1	408	1301	140	0.707	54772.1
C24_P	1	1	1	28	53	32	85	46	12.44	39.15	305	1197	132	0.237	46862.55
C24xCOL	1	1	1	51	149	55	35	69	11.36	40.8	443	958	119	0.533	39086.4
C24xLER	1	1	1	22	65	27	90	42	14.52	49.7	383	897	130	0.419	44580.9
C24xWS	1	1	1	38	154	41	71	57	12.96	46.9	466	1251	132	0.564	58671.9
C24xCVI	1	1	1	39	174	43	92	55	15.44	41.1	430	1583	139	0.601	65061.3
C24xC24	1	1	1	28	52	32	111	46	12.8	36.8	351	1106	133	0.246	40700.8
COL_P	1	2	2	21	35	28	82	40	11.44	39.1	302	568	131	0.062	22208.8
COLxCOL	1	2	2	19	51	24	53	37	11	43.3	297	791	128	0.108	34250.3
COLxLER	1	2	2	19	57	24	131	37	13.08	56.25	196	383	140	0.061	21543.75
COLxWS	1	2	2	19	49	24	77.66667	37.667	11.76	40.2333	286.3333	749	133.67	0.108333	29553.4
COLxCVI	1	2	2	19	43	24	47	37	13.88	37.5	284	480	142	0.115	18000
COLxC24	1	2	2	46	122	51	41	66	13.52	47.05	403	750	94	0.291	35287.5
LER_P	1	2	2	19	43	23	46	37	8.48	34.75	205	168	97	0.055	5838
LERxCOL	1	2	2	19	49	24	91	37	12.76	52.35	162	491	133	0.101	25703.85
LERxLER	1	2	2	20	46	28	116	44	7.32	33.35	80	58	94	0.052	1934.3
LERxWS	1	2	2	19	53	25	154	38	12.32	42.5	316	416	142	0.119	17680
LERxCVI	1	2	2	20	45	25	99	38	13.8	39.1	247	137	130	0.053	5356.7
LERxC24	1	2	2	27	87	31	127	46	13.44	53.45	251	627	156	0.161	33513.15
WS_P	1	2	2	19	40	22	89	36	8.72	35.65	259	128	66	.	4563.2

WSxCOL	1	2	2	21	45	27	111	40	13.32	47.9	358	795	137	0.104	38080.5
WSxLER	1	2	2	19	50	24	129	37	11.4	51.45	268	339	106	0.031	17441.55
WSxWS	1	2	2	19	44	23	116	37	9.56	33.7	265	338	138	0.042	11390.6
WSxCVI	1	2	2	19	52	22.5	76	36	11.68	34.55	316.5	709.5	129	0.106	25273.05
WSxC24	1	2	2	34	128	38	86	53	13.8	48.3	451	1393	142	0.495	67281.9
CVI_P	1	2	2	24	48	29	113	42	11.2	28.2	266	184	146	0.029	5188.8
CVIxCOL	1	2	2	20	51	26	106	40	13.24	38.2	270	408	133	0.078	15585.6
CVIxLER	1	2	2	19	52	23	110	37	12.36	37.95	301	167	105	0.031	6337.65
CVIxWS	1	2	2	19	46	25	85	38	13.32	35.2	298	436	84	0.063	15347.2
CVIxCVI	1	2	2	23	42	28	128	41	12.52	30.5	253	185	105	0.091	5642.5
CVIxC24	1	2	2	38	139	41	83	58	14.88	37.85	356	1107	131	0.543	41899.95
C24_P	1	2	2	24	47	29	107	42	11.6	32.45	300	504	139	0.031	16354.8
C24xCOL	1	2	2	46	158	50	30	67	12.64	47.7	476	1001	132	0.595	47747.7
C24xLER	1	2	2	27	97	32	110	47	14.08	57.7	391	1170	99	0.451	67509
C24xWS	1	2	2	40	137	43	78	58	12.84	48.45	488	1076	126	0.446	52132.2
C24xCVI	1	2	2	48	189	51	40	67	14.44	41.3	416	888	131	0.576	36674.4
C24xC24	1	2	2	24	54	28	71	43	12.48	36.8	288	670	132	0.107	24656
COL_P	2	1	3	20	46	25	65	40	12.6	40.2	408	1199	128	0.303	48199.8
COLxCOL	2	1	3	19	44	23	55	37	11	40.6	339	950	130	0.159	38570
COLxLER	2	1	3	19	45	24	81	39	12.52	52.7	267	379	134	0.071	19973.3
COLxWS	2	1	3	19	47	24	49	37	12.96	37.2	291	1050	134	0.178	39060
COLxCVI	2	1	3	21	47	26	73	39	13.32	37.5	386	934	125	0.196	35025
COLxC24	2	1	3	44	178	46	58	60	12.12	48.15	465	1178	126	0.657	56720.7
LER_P	2	1	3	20	42	26	53	41	9.2	40.4	153	575	111	0.086	23230
LERxCOL	2	1	3	19	52	23	135	38	12.6	45.75	306	926	130	0.206	42364.5
LERxLER	2	1	3	19	40	22	46	36	9	41.2	241	342	101	0.107	14090.4
LERxWS	2	1	3	19	49	24	117	37	12.32	41.9	231	753	132	0.115	31550.7
LERxCVI	2	1	3	19	68	23	80	39	14.2	42.1	328	832	129	0.213	35027.2
LERxC24	2	1	3	27	104	32	124	46	15	58.6	439	1462	125	0.455	85673.2
WS_P	2	1	3	19	45	24	104	39	10.6	36.35	227	623	133	0.087	22646.05
WSxCOL	2	1	3	19	46	25	100	39	13.28	46.6	296	1306	134	0.236	60859.6
WSxLER	2	1	3	19	52	23	136	39	10.84	45.1	245	259	71	0.051	11680.9
WSxWS	2	1	3	19	46	22	105	36	10.36	36.4	252	567	140	0.087	20638.8
WSxCVI	2	1	3	19	49	23	75	37	11.16	32.9	333	249	105	0.063	8192.1
WSxC24	2	1	3	29	114	33	102	47	14.64	47.7	445	1507	111	0.482	71883.9
CVI_P	2	1	3	23	48	28	111	42	12.28	25.75	314	710	130	0.25	18282.5
CVIxCOL	2	1	3	22	65	27	104	40	14.52	40.45	339	1202	124	0.347	48620.9
CVIxLER	2	1	3	19	62	23	90	37	13.08	35	239	351	77	0.083	12285
CVIxWS	2	1	3	19	51	23	115	37	10.4	26.7	308	640	135	0.144	17088
CVIxCVI	2	1	3	26	52	30	83	45	12.64	29.3	277	629	106	0.243	18429.7
CVIxC24	2	1	3	39	179	44	157	60	13.96	40.25	484	1449	132	0.845	58322.25
C24_P	2	1	3	30	71	36	127	52	12.4	38.25	331	1153	106	0.279	44102.25
C24xCOL	2	1	3	53	160	56	30	71	12.08	43.6	429	737	106	0.585	32133.2
C24xLER	2	1	3	24	59	28	49	45	13.92	54.75	403	1284	138	0.317	70299
C24xWS	2	1	3	39	156	42	85	58	12.88	49.75	518	1178	131	0.5	58605.5
C24xCVI	2	1	3	39	180	42	65	58	15.04	40.2	431	1303	136	0.721	52380.6
C24xC24	2	1	3	26	51	28	80	43	11.88	33	339	1210	111	0.251	39930

COL_P	2	2	4	19	39	26	65	41	12.08	41.25	269	822	147	0.134	33907.5
COLxCOL	2	2	4	19	50	25	62	39	11.8	45.75	378	1110	120	0.282	50782.5
COLxLER	2	2	4	19	50	25	87	39	12.24	48.3	211	444	128	0.097	21445.2
COLxWS	2	2	4	19	52	24	72	38	11.44	42.3	264	258	127	0.043	10913.4
COLxCVI	2	2	4	21	51	26	103	41	12.96	35.85	294	468	154	0.096	16777.8
COLxC24	2	2	4	52	163	54	36	70	11.92	40.4	426	766	154	0.455	30946.4
LER_P	2	2	4	19	40	25	38	40	6.6	29.75	195	164	71	0.025	4879
LERxCOL	2	2	4	19	47	22	84	38	10.72	44.2	172	322	145	0.061	14232.4
LERxLER	2	2	4	19	37	23	43	38	7.08	27.6	188	159	114	0.055	4388.4
LERxWS	2	2	4	19	56	24	134	38	11.8	44.75	214	400	130	0.051	17900
LERxCVI	2	2	4	19	47	27	162	40	13.64	37.65	285	396	126	0.135	14909.4
LERxC24	2	2	4	27	88	32	101	47	13.84	54.75	409	804	132	0.157	44019
WS_P	2	2	4	19	43	25	78	40	9.32	33	160	162	99	0.03	5346
WSxCOL	2	2	4	22	48	28	139	43	12.92	43.1	346	626	149	0.116	26980.6
WSxLER	2	2	4	19	39	27	150	41	11.68	47.35	279	180	120	0.041	8523
WSxWS	2	2	4	19	51	23	96	38	10.24	34.85	292	303	98	0.026	10559.55
WSxCVI	2	2	4	19	52	22.5	76	36	11.68	34.55	316.5	709.5	129	0.106	25273.05
WSxC24	2	2	4	30	102	34	58	49	14.2	44.45	449	1459	130	0.423	64852.55
CVI_P	2	2	4	27	68	30	103	43	13.2	35.75	269	367	127	0.167	13120.25
CVIxCOL	2	2	4	22	45	27	96	40	13.64	39.7	320	795	107	0.204	31561.5
CVIxLER	2	2	4	20	54	25	109	41	12.36	21.3	242	591	142	0.09	12588.3
CVIxWS	2	2	4	21	31	27	90	39	10.84	29.15	230	146	97	0.033	4255.9
CVIxCVI	2	2	4	26	45	30	92	43	12.56	29.15	260	215	156	0.054	6267.25
CVIxC24	2	2	4	45	177	49	89	65	14.8	41.85	444	888	120	0.433	37162.8
C24_P	2	2	4	24	43	28	72	43	12.4	38.15	288	467	134	0.1	17816.05
C24xCOL	2	2	4	53	167	56	30	73	11.36	39.35	418	613	106	0.481	24121.55
C24xLER	2	2	4	30	110	34	80	50	12.12	41.9	418	964	99	0.334	40391.6
C24xWS	2	2	4	49	166	53	77	70	11.68	44.15	446	889	132	0.455	39249.35
C24xCVI	2	2	4	39	120	42	75	57	13.08	33.25	438	996	131	0.35	33117
C24xC24	2	2	4	24	43	28	79	43	11.76	38.05	290	456	134	0.087	17350.8
COLxWS	1	2	2	34	103	37	69	53	12.72	46.35	358	1130	154	.	52375.5
WSxCVI	1	2	2	20	53	27	118	41	11.68	44.4	303	436	136	0.041	19358.4
WSxCVI	2	2	4	19	49	24	72	38	11.32	38.85	298	198	114	0.032	7692.3

Col x Ws in block 2 has been substituted with the average from the Col x Ws genotypes in blocks 1, 3 and 4. Ws x Cvi in blocks 2 and 4 have been substituted with the averages of the Ws x Cvi genotypes in blocks 1 and 3. The original values of these three genotypes are listed at the end and shaded in grey. A density value of 1 indicates low density and a density value of 2 indicates high density.
