# Supplementary material for: An Evaluation of Arabidopsis thaliana Hybrid Traits and Their Genetic Control
Source: G3 (Bethesda). 2011 Dec 1;1(7):571–9. doi: 10.1534/g3.111.001156 (PMC3276180; doi:10.1534/g3.111.001156)
Supplement: Supporting Information [file supp_1.7.571_TableS3.pdf]

Table S3   Raw data for the number of seeds per silique averaged for the diallel experiment

BLOCK 1	1	2	3	4	5	6	7	8	9	10	11	12	13	14	15	16	17	18	19	20	Average # of Seed / Silique
Col Parent	20	49	39	49	49	40	58	52	54	55	37	51	61	53	47	41	42	53	55	55	48
Col x Col	36	46	17	37	18	40	24	18	41	39	44	58	40	27	33	28	47	36	52	45	36.3
Col x Ler	37	58	49	59	44	56	51	61	54	56	37	51	22	56	46	55	64	60	55	62	51.65
Col x Ws	24	41	24	38	39	46	40	30	33	44	38	33	56	43	50	49	47	57	46	46	41.2
Col x Cvi	41	47	32	39	43	40	29	33	27	32	44	29	35	45	42	40	37	39	34	45	37.65
Col x C24	56	54	59	54	48	32	23	35	46	48	20	44	32	40	43	38	36	43	35	44	41.5
Ler Parent	36	43	43	35	57	35	50	44	54	53	36	39	33	54	48	48	49	47	48	42	44.7
Ler x Col	48	49	44	39	57	48	42	37	38	51	67	55	45	37	46	50	46	37	30	44	45.5
Ler x Ler	25	10	50	27	35	37	31	51	35	36	48	42	51	50	46	12	54	47	46	52	39.25
Ler x Ws	53	32	45	51	50	50	46	44	36	40	34	50	43	48	56	43	42	48	42	38	44.55
Ler x Cvi	28	30	45	37	60	47	44	48	42	36	47	37	47	31	49	47	36	51	37	44	42.15
Ler x C24	23	57	65	60	49	44	58	52	44	30	63	54	48	55	62	51	55	45	57	43	50.75
Ws Parent	38	37	37	36	44	46	40	39	48	28	38	41	34	37	24	40	31	37	30	38	37.15
Ws x Col	36	44	42	56	24	49	38	46	38	42	27	55	14	41	39	38	29	31	47	46	39.1
Ws x Ler	41	35	44	58	46	58	54	36	27	52	55	48	56	46	57	42	52	50	53	54	48.2
Ws x Ws	41	24	27	41	37	35	33	42	34	41	50	39	35	32	50	35	47	31	48	37	37.95
Ws x Cvi	29	24	37	34	42	29	47	33	40	39	44	30	35	31	41	37	39	40	45	28	36.2
Ws x C24	45	41	15	51	47	50	39	52	45	52	51	50	45	54	56	47	51	47	51	49	46.9
Cvi Parent	9	19	26	34	39	46	29	48	34	28	40	32	37	33	29	23	24	30	14	42	30.8
Cvi x Col	33	27	39	13	40	41	44	31	35	36	39	40	38	40	28	35	41	39	24	42	35.25
Cvi x Ler	3	11	16	40	29	41	36	37	36	46	36	42	31	43	39	42	32	37	45	36	33.9
Cvi x Ws	27	21	25	34	32	28	34	28	27	29	19	20	31	26	32	33	25	30	20	28	27.45
Cvi x Cvi	39	12	11	30	9	24	19	28	10	24	22	28	18	29	31	44	32	36	24	27	24.85
Cvi x C24	53	40	39	49	42	37	40	37	40	44	33	41	48	48	37	39	37	45	47	46	42.1
C24 Parent	35	32	47	44	39	47	43	40	39	37	39	42	33	39	37	40	41	38	34	37	39.15
C24 x Col	57	21	28	20	51	50	50	55	20	45	43	37	39	52	34	47	36	49	45	37	40.8
C24 x Ler	16	58	40	57	52	59	60	57	65	43	46	36	32	61	53	56	50	58	50	45	49.7
C24 x Ws	50	46	58	35	37	52	31	47	39	43	48	47	52	39	51	58	58	50	48	49	46.9
C24 x Cvi	45	48	43	35	48	44	43	50	31	35	42	57	30	35	46	27	42	43	32	46	41.1
C24 x C24	23	33	35	38	40	42	40	43	41	31	41	29	37	46	42	34	35	43	29	34	36.8

BLOCK 2

	1	2	3	4	5	6	7	8	9	10	11	12	13	14	15	16	17	18	19	20	Average # of Seed / Silique
Col Parent	9	46	33	38	42	21	44	8	35	18	53	40	53	52	55	50	51	44	40	50	39.1
Col x Col	22	46	40	8	53	45	45	65	55	41	46	33	35	36	52	42	46	53	53	50	43.3
Col x Ler	57	57	60	61	63	39	43	57	59	52	44	57	55	53	62	66	57	61	64	58	56.25
Col x Ws	52	47	53	56	39	45	37	48	52	45	53	36	46	43	45	50	37	47	46	50	46.35
Col x Cvi	39	15	15	39	29	41	42	32	46	44	46	41	35	41	35	49	44	32	38	47	37.5
Col x C24	37	40	54	43	54	58	47	56	62	53	57	54	40	41	45	34	30	51	56	29	47.05
Ler Parent	52	39	46	38	30	36	40	38	28	29	36	32	39	34	8	39	40	32	24	35	34.75
Ler x Col	41	47	24	58	51	59	53	52	56	64	53	53	55	60	55	59	55	48	47	57	52.35
Ler x Ler	48	46	33	46	40	52	34	45	36	21	19	42	24	34	21	31	18	25	26	26	33.35
Ler x Ws	23	23	35	45	34	46	45	46	55	50	39	45	57	33	35	43	44	58	44	50	42.5
Ler x Cvi	35	30	33	46	49	40	46	47	53	22	26	36	47	45	46	37	35	37	32	40	39.1
Ler x C24	51	60	61	57	57	55	62	56	63	50	56	52	41	59	50	47	51	39	49	53	53.45
Ws Parent	33	34	37	19	31	26	37	37	35	33	27	22	42	36	45	47	38	38	55	41	35.65
Ws x Col	48	53	55	47	59	46	45	51	50	40	34	53	41	54	50	46	47	42	49	48	47.9
Ws x Ler	36	48	49	49	45	52	50	48	52	57	53	59	60	54	63	52	54	39	54	55	51.45
Ws x Ws	30	26	37	36	39	39	34	44	44	22	30	20	25	35	28	41	42	33	33	36	33.7
Ws x Cvi	44	47	44	45	42	48	48	50	42	40	41	49	40	45	42	41	43	49	38	50	44.4
Ws x C24	32	38	23	58	46	31	59	56	50	53	55	46	49	58	55	62	54	50	43	48	48.3
Cvi Parent	17	12	21	43	34	32	24	32	36	30	13	39	27	29	25	28	35	36	31	20	28.2
Cvi x Col	36	27	41	46	38	33	37	42	46	41	43	41	11	46	45	39	41	34	38	39	38.2
Cvi x Ler	38	38	38	32	39	40	35	45	36	38	37	39	48	39	25	42	45	43	30	32	37.95
Cvi x Ws	34	36	21	36	44	40	36	37	51	35	22	38	28	38	30	37	40	33	26	42	35.2
Cvi x Cvi	35	33	34	29	40	35	13	37	26	32	25	20	39	33	34	33	24	26	34	28	30.5
Cvi x C24	24	41	42	42	40	25	24	44	34	36	41	44	29	44	47	38	49	41	29	43	37.85
C24 Parent	40	38	28	40	44	31	33	22	27	30	32	29	26	34	35	31	36	32	31	30	32.45
C24 x Col	45	48	48	51	34	47	34	39	55	56	54	54	51	40	44	55	49	53	48	49	47.7
C24 x Ler	63	58	64	56	65	53	50	58	58	59	50	56	54	56	53	62	56	60	63	60	57.7
C24 x Ws	51	49	57	55	52	50	56	55	51	48	34	50	39	51	47	43	45	45	47	44	48.45
C24 x Cvi	39	26	43	53	49	19	33	49	50	45	43	33	54	48	34	45	42	45	35	41	41.3
C24 x C24	44	42	22	47	44	45	43	45	30	36	32	26	27	29	33	34	38	43	37	39	36.8

BLOCK 3

	1	2	3	4	5	6	7	8	9	10	11	12	13	14	15	16	17	18	19	20	Average # of Seed / Silique
Col Parent	4	8	3	48	48	43	45	45	44	44	56	41	52	54	45	43	42	39	54	46	40.2

Col x Col	37	35	34	43	42	38	36	63	48	52	43	49	45	16	36	39	40	44	34	38	40.6
Col x Ler	54	64	23	56	4	65	37	57	65	43	61	53	63	63	63	64	63	42	54	60	52.7
Col x Ws	31	43	32	36	9	54	22	44	48	20	21	52	37	55	33	26	42	39	51	49	37.2
Col x Cvi	56	36	40	18	31	49	33	49	32	22	36	39	34	37	44	45	38	41	33	37	37.5
Col x C24	59	51	50	51	53	57	39	48	47	54	42	48	48	47	46	55	45	46	42	35	48.15
Ler Parent	21	34	49	36	44	44	47	38	42	40	39	26	43	43	50	44	46	49	31	42	40.4
Ler x Col	54	29	49	38	45	49	43	41	49	40	26	49	43	48	50	51	58	54	46	53	45.75
Ler x Ler	70	35	41	30	45	40	41	41	46	43	34	41	41	43	47	29	40	35	47	35	41.2
Ler x Ws	48	45	44	34	41	30	43	45	34	27	43	41	49	37	35	55	45	46	55	41	41.9
Ler x Cvi	44	33	44	34	21	51	45	45	42	43	44	47	50	35	40	50	38	42	51	43	42.1
Ler x C24	45	64	49	58	58	62	62	62	64	68	63	51	53	61	61	53	58	69	66	45	58.6
Ws Parent	34	36	38	37	32	27	36	34	42	37	40	35	35	36	34	41	33	46	33	41	36.35
Ws x Col	18	23	32	49	47	52	53	54	56	56	50	45	50	54	56	41	57	48	44	47	46.6
Ws x Ler	35	47	38	41	26	47	56	54	45	44	46	45	56	56	40	49	46	50	47	34	45.1
Ws x Ws	36	45	25	40	28	38	40	29	45	36	24	37	35	35	45	39	41	39	30	41	36.4
Ws x Cvi	35	40	32	35	22	39	33	38	37	18	24	31	27	37	34	28	30	43	38	37	32.9
Ws x C24	45	49	32	37	57	50	36	51	38	52	42	48	43	48	59	45	59	50	52	61	47.7
Cvi Parent	21	22	35	27	19	29	28	31	28	15	25	30	16	36	14	43	29	26	25	16	25.75
Cvi x Col	27	46	41	40	38	42	46	43	47	32	37	48	41	52	35	46	38	39	29	42	40.45
Cvi x Ler	18	25	50	17	41	7	41	30	40	17	35	37	39	47	41	37	39	45	49	45	35
Cvi x Ws	21	16	27	30	30	28	27	38	24	35	19	21	38	33	25	20	32	17	23	30	26.7
Cvi x Cvi	38	23	31	34	41	32	34	22	14	10	19	21	20	32	32	28	42	36	38	39	29.3
Cvi x C24	41	38	25	32	47	45	30	39	23	43	49	40	43	40	43	44	51	41	45	46	40.25
C24 Parent	45	46	33	32	35	33	45	40	21	34	41	37	41	45	43	31	43	44	41	35	38.25
C24 x Col	54	46	44	36	55	33	26	25	28	52	45	47	51	31	52	46	52	59	43	47	43.6
C24 x Ler	53	55	62	63	55	65	65	53	34	39	59	58	50	51	42	64	56	54	58	59	54.75
C24 x Ws	57	52	56	50	60	54	57	17	27	54	58	57	50	61	37	47	55	50	45	51	49.75
C24 x Cvi	38	26	43	25	52	41	42	41	47	36	39	35	33	46	31	47	45	44	51	42	40.2
C24 x C24	40	21	42	15	38	16	41	39	40	23	30	24	40	30	29	35	41	37	37	42	33

**BLOCK 4**	1	2	3	4	5	6	7	8	9	10	11	12	13	14	15	16	17	18	19	20	**Average # of Seed / Silique**
Col Parent	4	33	23	42	26	61	54	47	30	45	31	15	48	57	41	56	54	57	50	51	41.25
Col x Col	19	47	39	48	53	53	56	56	48	57	54	56	31	51	18	46	44	42	51	46	45.75
Col x Ler	42	51	45	45	39	38	50	58	56	50	53	48	49	50	54	53	47	43	43	52	48.3
Col x Ws	24	37	37	31	34	28	47	53	47	48	46	49	48	51	47	46	43	43	44	43	42.3

Col x Cvi	23	45	11	28	42	27	48	42	42	35	56	40	19	44	28	42	39	31	34	41	35.85
Col x C24	26	22	43	47	33	39	35	25	41	38	43	47	54	49	41	46	45	46	38	50	40.4
Ler Parent	38	33	28	33	43	27	34	38	39	25	26	37	10	28	22	30	23	31	14	36	29.75
Ler x Col	30	50	28	45	44	53	46	54	34	44	55	53	56	35	45	48	41	36	53	34	44.2
Ler x Ler	37	37	21	34	27	26	34	33	28	20	31	28	27	29	21	28	28	14	24	25	27.6
Ler x Ws	48	53	41	44	36	33	37	49	48	50	45	46	44	50	37	35	40	58	57	44	44.75
Ler x Cvi	16	17	38	38	40	43	28	40	34	44	38	45	41	44	47	42	41	43	46	28	37.65
Ler x C24	54	50	59	63	66	59	62	64	65	45	53	55	54	49	46	46	50	49	54	52	54.75
Ws Parent	34	34	30	32	40	31	24	33	34	35	34	38	37	25	34	32	29	37	35	32	33
Ws x Col	19	36	50	42	46	42	52	52	48	45	51	52	38	49	25	46	47	45	40	37	43.1
Ws x Ler	8	47	38	47	53	43	57	60	38	55	56	54	39	57	47	50	59	48	51	40	47.35
Ws x Ws	36	4	26	20	34	37	44	42	45	39	36	46	41	45	30	34	42	31	31	34	34.85
Ws x Cvi	37	35	27	48	41	31	43	42	46	38	33	40	29	32	32	41	48	46	44	44	38.85
Ws x C24	21	46	31	41	28	49	36	48	54	57	51	57	50	33	54	50	54	34	46	49	44.45
Cvi Parent	34	43	39	40	30	28	33	27	41	39	32	45	45	41	44	24	31	31	33	35	35.75
Cvi x Col	37	44	45	39	38	34	41	40	40	45	35	34	43	33	39	36	40	40	43	48	39.7
Cvi x Ler	2	21	21	24	34	19	27	22	9	28	21	26	19	22	16	33	15	28	17	22	21.3
Cvi x Ws	28	26	43	32	34	29	28	27	22	27	29	30	24	27	36	24	29	29	37	22	29.15
Cvi x Cvi	14	21	9	16	38	27	35	36	23	39	22	36	34	40	37	34	29	43	27	23	29.15
Cvi x C24	42	56	44	38	41	47	41	47	40	47	45	47	38	40	35	37	39	35	38	40	41.85
C24 Parent	16	45	52	45	38	36	35	40	24	39	41	43	43	29	42	41	30	48	40	36	38.15
C24 x Col	30	42	28	38	48	37	35	42	32	45	41	27	37	41	51	40	41	50	38	44	39.35
C24 x Ler	35	33	51	47	46	54	35	36	42	60	44	46	52	44	35	37	41	26	40	34	41.9
C24 x Ws	39	46	53	45	43	34	45	38	33	40	49	38	41	53	48	49	51	50	45	43	44.15
C24 x Cvi	33	36	34	36	35	23	36	28	30	31	26	34	36	34	29	39	37	36	38	34	33.25
C24 x C24	17	44	45	40	24	56	34	40	32	40	31	50	36	36	47	43	35	42	31	38	38.05
