# Supplementary material for: An Evaluation of Arabidopsis thaliana Hybrid Traits and Their Genetic Control
Source: G3 (Bethesda). 2011 Dec 1;1(7):571–9. doi: 10.1534/g3.111.001156 (PMC3276180; doi:10.1534/g3.111.001156)
Supplement: Supporting Information [file supp_1.7.571_TableS4.pdf]

Table S4 Raw data for the average silique length in the diallel experiment

BLOCK 1	1	2	3	4	5	6	7	8	9	10	11	12	13	14	15	16	17	18	19	20	21	22	23	24	25	Average Silique length
Col Parent	12	12	11	13	12	12	12	12	12	13	12	12	13	13	11	12	11	13	11	12	12	13	12	12	12	12.08
Col x Col	12	11	12	10	10	9	8	11	10	13	12	10	12	10	10	10	11	11	10	12	11	11	10	12	11	10.76
Col x Ler	12	14	15	15	13	14	14	14	14	13	15	15	13	12	13	13	13	13	12	14	13	12	12	13	12	13.32
Col x Ws	10	11	11	11	10	10	10	12	12	11	11	10	11	11	12	11	12	11	11	10	11	12	10	11	10	10.88
Col x Cvi	13	15	16	14	14	16	13	12	14	15	15	12	13	14	15	13	14	15	15	14	14	16	13	11	14	14
Col x C24	14	13	15	13	14	14	10	13	9	8	11	12	12	13	11	12	11	14	10	12	10	13	13	15	14	12.24
Ler Parent	10	10	10	9	9	10	9	10	10	10	8	9	8	9	8	10	10	10	10	10	9	10	9	9	10	9.44
Ler x Col	15	12	14	13	14	14	12	14	13	11	14	12	11	12	13	13	14	11	11	11	12	11	11	12	12	12.48
Ler x Ler	11	10	10	9	10	11	9	9	9	9	8	8	10	9	7	9	7	8	9	9	8	8	8	7	8	8.8
Ler x Ws	12	11	10	12	9	12	11	13	11	11	13	13	13	13	12	10	14	14	11	12	9	9	11	10	10	11.44
Ler x Cvi	15	13	13	16	16	15	14	16	15	15	14	16	15	15	16	15	15	15	16	13	15	14	12	16	16	14.84
Ler x C24	11	16	16	16	13	16	13	13	14	16	14	12	13	14	13	13	14	13	14	13	13	11	12	13	12	13.52
Ws Parent	11	10	11	12	11	10	10	9	12	10	7	12	11	10	11	12	12	11	9	8	10	10	10	10	10	10.36
Ws x Col	12	12	13	10	15	10	14	13	13	12	15	11	11	12	13	13	14	14	13	13	13	13	14	12	13	12.72
Ws x Ler	14	14	11	12	12	12	11	13	14	10	12	14	11	11	11	12	13	12	13	12	11	13	14	12	14	12.32
Ws x Ws	11	12	11	11	9	10	11	11	11	10	12	13	10	11	12	11	10	11	10	10	11	12	10	12	11	10.92
Ws x Cvi	14	14	11	13	12	11	13	12	12	12	11	11	14	11	12	11	12	11	14	12	14	13	13	13	9	12.2
Ws x C24	14	14	11	14	13	15	14	15	15	14	13	14	14	14	15	13	13	14	13	13	15	13	13	13	14	13.72
Cvi Parent	12	15	14	16	17	13	16	15	16	16	14	11	12	8	11	14	12	15	11	12	14	16	15	12	13	13.6
Cvi x Col	13	13	16	10	13	14	14	12	13	15	13	14	14	13	14	12	12	12	14	13	14	12	14	12	12	13.12
Cvi x Ler	14	14	12	11	15	12	12	12	11	12	10	14	11	11	11	11	12	10	10	11	13	14	14	10	11	11.92
Cvi x Ws	11	12	11	12	13	8	12	12	13	12	12	11	12	11	9	11	12	11	11	10	11	11	10	10	10	11.12
Cvi x Cvi	19	12	12	11	8	9	13	13	11	13	8	10	11	15	16	12	13	11	12	12	11	10	13	15	12	12.08
Cvi x C24	17	16	16	18	15	15	15	16	15	16	14	16	15	14	15	15	15	14	16	15	16	16	15	15	17	15.48
C24 Parent	15	14	13	15	13	12	13	12	13	12	13	13	11	10	12	12	11	12	12	11	11	12	14	13	12	12.44
C24 x Col	13	10	11	12	11	12	10	13	8	10	12	11	9	12	12	13	10	12	11	13	11	12	12	12	12	11.36
C24 x Ler	18	16	16	18	18	15	16	17	16	12	14	15	15	15	14	13	14	13	14	12	13	12	14	11	12	14.52
C24 x Ws	15	16	14	14	14	13	12	11	14	13	13	13	14	13	13	12	13	14	12	12	13	11	13	10	12	12.96
C24 x Cvi	17	17	17	15	16	16	17	16	13	14	15	16	13	13	14	13	17	15	15	14	18	17	17	16	15	15.44
C24 x C24	16	16	14	14	13	14	12	12	13	13	12	13	13	14	13	12	11	12	12	11	12	12	12	13	11	12.8

BLOCK 2

	1	2	3	4	5	6	7	8	9	10	11	12	13	14	15	16	17	18	19	20	21	22	23	24	25	**Average Silique length**
Col Parent	11	13	11	7	9	13	10	14	13	13	13	13	12	11	12	12	12	11	12	11	10	10	11	11	11	11.44
Col x Col	11	11	12	8	11	11	12	11	12	11	13	12	13	12	9	10	10	10	11	11	12	11	9	11	11	11
Col x Ler	15	13	13	10	11	14	14	15	13	15	14	13	14	14	16	14	13	12	12	12	12	11	11	12	14	13.08
Col x Ws	15	12	16	14	13	11	11	13	12	11	14	10	13	14	12	12	14	10	11	14	14	12	14	12	14	12.72
Col x Cvi	16	15	14	13	14	13	15	15	15	16	12	13	11	14	12	14	14	14	14	13	13	16	14	14	13	13.88
Col x C24	15	13	16	14	15	12	14	16	14	16	14	15	15	13	15	15	10	14	12	11	9	10	13	15	12	13.52
Ler Parent	9	9	9	6	9	7	9	10	9	9	10	8	8	8	7	8	10	9	8	8	9	9	7	9	8	8.48
Ler x Col	12	12	12	14	13	13	14	14	13	13	13	15	13	14	12	12	14	13	12	12	12	12	12	11	12	12.76
Ler x Ler	10	10	10	10	10	11	9	9	7	6	5	7	7	6	5	7	5	5	5	8	5	4	.	.	.	7.32
Ler x Ws	13	13	13	11	13	15	13	13	11	13	12	15	12	14	12	11	11	14	12	12	10	13	11	11	10	12.32
Ler x Cvi	15	14	14	15	14	16	15	15	15	11	14	15	15	15	12	13	13	12	14	15	15	12	13	12	11	13.8
Ler x C24	15	15	12	14	12	14	13	13	14	13	13	13	14	13	14	14	15	14	13	13	13	15	13	12	12	13.44
Ws Parent	8	8	9	9	9	10	9	10	9	9	10	10	8	9	8	9	7	8	8	10	8	7	9	9	8	8.72
Ws x Col	14	13	14	13	14	13	13	14	14	13	15	15	12	9	13	13	12	15	14	14	13	13	13	14	13	13.32
Ws x Ler	13	12	13	11	14	9	13	12	11	12	13	10	13	11	11	11	12	12	10	9	11	11	10	11	10	11.4
Ws x Ws	10	9	10	10	9	8	7	10	11	11	10	9	11	10	10	8	10	10	10	9	9	11	10	8	9	9.56
Ws x Cvi	12	11	11	12	12	12	12	11	11	11	12	12	13	13	12	13	12	11	11	12	11	11	11	11	12	11.68
Ws x C24	13	15	10	15	12	15	14	13	13	15	15	13	11	12	13	13	14	13	13	15	17	15	17	15	14	13.8
Cvi Parent	9	11	15	14	13	12	12	13	11	8	14	12	12	10	11	10	10	12	11	11	10	10	11	9	9	11.2
Cvi x Col	14	15	14	14	15	13	14	15	8	14	14	13	12	14	14	13	13	13	13	13	13	13	12	12	13	13.24
Cvi x Ler	11	12	11	13	13	12	12	11	13	11	13	15	11	11	15	11	12	15	11	11	10	11	14	15	15	12.36
Cvi x Ws	14	14	14	12	13	13	15	15	10	15	13	16	15	14	13	12	13	15	13	12	12	12	13	13	12	13.32
Cvi x Cvi	13	15	14	9	15	15	15	13	12	16	12	12	13	12	12	12	14	11	8	11	9	13	12	12	13	12.52
Cvi x C24	10	16	15	14	16	16	15	15	15	14	13	15	14	16	16	14	15	16	16	15	16	15	15	15	15	14.88
C24 Parent	13	15	14	14	14	11	12	11	12	11	12	13	13	12	12	11	10	10	11	10	10	10	10	8	11	11.6
C24 x Col	14	11	12	13	13	12	11	13	13	11	14	12	14	13	13	14	13	11	12	13	13	12	12	14	13	12.64
C24 x Ler	12	15	15	14	13	12	14	13	13	12	14	13	14	12	16	13	14	15	15	15	16	15	16	15	16	14.08
C24 x Ws	16	14	15	15	13	15	12	14	12	13	14	12	12	12	12	13	13	13	12	12	9	12	14	11	11	12.84
C24 x Cvi	14	15	11	10	16	15	16	16	17	16	15	15	13	14	16	15	14	13	13	15	14	16	14	14	14	14.44
C24 x C24	11	15	15	15	15	13	13	14	12	12	12	13	13	12	11	10	13	12	11	13	9	11	12	13	12	12.48

BLOCK 3

	1	2	3	4	5	6	7	8	9	10	11	12	13	14	15	16	17	18	19	20	21	22	23	24	25	**Average Silique Length**
Col Parent	13	12	15	13	12	13	14	13	14	12	11	13	11	12	14	13	13	12	11	13	12	12	12	12	13	12.6

Col x Col	13	11	12	11	12	12	12	9	11	12	8	10	11	13	10	12	8	12	11	12	12	9	11	12	9	11
Col x Ler	11	14	15	12	14	12	12	14	11	13	12	13	12	11	13	13	12	13	12	11	10	14	14	11	14	12.52
Col x Ws	11	12	15	14	13	14	14	13	13	14	14	13	12	11	13	13	13	14	12	12	12	13	13	13	13	12.96
Col x Cvi	10	13	13	16	13	14	15	15	16	14	14	13	16	12	12	9	13	13	13	14	14	13	14	14	10	13.32
Col x C24	12	14	13	13	12	10	14	12	12	13	12	12	13	13	14	12	11	12	12	12	9	8	12	13	13	12.12
Ler Parent	8	10	11	10	9	11	10	9	9	10	10	9	9	9	9	9	9	9	9	9	7	9	9	9	8	9.2
Ler x Col	13	12	14	15	15	13	13	13	13	12	14	13	12	13	14	12	12	11	14	11	11	11	11	11	12	12.6
Ler x Ler	11	10	10	8	9	9	9	9	10	9	10	8	8	8	8	8	10	8	10	9	9	9	10	8	8	9
Ler x Ws	12	10	11	14	14	15	13	14	12	13	13	10	13	11	14	13	12	11	13	12	12	11	11	12	12	12.32
Ler x Cvi	14	15	15	15	15	15	12	16	13	15	14	15	16	13	14	12	12	15	14	13	14	13	16	15	14	14.2
Ler x C24	14	16	15	16	16	15	15	15	15	15	15	15	16	16	16	16	14	13	14	15	15	13	13	16	16	15
Ws Parent	11	10	10	11	12	11	11	11	11	12	12	10	11	11	12	11	11	11	10	9	9	9	9	11	9	10.6
Ws x Col	13	14	13	15	15	13	15	14	14	15	13	12	13	14	12	12	13	13	15	14	12	13	12	12	11	13.28
Ws x Ler	12	12	12	13	11	10	13	11	11	10	10	13	12	8	8	8	13	12	13	12	10	11	8	9	9	10.84
Ws x Ws	11	9	12	12	10	10	10	11	11	11	11	11	10	10	10	10	10	11	10	10	9	10	9	12	9	10.36
Ws x Cvi	10	11	12	10	11	11	12	12	13	13	11	12	11	11	11	10	12	12	11	9	10	11	10	11	12	11.16
Ws x C24	15	14	16	15	14	15	14	15	14	17	14	15	16	14	13	14	15	13	15	14	15	15	15	15	14	14.64
Cvi Parent	14	10	13	11	11	13	12	14	13	9	11	12	11	10	15	9	14	10	12	16	15	14	13	13	12	12.28
Cvi x Col	15	15	16	16	13	15	15	13	15	14	15	15	15	15	14	14	15	15	14	14	13	15	14	14	14	14.52
Cvi x Ler	13	16	15	15	14	12	13	11	12	11	13	15	10	12	14	13	14	13	15	16	14	13	11	10	12	13.08
Cvi x Ws	8	11	11	11	12	9	11	11	11	12	11	9	10	10	11	11	10	10	11	12	11	10	8	9	10	10.4
Cvi x Cvi	15	13	11	12	15	14	16	13	10	12	13	11	8	10	11	13	13	16	12	14	12	14	13	11	14	12.64
Cvi x C24	14	13	12	12	15	16	13	13	10	14	14	14	14	15	15	14	15	13	14	15	14	12	16	16	16	13.96
C24 Parent	14	14	15	11	13	11	10	11	9	11	13	12	13	12	12	13	9	13	15	13	13	13	12	14	14	12.4
C24 x Col	15	13	11	13	8	10	10	8	15	14	12	13	13	14	9	12	11	13	13	13	11	12	12	13	14	12.08
C24 x Ler	16	15	14	15	15	15	15	14	14	14	13	12	13	13	14	14	13	14	13	14	13	14	14	14	13	13.92
C24 x Ws	13	14	16	15	15	14	14	15	14	11	9	12	13	14	11	11	13	12	12	13	11	12	13	14	11	12.88
C24 x Cvi	13	12	16	14	15	18	16	16	16	17	14	15	16	13	14	13	14	15	18	16	14	14	16	15	16	15.04
C24 x C24	11	16	13	10	14	13	10	14	10	12	12	10	12	12	12	12	13	12	11	10	11	12	12	11	12	11.88

**BLOCK 4**																										**Average**
	1	2	3	4	5	6	7	8	9	10	11	12	13	14	15	16	17	18	19	20	21	22	23	24	25	**Silique Length**
Col Parent	13	13	10	12	11	12	13	13	14	13	11	12	12	12	12	11	8	12	12	12	13	13	13	12	13	12.08
Col x Col	13	14	14	12	12	14	8	12	12	11	11	13	12	11	10	11	11	10	12	11	12	14	12	12	11	11.8
Col x Ler	13	13	13	13	13	13	11	12	11	12	12	12	12	13	13	12	12	12	12	11	12	13	12	13	11	12.24
Col x Ws	13	12	13	12	13	13	11	12	13	12	11	11	11	11	11	11	10	11	10	11	11	12	11	10	10	11.44

Col x Cvi	15	15	11	13	14	13	13	14	12	14	14	12	14	13	13	13	15	14	10	13	10	11	12	14	12	12.96
Col x C24	11	11	13	12	12	9	11	13	12	9	11	12	12	14	13	11	11	13	12	13	12	14	13	13	11	11.92
Ler Parent	8	8	7	7	8	8	7	8	7	7	6	6	5	8	5	6	7	7	6	6	5	4	7	7	5	6.6
Ler x Col	12	10	11	13	13	10	14	10	11	11	11	11	12	10	10	11	10	11	10	10	10	9	10	9	9	10.72
Ler x Ler	8	8	7	8	7	9	7	7	7	8	7	8	8	6	6	7	7	7	7	6	7	6	6	7	6	7.08
Ler x Ws	12	10	14	11	12	12	11	12	11	11	12	13	10	13	11	12	10	12	13	11	14	14	11	10	13	11.8
Ler x Cvi	13	14	13	14	15	15	13	16	15	15	13	14	15	13	15	10	14	14	14	14	14	13	10	13	12	13.64
Ler x C24	15	13	14	13	16	15	14	16	13	11	14	14	14	13	14	13	13	14	15	13	13	15	14	13	14	13.84
Ws Parent	10	11	9	10	9	8	10	11	10	10	9	10	10	9	8	8	9	8	9	9	11	8	9	8	10	9.32
Ws x Col	15	14	14	13	13	13	13	14	15	13	12	12	12	13	13	12	13	13	12	13	13	13	12	13	10	12.92
Ws x Ler	10	12	13	12	13	13	12	13	12	12	12	12	13	12	12	11	11	12	11	11	10	10	10	12	11	11.68
Ws x Ws	10	10	11	11	11	9	10	11	10	12	10	11	11	11	11	11	10	9	9	10	10	10	9	10	9	10.24
Ws x Cvi	13	13	14	14	12	13	11	12	11	11	12	12	11	11	11	8	11	12	11	11	10	9	11	9	10	11.32
Ws x C24	14	10	14	12	13	15	12	16	15	15	15	16	15	15	15	15	13	15	13	15	14	16	13	14	15	14.2
Cvi Parent	12	16	15	13	13	13	12	12	14	12	14	10	14	13	15	13	13	14	14	16	12	11	17	10	12	13.2
Cvi x Col	15	15	14	16	15	15	14	15	13	14	15	13	15	11	14	14	13	12	12	13	12	12	14	12	13	13.64
Cvi x Ler	9	10	13	8	13	13	15	13	13	13	13	15	10	15	13	14	12	12	11	13	14	14	12	9	12	12.36
Cvi x Ws	13	12	12	12	12	12	11	12	12	10	9	11	11	10	10	10	9	10	11	11	10	10	10	11	10	10.84
Cvi x Cvi	12	12	10	12	13	14	11	14	14	10	11	12	13	14	13	13	12	12	13	14	13	13	13	13	13	12.56
Cvi x C24	13	17	15	15	13	14	16	14	15	15	15	15	15	15	16	15	14	13	15	15	14	15	15	15	16	14.8
C24 Parent	13	14	15	15	14	13	12	12	11	12	13	12	10	12	13	12	12	12	12	12	13	11	12	11	12	12.4
C24 x Col	11	12	13	12	11	10	12	10	9	11	11	12	10	8	10	13	13	12	11	11	11	14	12	13	12	11.36
C24 x Ler	10	12	13	12	11	13	15	15	12	11	11	12	10	11	11	11	13	12	14	13	11	12	13	13	12	12.12
C24 x Ws	10	13	15	12	12	11	10	11	9	11	12	13	11	12	12	13	12	13	13	12	10	11	11	11	12	11.68
C24 x Cvi	14	13	14	11	13	13	14	13	14	13	13	12	14	13	14	13	12	13	12	13	12	14	13	13	14	13.08
C24 x C24	14	14	15	12	12	13	11	11	10	11	12	12	10	11	12	12	11	12	12	10	10	12	11	12	12	11.76

Silique length measurements are in millimeters.
