# Supplementary material for: An Evaluation of Arabidopsis thaliana Hybrid Traits and Their Genetic Control
Source: G3 (Bethesda). 2011 Dec 1;1(7):571–9. doi: 10.1534/g3.111.001156 (PMC3276180; doi:10.1534/g3.111.001156)
Supplement: Supporting Information [file supp_1.7.571_TableS5.pdf]

Table S5 Raw data from the *FRI* and *FLC* experiment

Genotype FRI/FLC	Block	Bolting Date (DAS)	Rosette Diameter (mm)	Flowering Date (DAS)	Flowering Height (mm)	Maturity Date (DAS)	Final Height (mm)	Lifespan (days)	Stem Weight (grams)	Average Silique Length	Average Number of Seeds per pod	Total Number of Siliques	Estimated Total Number of Seeds
Col-/-	1	19	44	24	49	36	430	137	0.567	14.8	52.8	953	50318.4
Col-/+	1	20	36	26	50	39	414	158	0.487	14.2	55.6	1031	57323.6
Col+/-	1	20	45	25	47	39	394	154	0.49	14.6	52.2	1088	56793.6
Col+/+	1	54	154	59	41	78	517	186	0.894	10.8	28.4	245	6958
Ler-/-	1	19	30	23	34	37	219	108	0.147	10.4	46.4	364	16889.6
Ler-/+C	1	21	42	27	25	44	245	148	0.354	12.8	54.4	825	44880
Ler-/+S	1	21	40	27	21	41	278	117	0.3	12	54.2	673	36476.6
Ler+/-	1	21	37	27	20	42	269	134	0.309	13	60.4	622	37568.8
Ler+/+	1	77	121	83	17	105	192	202	0.45	9.25	31.25	169	5281.25
C24	1	34	72	39	120	53	433	156	0.388	14.2	46	744	34224
C24xCol-/-	1	21	51	27	42	40	441	147	0.621	17.6	62.4	992	61900.8
C24xCol-/+	1	.	.	.	.	.	.	.	.	.	.	.	.
C24xLer-/-	1	23	67	27	42	42	432	150	0.396	17.4	58.8	732	43041.6
C24xLer-/+	1	46	146	52	41	68	548	183	0.8	17	54	978	52812
Col-/-	2	19	55	23	55	36	379	144	0.443	15.2	51.8	1063	55063.4
Col-/+	2	20	37	25	44	38	366	154	0.456	14.6	53.8	935	50303
Col+/-	2	21	36	26	51	37	401	145	0.567	13.6	49.4	979	48362.6
Col+/+	2	40	128	44	31	60	614	196	0.791	13.2	53.8	836	44976.8
Ler-/-	2	19	23	23	21	37	233	110	0.132	11.2	54.4	327	17788.8
Ler-/+C	2	20	34	25	16	40	255	140	0.285	11.2	49.2	749	36850.8
Ler-/+S	2	21	29	27	19	41	294	119	0.26	11.8	54.8	527	28879.6
Ler+/-	2	23	44	29	12	44	257	115	0.286	13.4	61.8	395	24411
Ler+/+	2	60	132	68	23	.	.	.	.	.	.	.	.
C24	2	34	66	37	123	52	310	140	0.456	11.4	25.6	996	25497.6
C24xCol-	2	25	70	30	70	43	448	151	0.609	16	55.6	1100	61160
C24xCol+	2	40	115	44	29	58	465	188	0.496	15.2	50.2	693	34788.6
C24xLer-	2	28	89	34	78	48	436	155	0.449	15.6	57.4	730	41902
C24xLer+	2	47	134	53	19	68	496	204	0.729	15.4	47.8	927	44310.6

Col-/-	3	20	41	25	43	37	389	143	0.521	14.6	49.6	1090	54064
Col-/+	3	21	45	27	39	40	395	153	0.436	15.4	57.8	991	57279.8
Col+/-	3	20	38	25	40	38	398	146	0.441	15	53	744	39432
Col+/+	3	44	126	48	11	68	676	176	0.947	11.8	28.8	406	11692.8
Ler-/-	3	19	28	23	36	37	220	70	0.106	10.6	47.2	234	11044.8
Ler-/+C	3	21	24	26	29	41	239	145	0.228	12.2	51	669	34119
Ler-/+S	3	30	71	35	80	50	263	118	0.299	11.4	57.6	692	39859.2
Ler+/-	3	30	72	35	92	50	236	144	0.34	12.8	63	814	51282
Ler+/+	3	67	107	73	14	93	294	182	0.357	10.6	49.8	389	19372.2
C24	3	33	69	37	126	51	413	139	0.319	14.4	46.2	700	32340
C24xCol-	3	24	74	29	27	43	472	155	0.752	16.8	57.2	1288	73673.6
C24xCol+	3	44	137	47	48	60	569	192	0.611	13.8	49.8	842	41931.6
C24xLer-	3	25	64	31	72	45	449	146	0.443	15	53.4	627	33481.8
C24xLer+	3	45	122	51	23	66	523	209	0.756	15.8	56.8	923	52426.4
Col-/-	4	19	43	23	29	37	385	144	0.493	14.4	52.4	783	41029.2
Col-/+	4	21	36	26	34	39	432	138	0.409	14.8	57.4	1001	57457.4
Col+/-	4	23	46	29	86	42	377	146	0.565	14.6	56.8	1179	66967.2
Col+/+	4	72	148	78	26	94	511	142	0.659	.	.	.	.
Ler-/-	4	19	26	23	39	37	238	95	0.075	10.8	45	224	10080
Ler-/+C	4	19	38	23	36	37	229	135	0.269	11.2	46.8	837	39171.6
Ler-/+S	4	20	25	25	28	38	277	121	0.253	10.2	45.2	643	29063.6
Ler+/-	4	27	62	33	28	49	260	133	0.418	13	66	943	62238
Ler+/+	4	67	121	75	33	91	258	183	0.335	11.2	52.6	455	23933
C24	4	36	72	41	134	54	414	145	0.381	14.4	47.4	648	30715.2
C24xCol-	4	23	64	29	49	42	461	149	0.784	18.6	62.6	1414	88516.4
C24xCol+	4	48	109	53	14	68	487	198	0.468	15.8	52	734	38168
C24xLer-	4	27	95	33	118	47	436	131	0.534	15.4	60	952	57120
C24xLer+	4	48	137	53	20	69	567	184	1.013	14.8	53.6	985	52796
Col-/-	5	19	48	24	42	37	337	134	0.389	15.6	56	1120	62720
Col-/+	5	20	46	25	48	39	378	148	0.487	13.2	50.6	1015	51359
Col+/-	5	20	44	25	34	39	383	144	0.437	14	51.6	988	50980.8
Col+/+	5	47	155	52	16	69	676	180	0.898	12.6	48	525	25200
Ler-/-	5	19	22	23	14	37	162	83	0.057	10	43.8	124	5431.2
Ler-/+C	5	21	31	27	18	42	245	141	0.266	11.6	52.4	816	42758.4

Ler-/+S	5	22	31	28	15	42	258	98	0.088	11.8	54.4	216	11750.4
Ler+/-	5	24	42	31	71	49	277	108	0.275	11.6	56.8	551	31296.8
Ler+/+	5	65	113	71	19	90	281	156	0.419	11.4	46.4	446	20694.4
C24	5	33	73	37	119	50	328	111	0.244	14.8	47.8	508	24282.4
C24xCol-	5	23	60	28	30	42	486	151	0.759	17.4	56.6	1047	59260.2
C24xCol+	5	51	142	55	31	69	547	182	1.069	13.6	47.2	1027	48474.4
C24xLer-	5	28	102	33	77	47	453	123	0.434	16.4	63.4	724	45901.6
C24xLer+	5	43	157	47	41	62	509	190	0.928	15	53.4	1086	57992.4
Col-/-	6	19	54	24	40	39	414	135	0.465	15.2	57.2	1116	63835.2
Col-/+	6	22	32	28	39	41	401	145	0.429	14.8	55.8	925	51615
Col+/-	6	21	35	26	45	39	357	129	0.45	13	48.4	869	42059.6
Col+/+	6	47	152	52	21	72	574	181	0.669	12.8	36	256	9216
Ler-/-	6	19	25	24	46	38	195	99	0.08	11	49	232	11368
Ler-/+C	6	21	31	27	17	42	202	140	0.219	11.4	47.6	632	30083.2
Ler-/+S	6	23	39	30	15	46	254	109	0.287	12.2	59.6	606	36117.6
Ler+/-	6	22	33	28	24	45	307	122	0.369	12.6	61	772	47092
Ler+/+	6	81	125	88	10	.	.	.	.	.	.	.	.
C24	6	35	76	40	168	53	396	141	0.394	14.6	43.6	810	35316
C24xCol-	6	28	102	33	73	43	469	139	0.73	16.4	58	1419	82302
C24xCol+	6	44	147	49	31	64	468	193	0.654	13.2	44.4	1001	44444.4
C24xLer-	6	28	62	33	52	47	503	148	0.596	17.4	64.8	1030	66744
C24xLer+	6	40	147	44	46	61	580	181	0.773	15.8	54	1077	58158

A period indicates that no data was available.
