# Supplementary material for: An Evaluation of Arabidopsis thaliana Hybrid Traits and Their Genetic Control
Source: G3 (Bethesda). 2011 Dec 1;1(7):571–9. doi: 10.1534/g3.111.001156 (PMC3276180; doi:10.1534/g3.111.001156)
Supplement: Supporting Information [file supp_1.7.571_TableS6.pdf]

Table S6 Raw data for the number of seeds per silique and silique length measurements in the *FRI* and *FLC* experiments

Genotype FRI/FLC	Block	Seeds per Silique 1	Seeds per Silique 2	Seeds per Silique 3	Seeds per Silique 4	Seeds per Silique 5	Average # seeds / Silique	Silique Length 1	Silique Length 2	Silique Length 3	Silique Length 4	Silique Length 5	Average Silique Length
Col-/-	1	16	15	15	15	13	14.8	59	47	56	58	44	52.8
Col-/+	1	15	14	14	15	13	14.2	57	63	52	57	49	55.6
Col+/-	1	16	16	13	13	15	14.6	46	55	61	46	53	52.2
Col+/+	1	11	13	12	9	9	10.8	21	35	29	28	29	28.4
Ler-/-	1	11	11	9	11	10	10.4	44	56	43	47	42	46.4
Ler-/+C	1	13	13	13	13	12	12.8	51	54	53	56	58	54.4
Ler-/+S	1	11	12	13	12	12	12.0	50	54	50	53	64	54.2
Ler+/-	1	13	13	13	13	13	13.0	59	60	62	59	62	60.4
Ler+/+	1	8	11	11	7	.	9.25	23	41	36	25	.	31.25
C24	1	16	15	14	13	13	14.2	51	44	46	50	39	46.0
C24xCol-/-	1	18	18	18	17	17	17.6	58	61	62	64	67	62.4
C24xCol-/+	1	.	.	.	.	.	.	.	.	.	.	.	.
C24xLer-/-	1	19	19	17	16	16	17.4	63	62	60	57	52	58.8
C24xLer-/+	1	18	17	18	17	15	17.0	54	57	58	55	46	54.0
Col-/-	2	16	15	16	15	14	15.2	49	51	50	57	52	51.8
Col-/+	2	15	14	14	15	15	14.6	50	51	52	57	59	53.8
Col+/-	2	15	15	12	13	13	13.6	45	66	42	50	44	49.4
Col+/+	2	15	13	13	12	13	13.2	66	55	53	48	47	53.8
Ler-/-	2	11	11	11	11	12	11.2	52	49	53	54	64	54.4
Ler-/+C	2	12	11	11	11	11	11.2	44	52	51	46	53	49.2
Ler-/+S	2	11	12	13	12	11	11.8	46	50	55	65	58	54.8
Ler+/-	2	14	14	13	13	13	13.4	68	64	52	65	60	61.8
Ler+/+	2	.	.	.	.	.	.	.	.	.	.	.	.
C24	2	12	12	11	11	11	11.4	25	26	27	23	27	25.6
C24xCol-	2	16	17	16	16	15	16.0	56	50	56	54	62	55.6
C24xCol+	2	15	16	16	15	14	15.2	51	49	50	48	53	50.2
C24xLer-	2	17	16	15	15	15	15.6	67	55	54	58	53	57.4
C24xLer+	2	16	17	17	14	13	15.4	47	58	51	43	40	47.8

Col-/-	3	13	16	15	15	14	14.6	39	50	55	56	48	49.6
Col-/+	3	16	16	15	16	14	15.4	60	55	58	56	60	57.8
Col+/-	3	15	16	16	14	14	15.0	47	57	56	49	56	53.0
Col+/+	3	12	12	12	11	12	11.8	26	25	33	30	30	28.8
Ler-/-	3	12	11	11	9	10	10.6	52	52	41	50	41	47.2
Ler-/+C	3	13	12	13	11	12	12.2	44	54	58	50	49	51.0
Ler-/+S	3	12	11	12	11	11	11.4	67	57	63	51	50	57.6
Ler+/-	3	12	13	13	13	13	12.8	51	64	74	60	66	63.0
Ler+/+	3	11	10	11	11	10	10.6	42	43	58	55	51	49.8
C24	3	15	15	15	14	13	14.4	46	46	50	43	46	46.2
C24xCol-	3	15	19	18	16	16	16.8	47	62	64	59	54	57.2
C24xCol+	3	14	14	13	13	15	13.8	42	51	48	52	56	49.8
C24xLer-	3	16	15	15	15	14	15.0	57	50	58	55	47	53.4
C24xLer+	3	18	17	16	14	14	15.8	62	57	58	51	56	56.8
Col-/-	4	17	14	13	14	14	14.4	54	48	49	54	57	52.4
Col-/+	4	15	15	14	15	15	14.8	54	51	62	56	64	57.4
Col+/-	4	15	15	15	14	14	14.6	59	62	51	54	58	56.8
Col+/+	4	.	.	.	.	.	.	.	.	.	.	.	.
Ler-/-	4	11	11	11	10	11	10.8	43	41	46	48	47	45.0
Ler-/+C	4	12	12	11	11	10	11.2	47	50	42	47	48	46.8
Ler-/+S	4	11	9	10	10	11	10.2	45	39	40	51	51	45.2
Ler+/-	4	14	13	12	14	12	13.0	66	63	65	71	65	66.0
Ler+/+	4	12	12	10	11	11	11.2	53	53	56	49	52	52.6
C24	4	16	13	15	14	14	14.4	52	43	47	50	45	47.4
C24xCol-	4	19	19	20	18	17	18.6	58	59	64	59	73	62.6
C24xCol+	4	16	16	16	15	16	15.8	56	52	58	50	44	52.0
C24xLer-	4	16	16	15	15	15	15.4	59	65	55	62	59	60.0
C24xLer+	4	16	16	15	13	14	14.8	57	57	51	46	57	53.6
Col-/-	5	17	15	16	15	15	15.6	59	60	58	53	50	56.0
Col-/+	5	14	13	13	13	13	13.2	45	51	54	50	53	50.6
Col+/-	5	15	15	14	13	13	14.0	57	46	53	48	54	51.6
Col+/+	5	14	13	13	12	11	12.6	44	57	58	45	36	48.0
Ler-/-	5	12	10	10	9	9	10.0	49	40	48	42	40	43.8
Ler-/+C	5	12	12	12	12	10	11.6	57	46	57	53	49	52.4

Ler-/+S	5	12	12	13	12	10	11.8	54	57	55	56	50	54.4
Ler+/-	5	13	13	13	10	9	11.6	62	59	64	52	47	56.8
Ler+/+	5	12	12	11	11	11	11.4	43	51	44	44	50	46.4
C24	5	17	15	14	15	13	14.8	51	56	46	43	43	47.8
C24xCol-	5	19	19	17	17	15	17.4	53	66	50	63	51	56.6
C24xCol+	5	15	15	13	13	12	13.6	51	51	46	45	43	47.2
C24xLer-	5	18	17	17	15	15	16.4	66	62	67	64	58	63.4
C24xLer+	5	16	15	15	15	14	15.0	63	50	50	57	47	53.4
Col-/-	6	17	14	15	16	14	15.2	59	43	51	69	64	57.2
Col-/+	6	15	14	15	15	15	14.8	58	47	60	57	57	55.8
Col+/-	6	12	14	13	13	13	13.0	42	50	50	48	52	48.4
Col+/+	6	13	13	13	12	13	12.8	27	36	40	31	46	36.0
Ler-/-	6	11	11	11	12	10	11.0	48	41	55	58	43	49.0
Ler-/+C	6	12	12	11	11	11	11.4	49	51	47	48	43	47.6
Ler-/+S	6	13	11	13	12	12	12.2	63	53	61	61	60	59.6
Ler+/-	6	12	13	13	13	12	12.6	46	72	65	69	53	61.0
Ler+/+	6	.	.	.	.	.	.	.	.	.	.	.	.
C24	6	17	15	15	13	13	14.6	49	49	42	43	35	43.6
C24xCol-	6	16	17	17	16	16	16.4	55	60	64	53	58	58.0
C24xCol+	6	13	13	14	13	13	13.2	34	49	53	45	41	44.4
C24xLer-	6	18	18	17	17	17	17.4	64	63	63	68	66	64.8
C24xLer+	6	15	17	16	15	16	15.8	41	60	58	58	53	54.0

Silique length measurements are in millimeters.
